# Brønsted acid and Pd–PHOX dual-catalysed enantioselective addition of activated C-pronucleophiles to internal dienes†
†Electronic supplementary information (ESI) available. See DOI: 10.1039/c9sc00633h


**DOI:** 10.1039/c9sc00633h

**Published:** 2019-04-17

**Authors:** Sangjune Park, Nathan J. Adamson, Steven J. Malcolmson

**Affiliations:** a Department of Chemistry , Duke University , French Family Science Center , 124 Science Drive , Durham , NC 27708 , USA . Email: steven.malcolmson@duke.edu

## Abstract

We describe the development of Pd–PHOX-catalysed enantioselective couplings of internal dienes with malononitrile and other activated C-pronucleophiles. Reactions are dramatically accelerated by the addition of Et_3_N·HBAr^F^_4_ as a Brønsted acid co-catalyst, enabling a suite of products bearing a variety of alkyl substituents at the stereogenic carbon to be prepared. A series of mechanism-oriented experiments reveal key aspects of the catalytic cycle and the importance of the non-coordinating BAr^F^_4_ counterion in not only promoting reactions of internal dienes but also additions of previously unreactive nucleophiles towards terminal dienes.

## Abstract 2

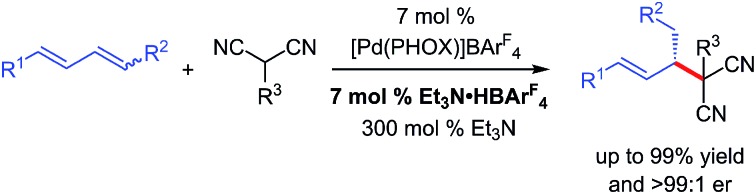
We describe the development of Pd–PHOX-catalysed enantioselective couplings of internal dienes with malononitrile and other activated C-pronucleophiles.

## Introduction

The addition of carbon-based electrophiles to enolates is a hallmark strategy in organic synthesis for the enantioselective formation of C–C bonds. Mannich reactions, aldol and Michael additions^1^ are among the most common approaches for the construction of small molecules. Enolate alkylations are also widespread but often focused towards controlling stereochemistry at a carbonyl's α-position.^2^ An important class of enolate alkylation is allylic substitution,^3^ which enables the assembly of molecules comprised of carbonyls with β-stereogenic centres^4^ while often concomitantly setting the stereochemistry at the α-position.^5^,^6^ Additionally, the products contain pendant unsaturation that might be leveraged for downstream synthesis applications. These enolate alkylations (allylic and non-allylic electrophiles) utilize *preactivated electrophiles* such as alkyl halides or allylic acetates.

**Scheme 1:**
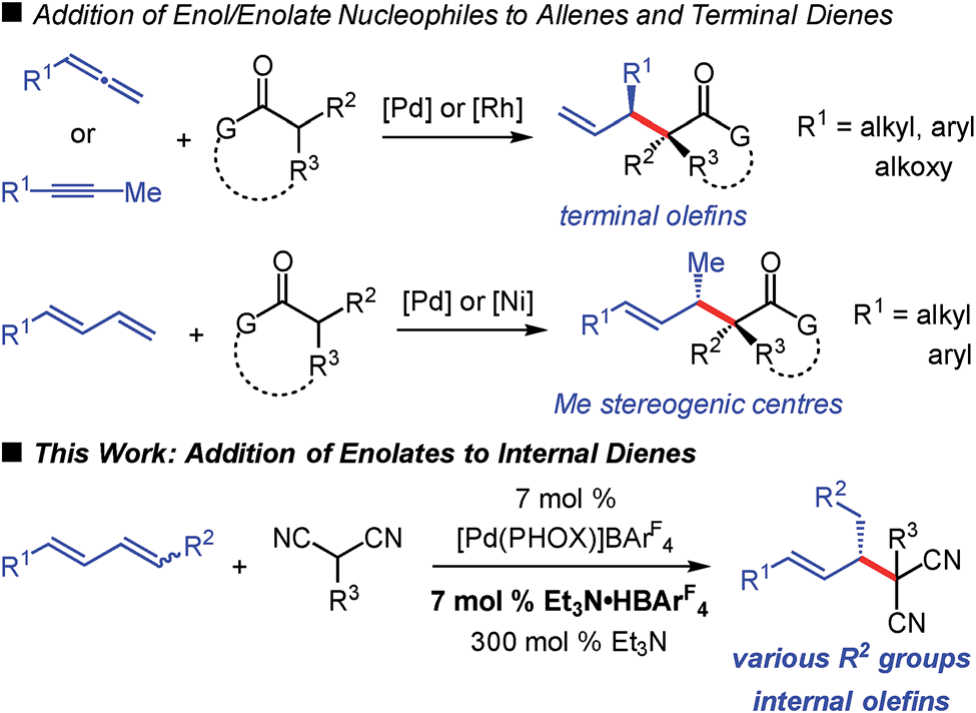
Catalytic enantioselective additions of enols/enolates to olefins.

The use of olefins as alkylating agents^7^ offers a number of distinct advantages, not the least of which is the inherent stability of an alkene compared to the lability of a leaving group, facilitating carrying the olefin electrophile through several transformations in a multistep sequence. A handful of researchers have explored this approach in enantioselective catalysis. For example, enol/enolate addition to allenes^8^ has afforded products with a variety of substituents at the β-stereogenic centre (Scheme 1), in some cases with additional control at the carbonyl's α-position.8a,c Employing allenes as electrophiles has exclusively^9^ resulted in products bearing a terminal alkene. In a similar process, Rh-bis(phosphine)-catalysed enol additions to internal alkynes delivers analogous products.^10^‡
‡Reactions of alkynes proceed through the identical metal–π-allyl intermediate as those of allenes. Terminal dienes have also emerged as alkyl electrophiles capable of affording products comprised of internal alkenes.^11^ Recently, our group described the Pd–PHOX-catalysed addition of activated pro-enolates to these dienes,^12^–^14^ and Xiao, Zhou and co-workers demonstrated diene reactions in Ni–bis(phosphine)-catalysed additions of enolates formed from simple ketones (Scheme 1).^15^ However, the products obtained from reactions of terminal dienes bear only a methyl group at the β-stereogenic centre.

In comparison, hydrofunctionalisation of internal dienes delivers molecules with both disubstituted olefins and myriad groups at the stereogenic β-position. Still, enantioselective additions to acyclic 1,4-disubstituted dienes are challenging: only indole^16^ and amine^17^ couplings have been reported. In this work, we illustrate that malononitrile and related pronucleophiles undergo highly regio- and enantioselective additions to internal dienes (Scheme 1). High reaction efficiency was achievable by the combination of an electron-deficient Pd–PHOX catalyst and Et_3_N·HBAr^F^_4_ as Brønsted acid co-catalyst.

## Results and discussion

### Method development and product functionalisation

We initiated our investigation by examining the addition of malononitrile to diene **1a** with Pd–PHOX catalyst **Pd-1**, the optimal catalyst for internal diene hydroamination,^17^ which bears the non-coordinating BAr^F^_4_ counterion (Table 1). We hypothesized that the small size of malononitrile, its low p*K*_a_,^18^ and the relatively high nucleophilicity of its anion^19^ would make it exceptionally reactive towards alkylation. Indeed, in the addition of malononitrile to 1-phenylbutadiene—a terminal diene—we found it difficult to suppress double alkylation.^12^ In the absence of any additive, there is no observable reaction with diene **1a** (entry 1); however, with 300 mol% Et_3_N, the desired **2a** is generated in >99 : 1 er as the sole product of the reaction but in only 24% yield (entry 2).

**Table 1:** Reaction optimization for diene–malononitrile coupling^*a*^

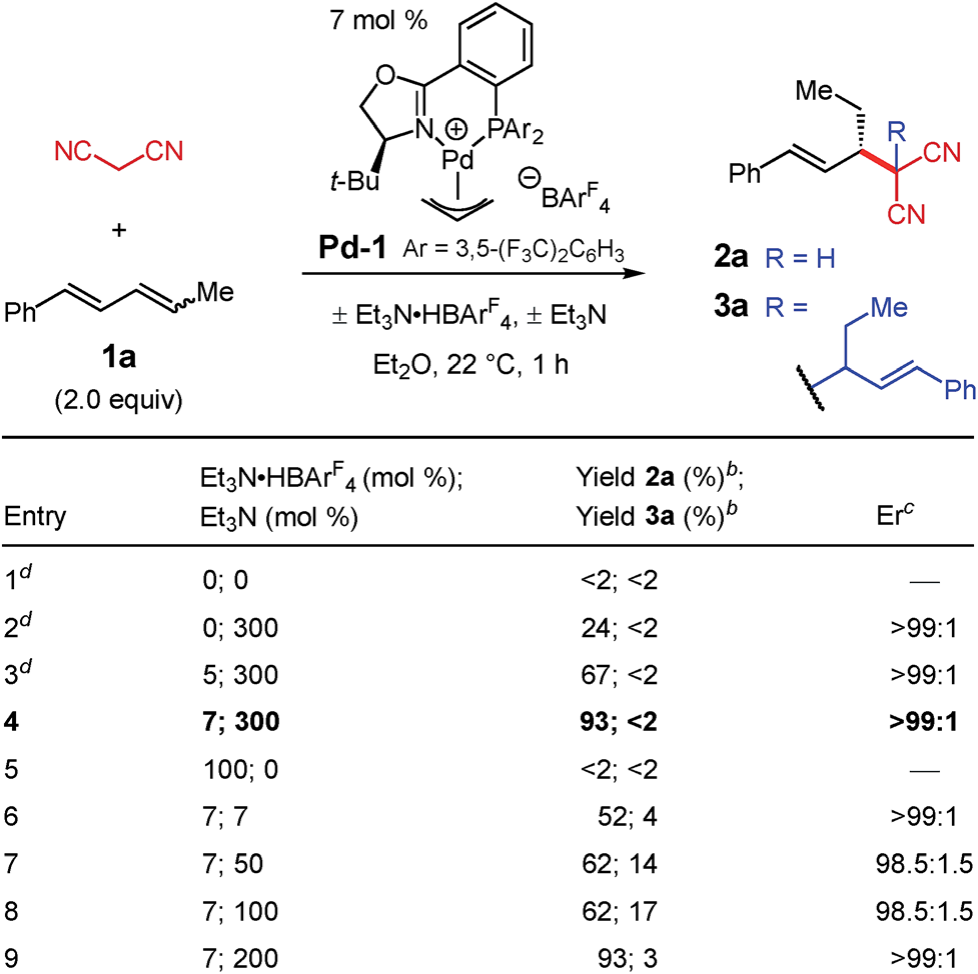

^*a*^Reaction under N_2_ with 0.2 mmol malononitrile in 0.2 mL Et_2_O. **1a** used as a 1.8 : 1 *E*,*Z* : *E*,*E* mixture of stereoisomers. See the ESI for experimental details.

^*b*^Isolated yield of purified product.

^*c*^Determined by HPLC analysis of purified **2**.

^*d*^Reaction with 5 mol% **Pd-1**.

We discovered that the addition of a substoichiometric quantity of Et_3_N·HBAr^F^_4_ as a Brønsted acid greatly improves the product yield (entry 3). Notably, the addition of Brønsted acid has been observed as a crucial component in several other transformations involving nucleophilic additions to olefins,^20^ including reactions of enols.8a–d,10a Further raising the catalyst loading of both **Pd-1** and Et_3_N·HBAr^F^_4_ to 7 mol%, which proved to be the optimal conditions, allows **2a** to be isolated in 93% yield and >99 : 1 er (entry 4). In the absence of Et_3_N, even with stoichiometric Et_3_N·HBAr^F^_4_ (entry 5), alkylation product **2a** is not formed. Lesser quantities of the base, from 7 mol% up to 100 mol%, drastically reduce the yield of **2a** while also enabling its conversion to bis-alkylation product **3a** (entries 6–8). With 200 mol% Et_3_N, product **2a** is again isolated in 93% yield but is still accompanied by a small quantity of byproduct **3a** (entry 9).

Under the optimal conditions, several 1,4-disubstituted dienes couple with malononitrile (Table 2). Electronic perturbations of the diene's aryl group are tolerated, affording **2b–d** in 40–66% yield and >95 : 5 er. Moving the aryl group substituent to the *meta* or *ortho* positions generates tolyl products **2e** and **2f** in higher yields (87–89%) and with excellent enantioselectivity (97.5 : 2.5 and 96 : 4 er, respectively). Changing the substituent at the 4-position of the diene enables products with a variety of alkyl groups at the stereogenic carbon to be obtained (**2g–k**); the reaction is tolerant of both ethereal (**2i–j**) and carbonyl functionality (**2h** and **2k**). All aryl/alkyl-substituted diene substrates deliver malononitrile products **2a–k** as a single regioisomer. Additionally, 1,4-dialkyl-containing dienes also afford malononitrile addition products as a single regioisomer as long as the two alkyl groups are sterically differentiated. Vinylcyclohexane **2l** is obtained in 72% yield and 94 : 6 er. In all cases, enantioselectivity is constant throughout the course of the reaction, indicating that the process is irreversible.

**Table 2:** Internal diene scope for malononitrile additions^*a*^

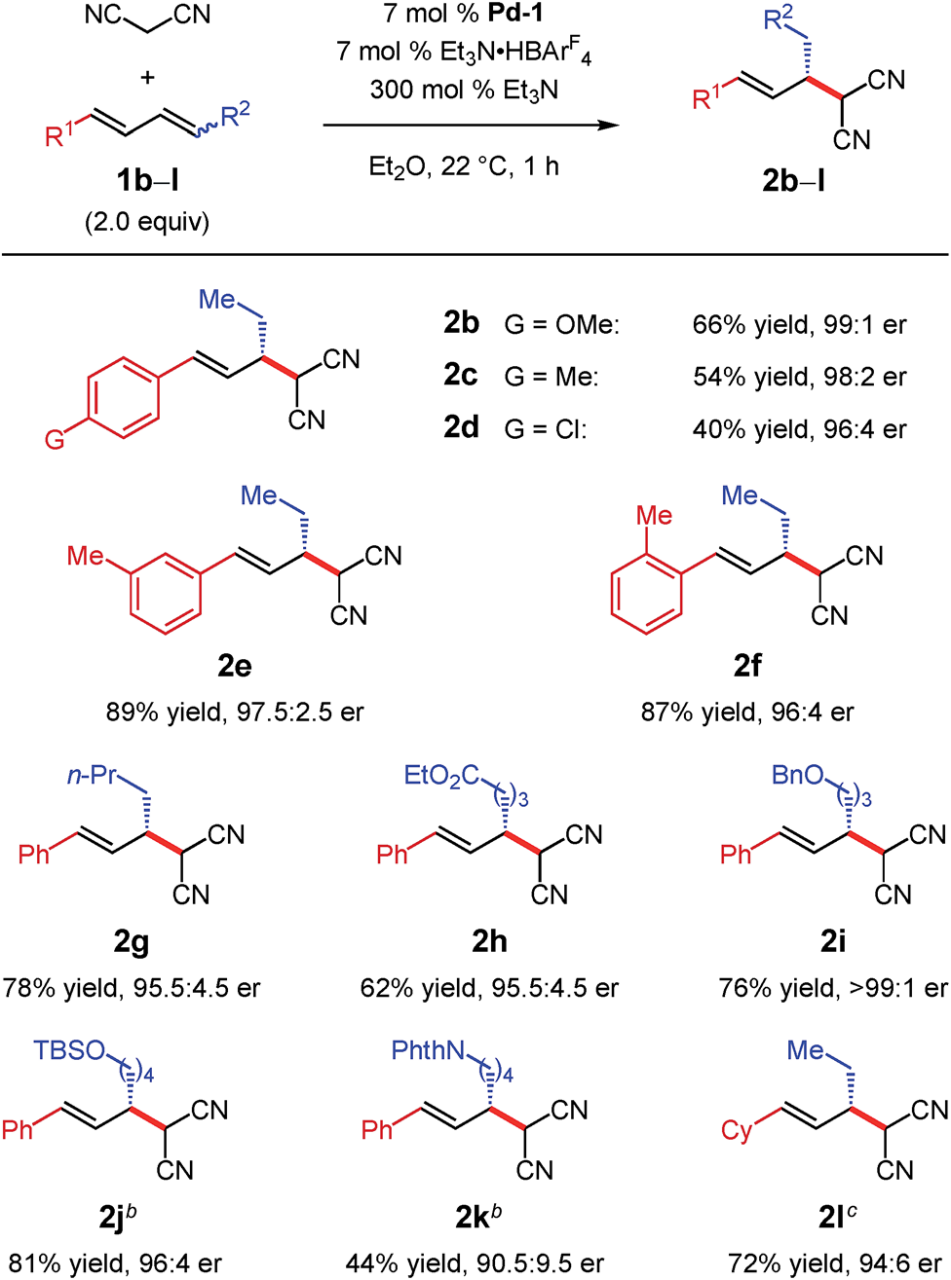

^*a*^Reaction under N_2_ with 0.2 mmol malononitrile in 0.2 mL Et_2_O. Dienes were used as a mixture of stereoisomers (1.1 : 1 to 4.2 : 1 *E*,*Z* : *E*,*E*). See the ESI for experimental details.

^*b*^Isolated yield of purified product.

^*c*^Determined by HPLC analysis of purified **2**.

Substituted malononitrile pronucleophiles undergo **Pd-1**-catalysed addition to diene **1a** within 1 h with complete regiocontrol (Table 3). The methyl-, benzyl-, and cinnamyl-substituted pronucleophiles generate products **5a–c** in 78–99% yield and >95 : 5 er. Acetylacetone, both less acidic^21^ and its anion less nucleophilic^19^ than malononitrile, leads to diketone **5d** in 51% yield and 92.5 : 7.5 er after 20 h, still as a single regioisomer. Ethyl cyanoacetate addition to diene **1a**, however, results in a 60 : 40 mixture of product regioisomers **5e** and **6e** (56% yield). Isomer **6e** arises from addition of the nucleophile to the phenyl-substituted carbon of the π-allyl–**Pd-1** intermediate derived from diene **1a** whereas **5e** results from nucleophile addition to the ethyl-substituted carbon of the same π-allyl complex. Dimethyl malonate, delivers a similar result (57 : 43 **5f** : **6f** in 49% yield). Despite their lower p*K*_a_'s^21^,^22^ but perhaps partially due to their lower nucleophilicities,^19^ cyclic pronucleophiles such as Meldrum's acid and dimedone fail to undergo addition to diene **1a** under the conditions shown in Table 3.

**Table 3:** Pronucleophile scope for internal diene addition reactions^*a*^ ^,^^*b*^ ^,^^*c*^

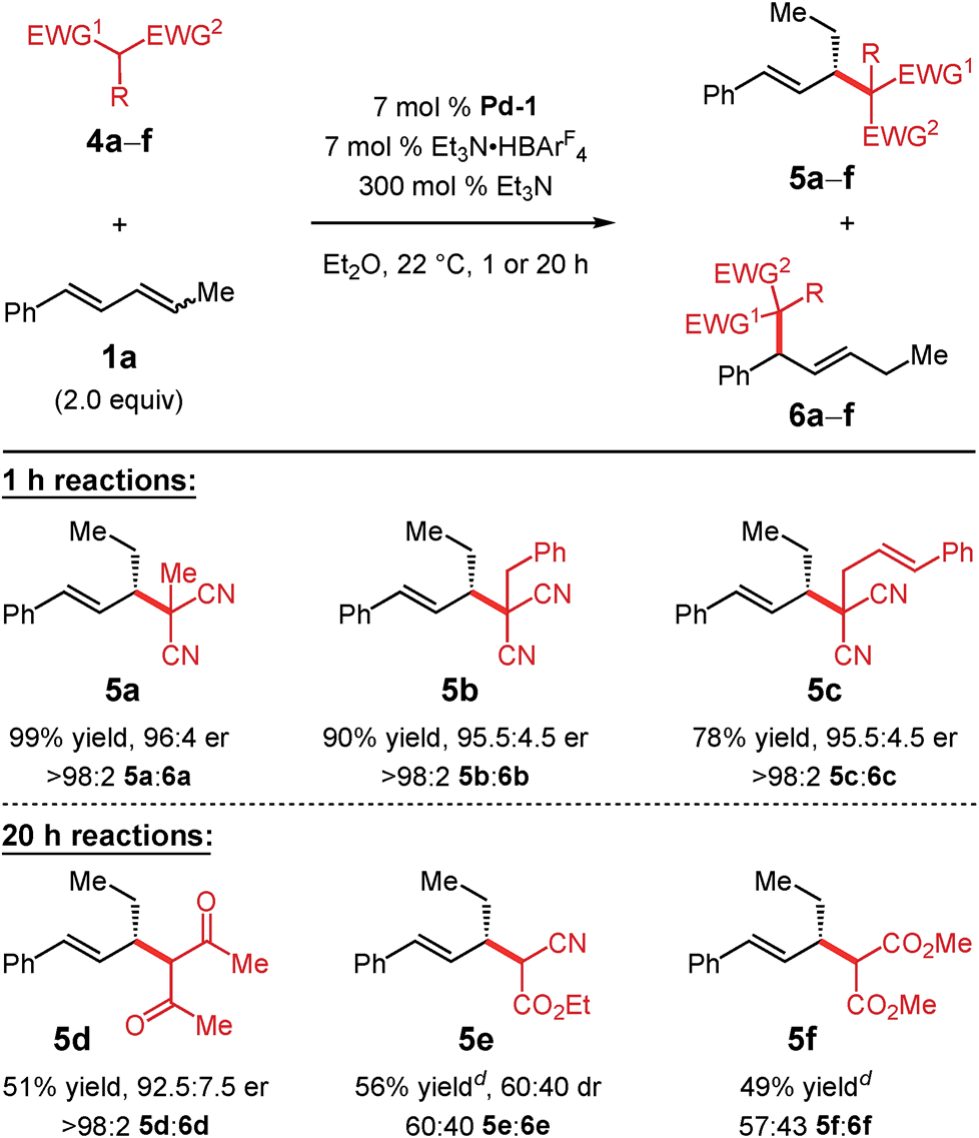

^*a*^Reaction under N_2_ with 0.2 mmol pronucleophile in 0.2 mL Et_2_O. **1a** used as a 1.8:1 *E,Z : E,E* mixture of stereoisomers.

^*b*^Isolated yield of purified product.

^*c*^er determined by HPLC analysis of purified **5**.

^*d*^Isolated yield of the isomeric mixture; er not determined.

**Scheme 2:**
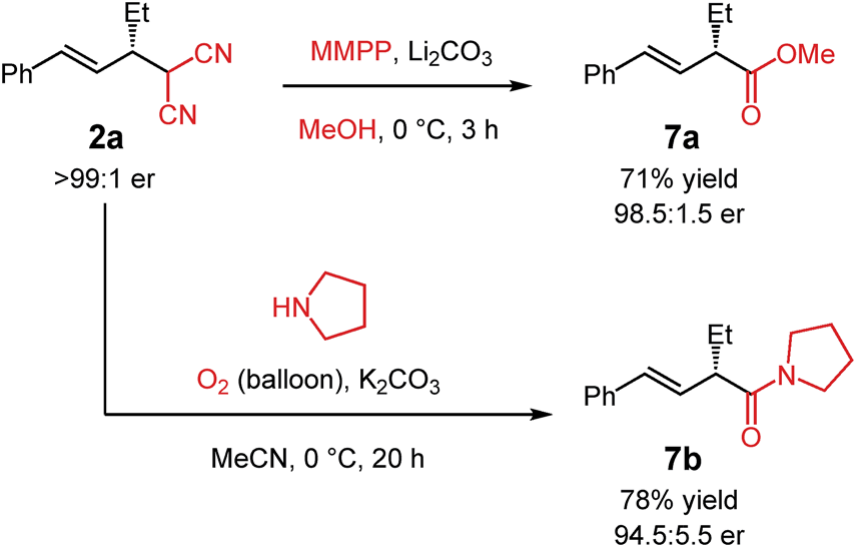
Synthesis of α-alkenyl carbonyls from the malononitrile group.

The malononitrile adducts of internal dienes are useful building blocks for further synthesis. For example, the malononitrile unit within **2a** (Scheme 2) may undergo oxidative methanolysis^23^*via* the cyanoketone to deliver α-alkenylester **7a** in 71% yield with minimal erosion of enantiopurity. Similarly, oxidation in the presence of pyrrolidine leads to α-alkenylamide **7b** in 78% yield (94.5 : 5.5 er).^24^

### Mechanism studies

A mechanism for Pd–PHOX-catalysed addition of malononitrile to internal dienes is illustrated in Scheme 2.^17^,^25^ After initiation of **Pd-1** by nucleophilic attack of the malononitrile anion upon its π-allyl ligand, coordination of diene **1a** to the Pd(0) species leads to intermediate **i**. Oxidative protonation at Pd by Et_3_N·HBAr^F^_4_ leads to hydrido complex **ii**, which undergoes migratory insertion, ultimately forming π-allyl–Pd species **iii**. The equilibration is accelerated and shifted towards complex **iii** by the addition of exogenous Et_3_N·HBAr^F^_4_ as the co-catalyst. At the same time, deprotonation of malononitrile by Et_3_N leads to the malononitrile anion **iv**, an equilibrium that favours the neutral molecule. The anion may then attack π-allyl–Pd **iii** through an outer sphere pathway. The C–C bond-forming event to form **v** appears to be irreversible and simultaneously regenerates Et_3_N·HBAr^F^_4_. Exchange of the olefin within **v** for diene **1a** releases product **2a** and regenerates complex **i**.

**Scheme 3:**
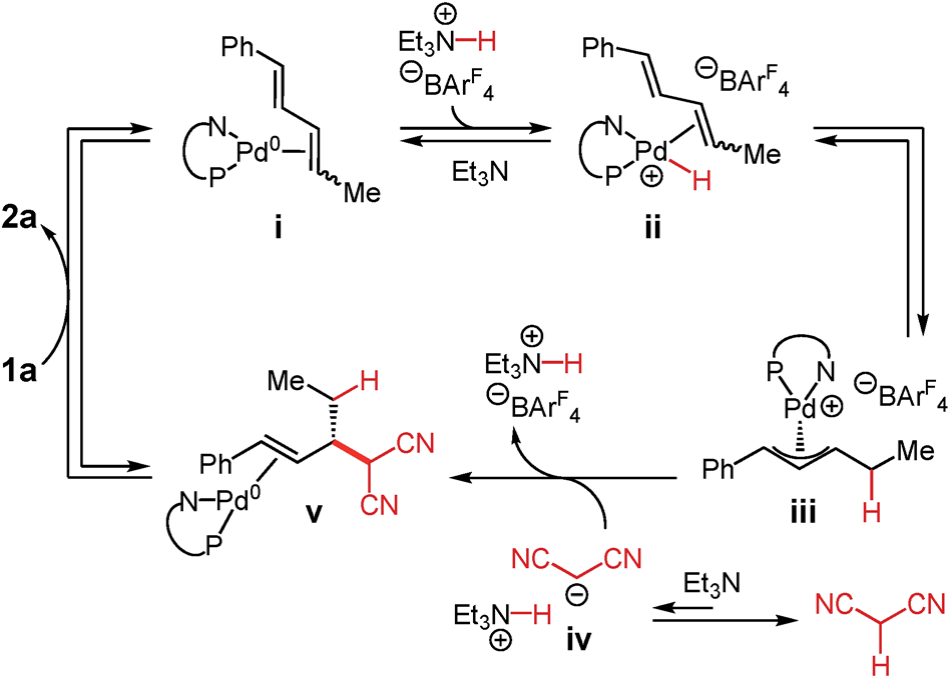
Proposed mechanism of malononitrile addition to internal dienes.

In the context of this mechanistic proposal, we sought to address several facets of the Pd–PHOX-catalysed diene–enolate couplings. These investigations might allow us to glean further information about individual steps of this mechanism and more broadly understand diene couplings to nucleophiles promoted by Pd–PHOX catalysts.

**Scheme 4:**
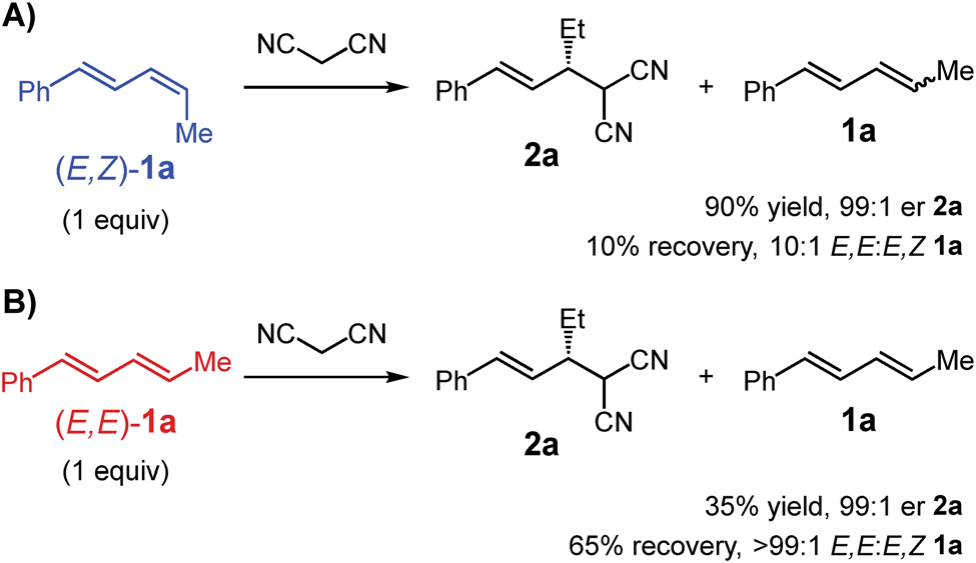
Comparison of reactivity of diene diastereomers; conditions: **Pd-1** (7 mol%), Et_3_N·HBAr^F^_4_ (7 mol%), Et_3_N (300 mol%), Et_2_O, 22 °C, 1 h.

We first set out to investigate the impact of diene stereochemistry upon the reaction. The transformations presented in Tables 1–3 utilize an *E*,*Z/E*,*E*-mixture of diene diastereomers favouring the *Z*-stereoisomer at the alkyl-substituted alkene. As shown in Scheme 4A, *E*,*Z*-**1a** is significantly more reactive than the *E*,*E*-stereoisomer, delivering malononitrile **2a** in 90% yield and 99 : 1 er. Comparatively, *E*,*E*-**1a** (Scheme 4B) leads to only 35% yield of **2a** (99 : 1 er). In both experiments, one equivalent of diene was employed and any left unreacted was completely recovered. From the reaction of *E*,*Z*-**1a**, the recovered diene had almost completely isomerised to the *E*,*E*-diastereomer (Scheme 4A). In contrast, the recovered diene from reaction of *E*,*E*-**1a** was still entirely that stereochemistry (Scheme 4B).

These data also help explain why under the optimal conditions, product **2a** does not undergo a second alkylation event despite the presence of excess diene **1a** (see Table 1). Of the two equivalents of diene **1a** added to the reaction, only *ca.* 65% (1.3 equiv.) is the more reactive *E*,*Z*-stereoisomer. The data also suggest the catalyst is capable of reacting with the diene and isomerising the *E*,*Z*- to the *E*,*E*-diastereomer without product formation. Therefore, during the course of the reaction, as mono-alkylated product builds up, there is concomitant isomerisation of the diene occurring (likely along with catalyst decomposition), thereby slowing formation of bis-alkylated **3a**. With fewer Et_3_N equivalents, **3a** is observed to some degree (Table 1). This might be attributable to the equilibrium (see Scheme 3) between malononitrile and its deprotonated form **iv** being shifted towards the neutral molecule when less base is available, slowing the rate of formation of **2a**. Mono-alkylated **2a** that is formed may then engage in subsequent reaction with remaining *E*,*Z*-**1a** prior to its consumption in product formation or its stereoisomerisation. Additionally, although substituted malononitriles, such as those shown in Table 3, are competent reaction partners, compound **2a** is significantly more hindered (β-branching) which slows a second alkylation event.

**Scheme 5:**
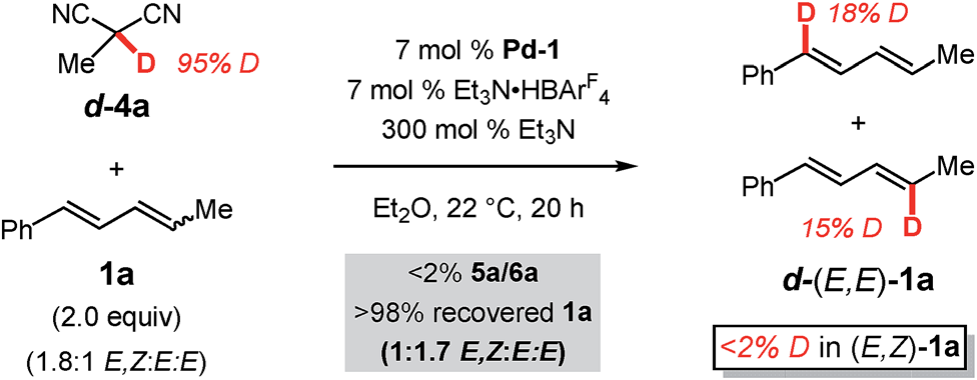
Deuterium labelling studies with deuterated methylmalononitrile

We next further sought to assess the reversibility of the individual elementary steps connecting diene **1a** to π-allyl–Pd **iii** (Scheme 3). To do so, we examined the coupling of a deuterated pronucleophile, (1) to discover the label's position in the anticipated addition product with respect to the site of C–C bond formation and (2) to determine whether deuterium could be incorporated into the diene without product forming. We chose deuterated 2-methylmalononitrile (***d*-4a**) as the pronucleophile (Scheme 5). Incredibly, although the protic version of **4a** delivers **5a** in 99% yield after 1 h, no coupled product is formed after 20 h with ***d*-4a**. This is likely due to the combination of a significant kinetic isotope effect and catalyst decomposition. Diene **1a** could be completely recovered from the mixture: a portion of the diene had isomerised from the *E*,*Z*-isomer to (*E*,*E*)-**1a** (from 1.8 : 1 to 1 : 1.7 *E*,*Z* : *E*,*E*). A significant percentage of the deuterium label (*ca.* 43%) was transferred from ***d*-4a** to the diene with the label confined solely to the recovered *E*,*E*-**1a** and roughly evenly distributed between C1 and C4.

Just as in hydroamination of **1a** with **Pd-1** (reaction of N-deuterated indoline),^17^ the kinetic isotope effect observed here is likely at least partially due to an equilibrium isotope effect that exchanges deuterium from ***d*-4a** to diene **1a** as mediated by Et_3_N and the Pd catalyst. The lack of label in recovered (*E*,*Z*)-**1a** is owed to its conversion exclusively to ***d***-(*E*,*E*)-**1a** when it reacts with Pd–D: stereochemical isomerisation of the alkene occurs by Pd–D migratory insertion to (*E*,*Z*)-**1a**, bond rotation, and preferential β-hydride elimination (compared to β-deuteride elimination). As mentioned with regard to the reactivity difference of the two diene stereoisomers (*vide supra*), this process does not occur to incorporate deuterium into (*E*,*Z*)-**1a***via* reaction of (*E*,*E*)-**1a**. Finally, the distribution of the label in ***d***-(*E*,*E*)-**1a** indicates that palladium–hydride/deuteride insertion may occur at either olefin of the diene, yet only insertion at the alkyl-substituted olefin leads to product in the reactions presented in Tables 1–3. The results further confirm that *E*,*Z*-diene coordination to Pd is reversible.

**Scheme 6:**
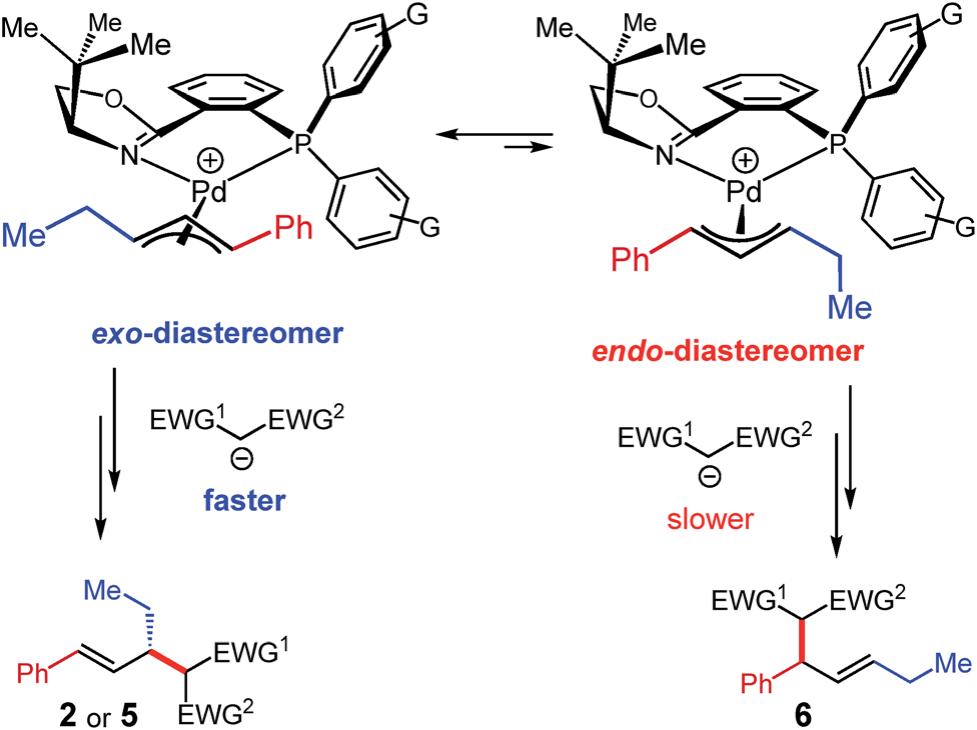
Curtin–Hammett kinetics likely operative in internal diene–enolate couplings and responsible for the observed regio- and enantioselectivity.

It is noteworthy that whereas malononitrile and acetylacetone nucleophiles afford a single detectable product regioisomer (Tables 1–3), ethyl cyanoacetate and dimethylmalonate deliver a mixture (**5e**/**6e** and **5f**/**6f**, respectively, Table 3). As mentioned earlier, the product isomers arise from attack of the nucleophiles upon two different carbons of the same π-allyl. Differing regioselectivities among nucleophiles is likely due to Curtin–Hammett kinetics in the reaction (Scheme 6) and is the combination of three properties of the nucleophile: (1) the p*K*_a_ of the pronucleophile (affecting its equilibrium with the active deprotonated nucleophile), (2) the nucleophilicity of the carbanion,^19^ and (3) the sterics of the nucleophile. As documented previously for Pd–PHOX complexes,3*a* the π-allyl–Pd species exist as a mixture of the thermodynamically preferred *exo*-diastereomer and its isomeric *endo*-complex (Scheme 6). In this transformation, faster attack of the nucleophile upon the *exo*-isomer at the carbon *trans* to the phosphine (ethyl-substituted carbon) leads to the observed major enantiomer and regioisomer (**2 **or **5**). Conversely, some nucleophiles undergo competitive addition to the *endo*-diastereomer, attacking the phenyl-substituted carbon (*trans* to the phosphine), affording **6**. Although ethyl cyanoacetate has a similar p*K*_a_ (ref. 26) to acetylacetone, generating a certain quantity of active nucleophile, its nucleophilicity is considerably greater (similar to malononitrile^19^). Likewise, even though dimethyl malonate has an even higher p*K*_a_,^22^ its nucleophilicity is greater still.^19^ Therefore, it is possible that reactants with greater nucleophilicity lead to similar rates of attack upon the *exo*- and *endo*-diastereomers of the π-allyl intermediate. The outlier is malononitrile, whose lower p*K*_a_ (ref. 18) and high nucleophilicity^19^ should seemingly lead to an isomeric mixture. The high selectivity for products **2** or **5** with malononitrile (*via* the *exo*-isomer) is likely due to the small size of this pronucleophile.

An interesting dichotomy between reactions of terminal dienes, such as 1-phenylbutadiene, and those of internal dienes with activated C-pronucleophiles as catalysed by Pd–PHOX complexes presented itself in this study. In our previous investigations of C-pronucleophile additions to terminal dienes,^12^ Meldrum's acid proved to be one of the best and most versatile pronucleophiles. Those reactions are promoted by the same PHOX ligand for Pd as in **Pd-1** but with a catalyst that bears a tetrafluoroborate counterion in place of BAr^F^_4_. Additionally, terminal diene reactions do not require Brønsted acid additive. Contrastingly, dimethyl malonate fails to undergo addition to 1-phenylbutadiene under the previously established optimal terminal diene conditions with the BF_4_-containing catalyst.

**Scheme 7:**
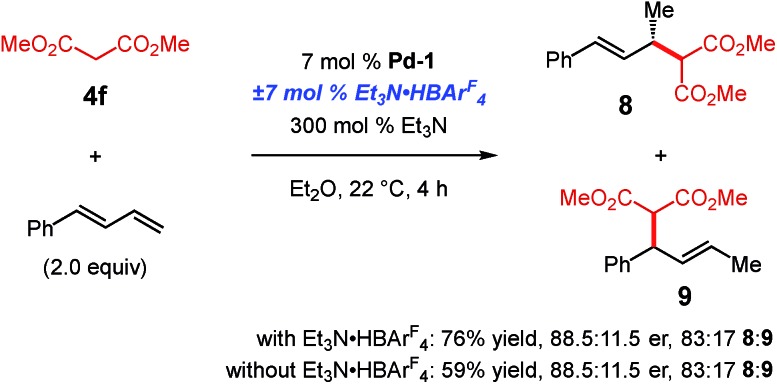
Addition of dimethyl malonate to phenylbutadiene with **Pd-1**.

We wished to determine whether these differences arise from the BAr^F^_4_ counterion of **Pd-1** or the Et_3_N·HBAr^F^_4_ additive. As shown in Scheme 7, dimethyl malonate **4f** is able to add to 1-phenylbutadiene in the presence of **Pd-1** (BAr^F^_4_ counterion). Although the yield is slightly higher with the addition of Et_3_N·HBAr^F^_4_, both the regioselectivity and enantiopurity of the major product (**8**) are the same with or without Brønsted acid. Therefore, higher p*K*_a_ pronucleophiles may react with terminal dienes with a Pd–PHOX catalyst comprised of the non-coordinating BAr^F^_4_. It is noteworthy that the regiomeric ratio favouring the 4,3-addition product **9** is higher for dimethyl malonate coupling to phenylbutadiene than to internal diene **1a** (*cf.*, Table 3, compound **5f**).

We finally turned to addressing the lack of reactivity of Meldrum's acid towards internal diene **1a** with **Pd-1**. In fact, not only does C–C bond formation fail to occur in this case, based on the diene recovered from the reaction mixture, the initial 1.8 : 1 *E*,*Z* : *E*,*E* ratio of **1a** also remains unchanged. The absence of stereochemical isomerisation indicates that catalyst formed from **Pd-1** fails to engage the diene at all in the presence of this pronucleophile.

**Scheme 8:**
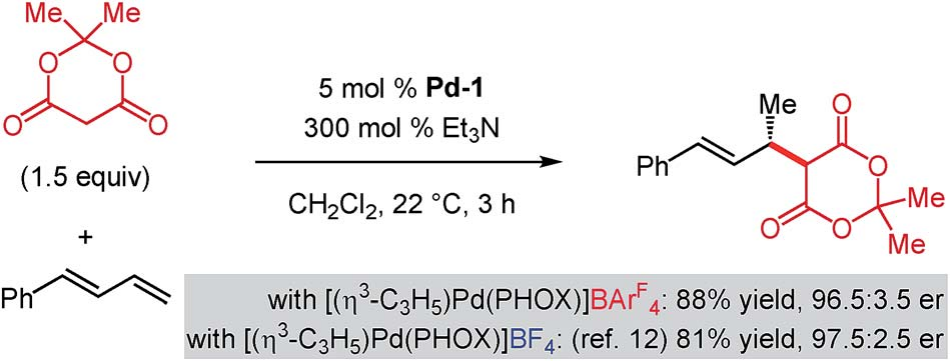
Meldrum's acid addition to phenylbutadiene with Pd–PHOX catalysts bearing different counteranions.

As mentioned, we previously developed the addition of Meldrum's acid to 1-phenylbutadiene with [(η^3^-C_3_H_5_)Pd(PHOX)]BF_4_ as the catalyst.^12^ Unfortunately, reactions of internal dienes are completely inhibited by having a BF_4_ counterion in the reaction medium, preventing our examining Meldrum's acid reaction with internal diene **1a** catalysed by [(η^3^-C_3_H_5_)Pd(PHOX)]BF_4_. However, Meldrum's acid addition to 1-phenylbutadiene occurs smoothly with BAr^F^_4_-containing **Pd-1**, delivering a nearly identical result to its BF_4_ analogue (Scheme 8). These data highlight that the acidic Meldrum's acid is compatible with **Pd-1** no matter what its counteranion as long as a more reactive terminal diene is present. This suggests that as the internal diene **1a** is more difficult to coordinate to the metal centre, significant catalyst decomposition occurs under the more acidic conditions imposed with Meldrum's acid (lower p*K*_a_) compared to other pronucleophiles, thereby impeding the desired reaction pathway.

## Conclusions

We have demonstrated that internal dienes act effectively as alkylating agents for enantioselective intermolecular couplings with *in situ*-generated enolates under Pd–PHOX catalysis. The atom economic olefin hydrofunctionalisations are accelerated by the addition of Et_3_N·HBAr^F^_4_ as a Brønsted acid co-catalyst. Somewhat counterintuitively, the use of superstoichiometric Et_3_N is needed to generate sufficient quantities of the active nucleophile in order to form the mono-alkylation product selectively (*i.e.*, avoid over-alkylation). Several features of the reaction mechanism have been uncovered through deuterium labelling the pronucleophile and by selectively employing different diastereomerically pure diene stereoisomers in reactions. Notable differences in participating C-pronucleophiles in Pd–PHOX-catalysed reactions of internal *versus* terminal 1,3-dienes have been unveiled. Further investigation of reaction mechanism and applications to additional methodology development from lessons learned in these studies are ongoing.

## Conflicts of interest

There are no conflicts to declare.

## Supplementary Material

Supplementary information
