# Supplementary material for: Brønsted acid and Pd–PHOX dual-catalysed enantioselective addition of activated C-pronucleophiles to internal dienes
Source: Chem Sci. 2019 Apr 17;10(19):5176–82. doi: 10.1039/c9sc00633h (PMC6524568; doi:10.1039/c9sc00633h)
Supplement: Supplementary file 1 [file SC-010-C9SC00633H-s001.pdf]

Electronic Supplementary Material (ESI) for Chemical Science.
This journal is © The Royal Society of Chemistry 2019

Supporting Information

Brønsted Acid and PHOX–Pd Dual-Catalyzed Enantioselective Addition of Activated *C*-Pronucleophiles to Internal Dienes

Sangjune Park and Steven J. Malcolmson*

Department of Chemistry, Duke University
Durham, North Carolina 27708 USA

E-mail: steven.malcolmson@duke.edu

Table of Contents

I. General Information S2

II. Preparation of Preparation of Catalyst, Ligands and Substrates S4

III. Supplemental Screening Data S6

IV. Substrate Scope S9

V. Additonal Experiments S29

VI. Product Derivatizations S30

VII. Reaction Outcome Dependence on Diene Stereochemistry S32

VIII. Deuterium Labelling Studies S33

IX. References S34

X. NMR Spectra S35

I. General Information

General Procedures. All reactions were carried out in oven- (120 °C) or flame-dried glassware under an inert atmosphere of dry N_2 unless otherwise noted. Oven-dried (60 °C or 120 °C) stainless steel cannulas and/or glass syringes (or N_2-flushed plastic syringes) were used for reagent transfer. Organic solutions were concentrated under reduced pressure using a rotary evaporator (Büchi). Flash column chromatography was performed using SiliCycle SiliaFlash® P60 Silica Gel.

Reagents.

trans-1-Bromo-1-propene (Sigma-Aldrich), *β*-bromostyrene (Sigma-Aldrich), 1,3-bis(trifluoromethyl)-5-bromobenzene (Chem-Impex), *n*-BuLi in cyclohexane 2.0 M (Sigma-Aldrich), (*n*-butyl)triphenylphosphonium bromide (Acros), carbon tetrabromide (TCI), catecholborane (Sigma-Aldrich), chlorobis[3,5-bis(trifluoromethyl)phenyl]phosphine (Alfa Aesar), chlorobis[4-(trifluoromethyl)phenyl]phosphine (Alfa Aesar), cesium carbonate (Sigma-Aldrich), 4-chlorocinnamaldehyde (Sigma-Aldrich), 4-chlorocinnamic acid (Matrix), *trans*-cinnamaldehyde (Alfa Aesar), diisobutylaluminum hydride (Sigma-Aldrich), ethylenediamine (Sigma-Aldrich), ethyltriphenylphosphonium bromide (Acros), lithium carbonate (Sigma-Aldrich), magnesium monoperoxyphthalate (Sigma-Aldrich), 4-methylcinnamic acid (Sigma-Aldrich), *trans-p*-methoxycinnamaldehyde (Acros), potassium tert-butoxide (Strem), potassium phthalimide (Sigma-Aldrich), 1-propynyl magnesium bromide in THF 0.5 M (Sigma-Aldrich), potassium carbonate (Alfa Aesar), sodium borohydride (VWR), *o*-tolualdehyde (TCI) and triphenylphosphine (Sigma-Aldrich) were used as received. Sodium tetrakis[3,5-bis(trifluoromethyl)phenyl]borate ($NaBAr^F_4$) was prepared according to a reported procedure.[1]

Solvents. Solvents were sparged with dry N_2 and purified under a positive pressure of dry N_2 by an Innovative Technologies PureSolve solvent purification system: tetrahydrofuran (Sigma-Aldrich), dichloromethane (Sigma-Aldrich) and diethyl ether (Sigma-Aldrich), and toluene (Sigma-Aldrich) were passed through two consecutive alumina columns. Acetonitrile (Fisher) and ethanol (200 proof, Koptec) used for reactions were distilled over CaH_2 prior to use. Benzene (*anhyd.*, EMD Millipore), methanol (Sigma-Aldrich), DMF (*anhyd.*, Alfa Aesar), and *tert*-butanol were used as received. Hexanes (Fisher) and ethyl acetate (Fisher) were used for flash column chromatography and used as

received. HPLC-grade hexanes (Sigma-Aldrich), methanol (Sigma-Aldrich), acetonitrile (Sigma-Aldrich) and isopropanol (Sigma-Aldrich) were used as received.

Instrumentation. ^{1}H NMR spectra were recorded on a Varian INOVA (400 MHz) and Bruker Advance Neo (500 MHz) spectrometer. Chemical shifts are reported in ppm from tetramethylsilane with the solvent resonance resulting from incomplete deuteration as the internal reference ($CDCl_3$: *δ* 7.24). Data are reported as follows: chemical shift, integration, multiplicity (s = singlet, d = doublet, t = triplet, q = quartet, br = broad, m = multiplet, app. = apparent), coupling constant(s) (Hz). ^{13}C NMR spectra were recorded on a Varian/Agilent VNMRS (500 MHz) or Bruker Advance Neo (500 MHz) spectrometer with complete proton decoupling. Chemical shifts are reported in ppm from tetramethylsilane with the signal from chloroform as the internal reference ($CDCl_3$: *δ* 77.08). ^{19}F NMR spectra were recorded on a Varian INOVA (400 MHz) or Bruker Advance Neo (500 MHz) spectrometer. Enantiomer ratios (er) were determined by HPLC (Phenomenex™ Lux® Cellulose I, III or Phenomenex™ Lux® Amylose I, II) in comparison with authentic racemic materials on a Shimadzu Prominence Modular HPLC. High-resolution mass spectrometry was performed on an Agilent (1200 Series) LCMS-TOF-DART at the Duke University Mass Spectrometry Facility. MALDI-MS data were recorded on a Bruker Autoflex Speed LRF MALDI-TOF. Specific rotation values were recorded on a Rudolph Autopol IV Polarimeter. Infrared (IR) spectra were collected on a Nicolet 6700 FT-IR spectrometer, υ_{max} in cm^{-1}. Bands are characterized as broad (br), strong (s), medium (m), or weak (w). Melting points were measured on an Electrothermal MelTemp® capillary melting point apparatus and are uncorrected.

HRMS data are an average of 3–4 runs. Polarimetry data are an average of 5–6 trials. Melting point data are an average of 3 runs.

II. Preparation of Catalyst, Ligands and Substrates

1. Preparation of Catalyst and Ligands: Pd-1 complex and ligands (**L2** and **L3**) were prepared as previously described.[2,3]

2. Preparation of Internal Diene Substrates

The following internal diene substrates were prepared as previously described:[2] ((1*E*)-Penta-1,3-dien-1-yl)benzene (**1a**), 1-methoxy-4-((1*E*,3*E*)-penta-1,3-dien-1-yl)benzene (**1b**), 1-methyl-4-((1*E*)-penta-1,3-dien-1-yl)benzene (**1c**), 1-chloro-4-((1*E*)-penta-1,3-dien-1-yl)benzene (**1d**), 1-methyl-3-((1*E*)-penta-1,3-dien-1-yl)benzene (**1e**), 1-methyl-2-((1*E*)-penta-1,3-dien-1-yl)benzene (**1f**), ((1*E*)-hepta-1,3-dien-1-yl)benzene (**1g**), ethyl (6*E*)-7-phenylhepta-4,6-dienoate (**1h**), ((1*E*)-6-(benzyloxy)hexa-1,3-dien-1-yl)benzene (**1i**), *tert*-butyldimethyl(((6*E*)-7-phenylhepta-4,6-dien-1-yl)oxy)silane (**1j**), ((1*E*)-penta-1,3-dien-1-yl)cyclohexane (**1l**) and (6*E*)-7-Phenylhepta-4,6-dien-1-ol (**A**).

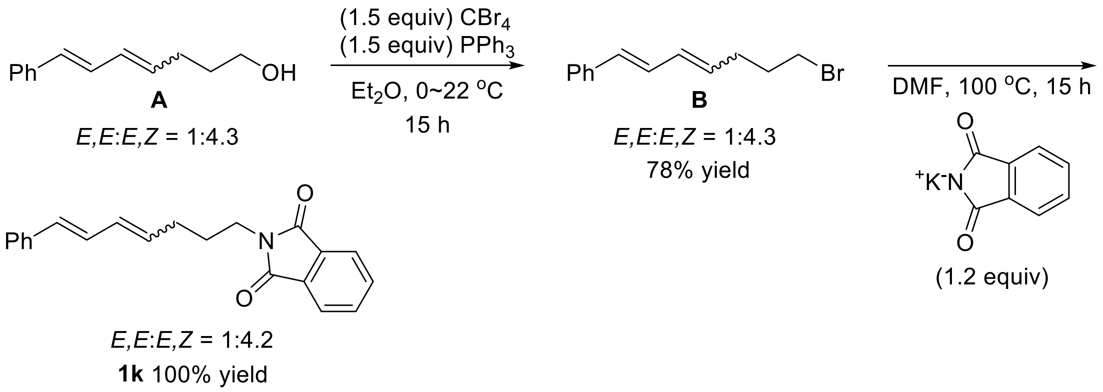

((1*E*)-7-Bromohepta-1,3-dien-1-yl)benzene (B): To a dry 100-mL round-bottom flask equipped with a magnetic stirring rod was added carbon tetrabromide (4.97 g, 15.0 mmol, 1.50 equiv), triphenylphosphine (3.93 g, 15.0 mmol, 1.50 equiv) and Et_2O (25 mL). The mixture was allowed to stir at 0 °C. After 15 min, **A** (1.88 g, 10.0 mmol, 1.00 equiv) was added and the reaction mixture was then allowed to stir and warm to ambient temperature. After 15 h, the mixture was poured into a separatory funnel filled with water (100 mL). The organics were extracted with EtOAc (3 X 50 mL). The combined organic fractions were dried over $MgSO_4$, filtered, and concentrated. The residue was purified by silica gel column chromatography (100% hexanes to 90:10 hexanes:EtOAc) to give **B** (2.15 g, 8.56 mmol, 85.6% yield) as a colorless oil. ***E,Z*-Major isomer**: **^{1}H NMR** (400 MHz, $CDCl_3$) δ

7.41 (2H, d, *J* = 7.9 Hz), 7.31 (2H, t, *J* = 7.5 Hz), 7.21 (1H, t, *J* = 7.7 Hz), 7.08 (1H, dd, *J* = 15.5, 11.2 Hz), 6.54 (1H, d, *J* = 15.6 Hz), 6.21 (1H, t, *J* = 10.8 Hz), 5.50–5.41 (1H, m), 2.45 (2H, q, *J* = 7.4 Hz), 2.02–1.94 (2H, m); ***E*,*E*-Minor isomer**: **^{1}H NMR** (400 MHz, $CDCl_3$) δ 7.36 (2H, d, *J* = 8.0 Hz), 7.35–7.16 (1H, m), 6.73 (1H, dd, *J* = 15.7, 10.6 Hz), 6.46 (1H, d, *J* = 15.6 Hz), 6.28–6.19 (1H, m), 5.75 (1H, dt, *J* = 15.0, 7.3 Hz), 3.46–3.40 (2H, m), 2.30 (2H, q, *J* = 7.2 Hz), 2.02–1.94 (2H, m). Spectral data matched those previously reported.[4]

2-((6*E*)-7-Phenylhepta-4,6-dien-1-yl)isoindoline-1,3-dione (1k): To a dry 100-mL round-bottom flask equipped with a magnetic stirring rod was added **B** (1.00 g, 3.98 mmol, 1.00 equiv), potassium phthalimide (885 mg, 4.78 mmol, 1.20 equiv) and DMF (60 mL). The mixture was allowed to stir and then heated to 100 °C for 15 h. The reaction mixture was then allowed to cool to ambient temperature and poured into a separatory funnel filled with water (100 mL). The organics were extracted with EtOAc (3 X 50 mL). The combined organic fractions were dried over $MgSO_4$, filtered, and concentrated. The residue was purified by silica gel column chromatography (100% hexanes to 80:20 hexanes:EtOAc) to give **1k** (1.27 g, 3.98 mmol, >98% yield) as a white solid. ***E*,*Z*-Major isomer**: **^{1}H NMR** (500 MHz, $CDCl_3$) δ 7.81 (2H, dd, *J* = 5.4, 3.0 Hz), 7.67 (2H, dd, *J* = 5.5, 3.0 Hz), 7.38 (2H, d, *J* = 7.3 Hz), 7.35–7.14 (3H, m), 7.00 (1H, dd, *J* = 15.5, 11.2 Hz), 6.49 (1H, d, *J* = 15.5 Hz), 6.15 (1H, t, *J* = 11.0 Hz), 5.50 (1H, dt, *J* = 10.5, 7.6 Hz), 3.72 (2H, t, *J* = 7.2 Hz), 2.35 (2H, q, *J* = 7.5 Hz), 1.86–1.80 (2H, m); **^{13}C NMR** (125 MHz, $CDCl_3$) δ 168.4, 134.5, 133.9, 132.7, 132.2, 131.2, 130.0, 128.6, 127.5, 126.4, 124.1, 123.2, 37.7, 28.4, 25.4; ***E*,*E*-Minor isomer**: **^{1}H NMR** (500 MHz, $CDCl_3$) δ 7.82-7.79 (2H, m), 7.70–7.65 (2H, m), 7.35–7.14 (4H, m), 6.67 (1H, dd, *J* = 15.6, 10.4 Hz), 6.40 (1H, d, *J* = 15.7 Hz), 6.24–6.12 (1H, m), 5.78 (1H, dd, *J* = 14.7, 7.0 Hz), 3.74–3.69 (2H, m), 2.20 (2H, q, *J* = 7.3 Hz), 1.86–1.79 (2H, m)); **^{13}C NMR** (125 MHz, $CDCl_3$) δ 168.5, 137.6, 133.8, 131.4, 130.6, 129.1, 128.6, 127.2, 126.2, 37.7, 30.2, 28.0; **IR** (neat, cm^{-1}) 3028 (m), 2968 (m), 2931 (m), 1494 (m), 1450 (m), 1280 (m), 967 (s), 748 (s), 694 (s); **MP** 69–71 °C; **HRMS** (ESI$^+$) [M+H]$^+$ calc'd for $C_{21}H_{20}NO_2$: 318.1489, found: 318.1493.

3. Preparation of substituted malononitrile derivatives

2-methylmalononitrile (**4a**), 2-benzylmalononitrile (**4b**), and 2-cinnamylmalononitrile (**4c**) were prepared by the alkylation of malononitrile as previously described.[5]

III. Supplemental Screening Data

Table S1. Other bases screened

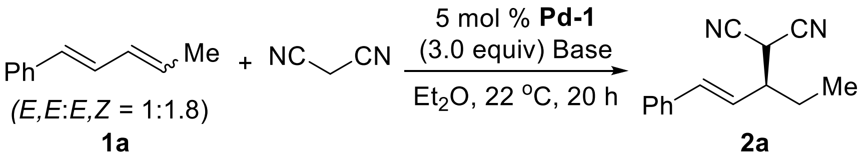

entry	Base	yield (%)[a]	er[b]
1	Et_3N	24	99.5:0.5
2	DBU	<5	-
3	(*i*-Pr)$_2$NEt	<5	-
4	pyridine	<5	-
5	LiO*t*-Bu	0	-
6	K_2CO_3	0	-

Reaction conditions: **1a** (0.4 mmol), malononitrile (0.2 mmol), Et_3N (0.6 mmol) and **Pd-1** were allowed to stir in solvent (0.2 mL, 1.0 M) at room temperature under N_2.[a]Isolated yield. [b]Enantiomeric ratio determined by HPLC analysis of purified products in comparison with authentic racemic standards.

Table S2. Equivalents of **Pd-1** and $Et_3N{\cdot}HBAr^F_4$

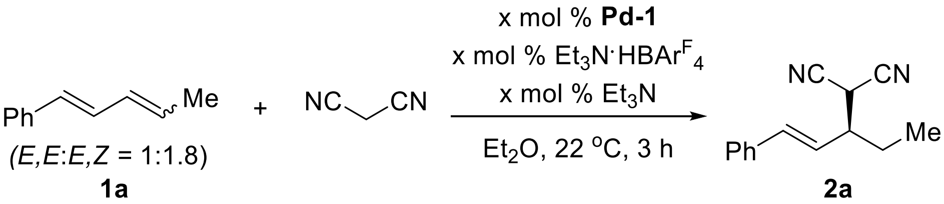

entry	**Pd-1** (mol %)	Et_3N (equiv)	$Et_3N{\cdot}HBAr^F_4$ (mol %)	yield (%)a[b]	er[b]
1	5	0	0	<5	-
2	5	1.0	5	39	99.99:0.01
3	5	3.0	5	67	99.5:0.5
4	7	3.0	7	93	99.5:0.5
5	10	3.0	10	93	98.5:1.5

Reaction conditions: **1a** (0.4 mmol), malononitrile (0.2 mmol), Et_3N (0.6 mmol) and **Pd-1** were allowed to stir in solvent (0.2 mL, 1.0 M) at room temperature under N_2.[a]Isolated yield. [b]Enantiomeric ratio determined by HPLC analysis of purified products in comparison with authentic racemic standards.

Table S3. Further examination of equivalents of $Et_3N\cdot HBAr^F{}_4$

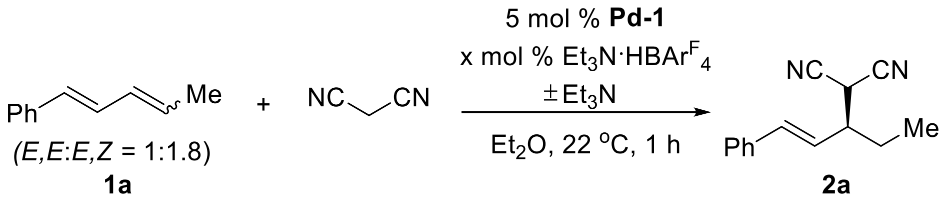

entry	Et_3N (equiv)	$Et_3N\cdot HBAr^F{}_4$ (mol %)	yield (%)[a]	er[b]
1	3.0	5	67	99.5:0.5
2	3.0	10	67	99:1
3	3.0	20	74	99:1
4	3.0	100	91(9)[c]	99.5:0.5
5	0	100	N.R.	-

Reaction conditions: **1a** (0.4 mmol), malononitrile (0.2 mmol), Et_3N (0.6 mmol) and **Pd-1** were allowed to stir in solvent (0.2 mL, 1.0 M) at room temperature under N_2.[a]Isolated yield. [b]Enantiomeric ratio determined by HPLC analysis of purified products in comparison with authentic racemic standard. [c]Yield of bis-alkylated product.

Table S4. Equivalents of Et_3N

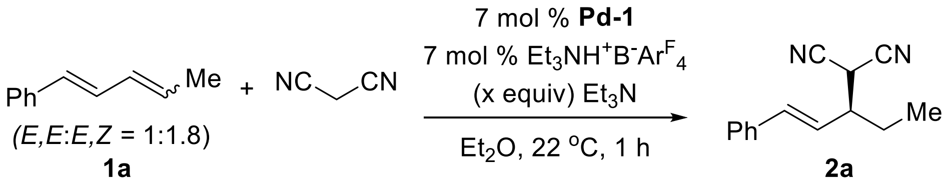

entry	Et_3N (equiv)	yield (%)[a]	er[b]
1	0.07	52(4)[c]	99.5:0.5
2	0.20	71(11)[c]	99:1
3	0.50	62(14)[c]	98.5:1.5
4	1.0	62(17)[c]	98.5:1.5
5	2.0	93(3)[c]	99.5:0.5
6	3.0	93	99.5:0.5

Reaction conditions: **1a** (0.4 mmol), malononitrile (0.2 mmol), Et_3N (0.6 mmol) and **Pd-1** were allowed to stir in solvent (0.2 mL, 1.0 M) at room temperature under N_2.[a]Isolated yield. [b]Enantiomeric ratio determined by HPLC analysis of purified products in comparison with authentic racemic standard. [c]Yield of bis-alkylated product.

An additional experiment was performed using 5.0 equiv of **1a** and 1.0 equiv of Et_3N in an attempt to force conversion to the bis-alkylated product **3a**; however, the mono-alkylated product **2a** was still isolated as the major product.

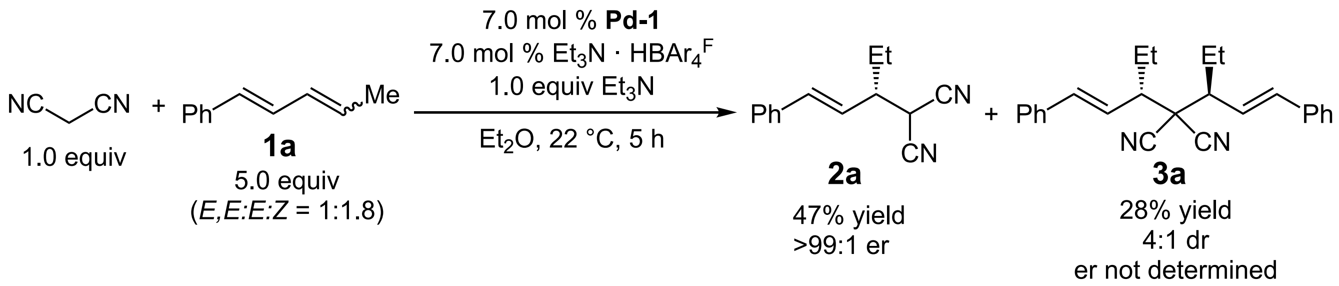

Table S5. Other PHOX ligands screened

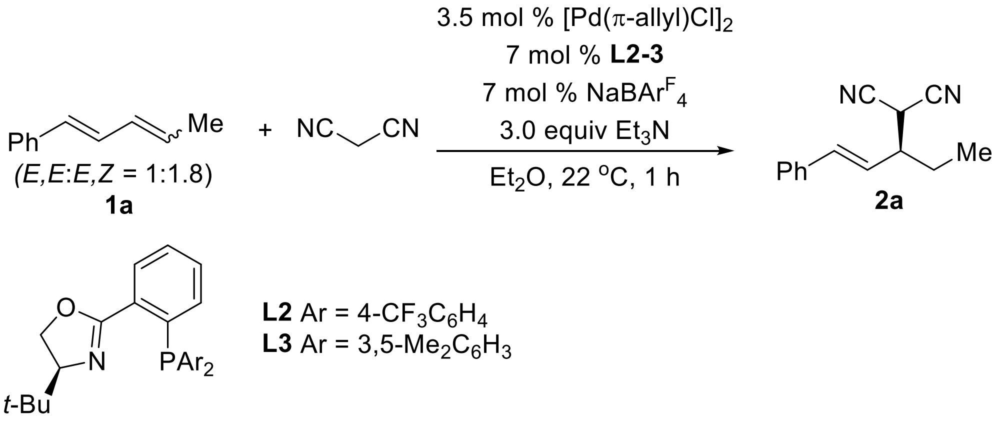

entry	PHOX ligands	yield (%)[a]	er[b]
1	**L2**	62(30)[c]	93:7
2	**L3**	12	85.5:14.5

Reaction conditions: **1a** (0.4 mmol), malononitrile (0.2 mmol) and Et_3N (0.6 mmol) in the presence of $[Pd(\pi\text{-allyl})Cl]_2$ (3.5 mol %), PHOX ligands (7 mol %) and $NaBAr^F_4$ (7 mol %) were allowed to stir in Et_2O (0.2 mL) under N_2. [a]Isolated yield. [b]Enantiomeric ratio determined by HPLC analysis of purified products in comparison with authentic racemic standard. [c]Yield of bis-alkylated product.

IV. Substrate Scope

Hydroalkylation of Internal Dienes

General Method: Inside an N_2-filled glovebox, to a microwave vial equipped with a magnetic stirring rod were added successively: **Pd-1** (0.014 mmol, 7.0 mol %), appropriate nucleophile (0.20 mmol, 1.0 equiv), Et_2O (0.2 mL), appropriate diene (0.40 mmol, 2.0 equiv), and lastly Et_3N (84 μL, 0.60 mmol, 3.0 equiv). Reactions were allowed to stir at room temperature for the specified amount of time. The solution was then concentrated in vacuo and the unpurified material was analyzed by ^{1}H NMR to determine the regiomeric ratio and diastereomeric ratio when appropriate. The material was purified by flash silica gel chromatography.

The results for the substrate scope in the manuscript are an average of two runs, but for clarity, the results shown below are one of the two runs.

Products **2** and **5** contain <5% of the allylated starting material pronucleophile that is generated upon precatalyst activation and could not be separated from the desired product. [6]

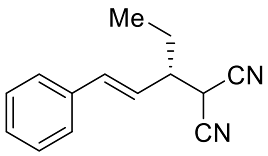

2a

(*S*,*E*)-2-(1-phenylpent-1-en-3-yl)malononitrile (2a): Prepared by the General Method using **Pd-1** at 22 ˚C for 1 h. The material was purified by flash silica gel chromatography (100% hexanes to 80:20 hexanes:EtOAc) to yield **2a** as a colorless oil (39.2 mg, 0.186 mmol, 93.0% yield). **^{1}H NMR** (400 MHz, $CDCl_3$) δ 7.40 (2H, d, J = 8.0 Hz), 7.37–7.25 (3H, m), 6.67 (1H, d, J = 15.7 Hz), 5.99 (1H, dd, J = 15.7, 9.3 Hz), 3.78 (1H, d, J = 5.3 Hz), 2.76–2.69 (1H, m), 1.90–1.80 (1H, m), 1.77–1.65 (1H, m), 1.01 (3H, t, J = 7.3 Hz); **^{13}C NMR** (125 MHz, $CDCl_3$) δ 136.4, 135.6, 128.8, 128.5, 126.7, 124.6, 111.9, 111.7, 46.4, 28.8, 25.5, 11.5; **IR** (neat, cm^{-1}) 3028 (m), 2968 (m), 2931 (m), 1494 (m), 1450 (m), 1280 (m), 967 (s), 748 (s), 694 (s); **HRMS** (ESI^{+}) $[M+H]^{+}$ calc'd for $C_{14}H_{15}N_2$: 211.1230, found: 211.1231. $[\alpha]_D^{27}$ = –15.0 (c = 1.0, $CHCl_3$) for a sample of 99.5:0.5 er.

HPLC: Column: Lux Amylose-2 (3 μm, 4.60 mm X 250 mm). Mobile phase: 10:90 *i*-PrOH:Hexanes, 1.0 mL/min. Detection wavelength: 254 nm. Er = 99.5:0.5.

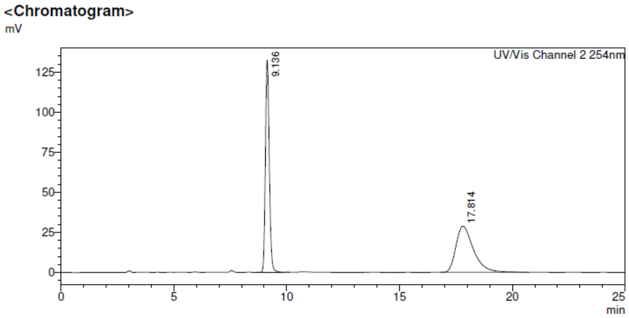

<Peak Table>
UV/Vis Channel 2 254nm

Peak#	Ret. Time	Area	Area%
1	9.136	1585693	50.407
2	17.814	1560103	49.593
Total		3145796	100.000

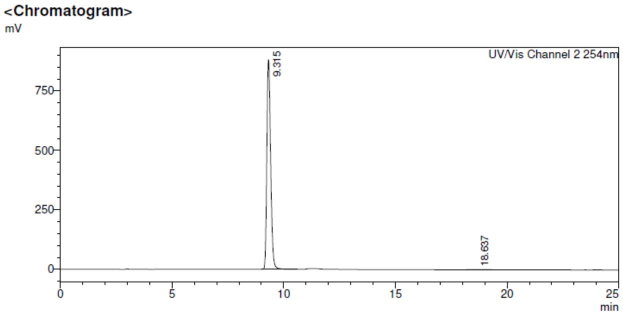

<Peak Table>
UV/Vis Channel 2 254nm

Peak#	Ret. Time	Area	Area%
1	9.315	10414359	99.403
2	18.637	62537	0.597
Total		10476897	100.000

Bis-alkylated side product:

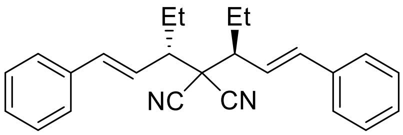

3a

2,2-Bis((*S*,*E*)-1-phenylpent-1-en-3-yl)malononitrile (3a): The material was purified by flash silica gel chromatography (100% hexanes to 80:20 hexanes:EtOAc) to yield **3a** as a colorless oil. **^{1}H NMR** (500 MHz, $CDCl_3$) δ 7.41–7.30 (10H, m), 6.45 (2H, d, J = 15.8 Hz), 5.89 (2H, dd, J = 15.7, 9.9 Hz), 2.60–2.54 (2H, m), 2.09–2.04 (2H, m), 1.77–1.67 (2H, m), 0.92 (6H, t, J = 7.3 Hz); **^{13}C NMR** (125 MHz, $CDCl_3$) δ 137.1, 135.8, 129.0, 128.6, 126.6, 123.3, 114.7, 50.3, 47.0, 25.1, 11.7; **IR** (neat, cm^{-1}) 3026 (w), 2965 (m), 2930 (m), 2875 (m), 1492 (m), 1451 (s), 967 (s), 751 (s), 691 (s); **HRMS** (ESI^+) $[M+H]^+$ calc'd for $C_{25}H_{27}N_2$: 355.2169, found: 355.2172.

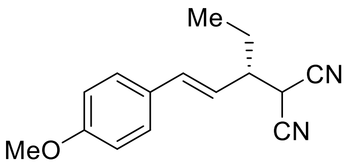

2b

(*S*,*E*)-2-(1-(4-methoxyphenyl)pent-1-en-3-yl)malononitrile (2b): Prepared by The General Method using **Pd-1** at 22 ˚C for 1 h. The material was purified by flash silica gel chromatography (100% hexanes to 90:10 hexanes:EtOAc) to yield **2b** as a colorless oil (32.0 mg, 0.133 mmol, 66.6 yield). **^{1}H NMR** (400 MHz, $CDCl_3$) δ 7.33 (2H, d, J = 8.7 Hz), 6.86 (2H, d, J = 8.7 Hz), 6.60 (1H, d, J = 15.7 Hz), 5.83 (1H, dd, J = 15.7, 9.3 Hz), 3.80 (3H, s), 3.76 (1H, d, J = 5.3 Hz), 2.75–2.62 (1H, m), 1.91–1.77 (1H, m), 1.76–1.60 (1H, m), 0.99 (3H, t, J = 7.4 Hz); **^{13}C NMR** (125 MHz, $CDCl_3$) δ 159.9, 135.7, 128.4, 128.0, 122.3, 114.1, 112.0, 111.8, 55.4, 46.4, 28.9, 25.5, 11.5; **IR** (neat, cm^{-1}) 2966 (m), 2933 (m), 2838 (w), 1577 (s), 1606 (w), 1511 (s), 1462 (m), 1248 (s), 1175 (s), 1030 (s), 968 (s), 819 (s); **HRMS** (ESI$^+$) $[M+H]^+$ calc'd for $C_{15}H_{17}N_2O$: 241.1337, found: 241.1337.
$[\alpha]_D^{27}$ = –15.1 (c = 1.0, $CHCl_3$) for a sample of 99:1 er.

HPLC: Column: Lux Amylose-2 (3 μm, 4.60 mm X 250 mm). Mobile phase: 10:90 *i*-PrOH:Hexanes, 0.3 mL/min. Detection wavelength: 254 nm. Er = 99:1.

<Chromatogram>

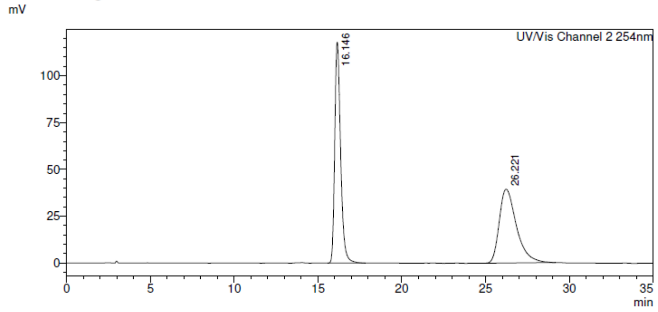

<Peak Table>
UV/Vis Channel 2 254nm

Peak#	Ret. Time	Area	Area%
1	16.146	2875505	50.489
2	26.221	2819776	49.511
Total		5695281	100.000

<Chromatogram>

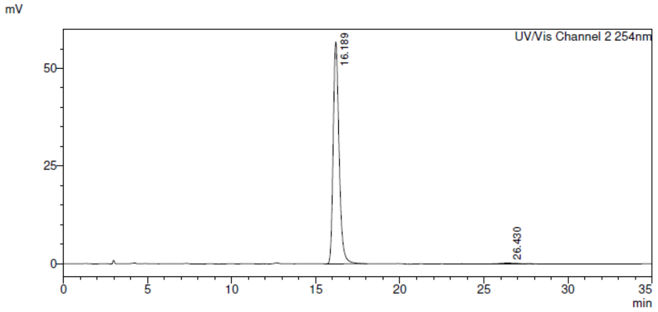

<Peak Table>
UV/Vis Channel 2 254nm

Peak#	Ret. Time	Area	Area%
1	16.189	1386012	98.797
2	26.430	16880	1.203
Total		1402892	100.000

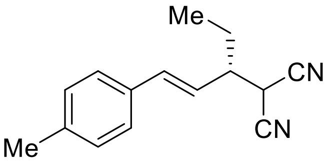

2c

(*S*,*E*)-2-(1-(*p*-tolyl)pent-1-en-3-yl)malononitrile (2c): Prepared by The General Method using **Pd-1** at 22 ˚C for 1 h. The material was purified by flash silica gel chromatography (100% hexanes to 80:20 hexanes:EtOAc) to yield **2c** as a colorless oil (24.0 mg, 0.107 mmol, 54.0% yield). **^{1}H NMR** (400 MHz, $CDCl_3$) δ 7.29 (2H, d, J = 8.1 Hz), 7.14 (2H, d, J = 7.9 Hz), 6.63 (1H, d, J = 15.7 Hz), 5.93 (1H, dd, J = 15.7, 9.3 Hz), 3.76 (1H, d, J = 5.3 Hz), 2.74–2.67 (1H, m), 2.34 (3H, s), 1.94–1.84 (1H, m), 1.80–1.69 (1H, m), 1.00 (3H, t, J = 7.4 Hz); **^{13}C NMR** (125 MHz, $CDCl_3$) δ 138.5, 136.2, 132.9, 129.4, 126.7, 123.6, 111.9, 111.8, 46.4, 28.9, 25.5, 21.3, 11.5; **IR** (neat, cm^{-1}) 2967 (m), 2923 (m), 1513 (s), 1455 (m), 1114 (w), 968 (s), 804 (s); **HRMS** (ESI^{+}) [M+H]$^{+}$ calc'd for $C_{15}H_{17}N_2$: 225.1386, found: 225.1385.

$[\alpha]_D^{27}$ = −15.1 (c = 1.0, $CHCl_3$) for a sample of 98:2 er.

HPLC: Column: Lux Amylose-2 (3 μm, 4.60 mm X 250 mm). Mobile phase: 10:90 *i*-PrOH:Hexanes, 1.0 mL/min. Detection wavelength: 254 nm. Er = 98:2.

<Chromatogram>

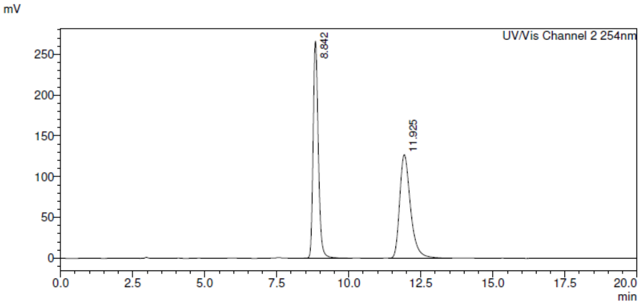

<Peak Table>

UV/Vis Channel 2 254nm

Peak#	Ret. Time	Area	Area%
1	8.842	3353676	50.275
2	11.925	3316926	49.725
Total		6670602	100.000

<Chromatogram>

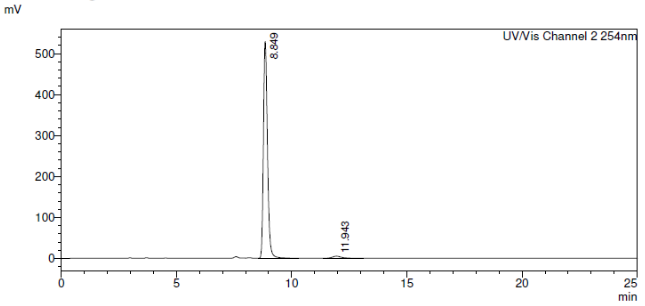

<Peak Table>

UV/Vis Channel 2 254nm

Peak#	Ret. Time	Area	Area%
1	8.849	6420840	97.938
2	11.943	135177	2.062
Total		6556016	100.000

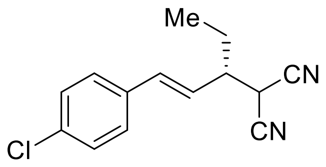

2d

(*S*,*E*)-2-(1-(4-chlorophenyl)pent-1-en-3-yl)malononitrile (2d): Prepared by General Method using **Pd-1** at 22 ˚C for 1 h. The material was purified by flash silica gel chromatography (100% hexanes to 80:20 hexanes:EtOAc) to yield **2d** as a colorless oil (19.6 mg, 0.080 mmol, 40.0% yield). **^{1}H NMR** (400 MHz, $CDCl_3$) δ 7.38–7.27 (4H, m), 6.62 (1H, d, J = 15.7 Hz), 5.96 (1H, dd, J = 15.8, 9.3 Hz), 3.78 (1H, d, J = 5.3 Hz), 2.76–2.68 (1H, m), 1.91–1.79 (1H, m), 1.78–1.65 (1H, m), 1.00 (3H, t, J = 7.4 Hz); **^{13}C NMR** (125 MHz, $CDCl_3$) δ 135.2, 134.3, 134.1, 129.0, 128.0, 125.3, 111.9, 111.6, 46.4, 28.7, 25.5, 11.5; **IR** (neat, cm^{-1}) 2962 (m), 2930 (m), 2912 (w), 2873 (w), 1491 (s), 1357 (m), 1113 (s), 993 (s), 946 (m), 812 (s); **HRMS** (ESI^{+}) $[M+H]^{+}$ calc'd for $C_{14}H_{13}ClN_2$: 245.0840, found: 245.0836.
$[\alpha]_D^{27}$ = –15.1 (c = 1.0, $CHCl_3$) for a sample of 96:4 er.

HPLC: Column: Lux Amylose-2 (3 μm, 4.60 mm X 250 mm). Mobile phase: 10:90 *i*-PrOH:Hexanes, 1.0 mL/min. Detection wavelength: 254 nm. Er = 96:4.

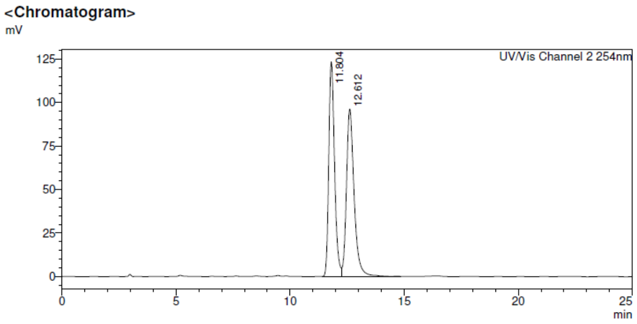

<Peak Table>
UV/Vis Channel 2 254nm

Peak#	Ret. Time	Area	Area%
1	11.804	2167621	49.183
2	12.612	2239640	50.817
Total		4407261	100.000

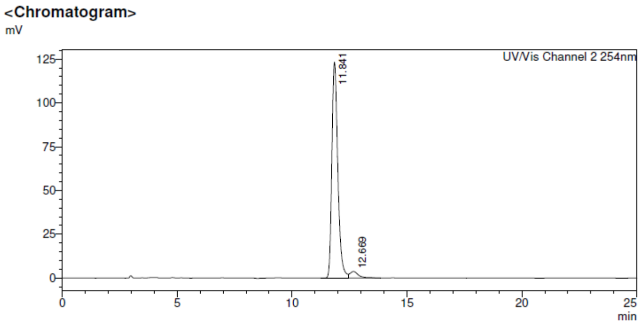

<Peak Table>
UV/Vis Channel 2 254nm

Peak#	Ret. Time	Area	Area%
1	11.841	2218480	95.710
2	12.669	99446	4.290
Total		2317926	100.000

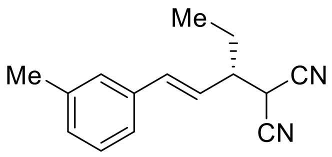

2e

(*S*,*E*)-2-(1-(*m*-Tolyl)pent-1-en-3-yl)malononitrile (2e): Prepared by The General Method using **Pd-1** at 22 ˚C for 1 h. The material was purified by flash silica gel chromatography (100% hexanes to 90:10 hexanes:EtOAc) to yield **2e** as a colorless oil (40.0 mg, 0.178 mmol, 89.2% yield). **^{1}H NMR** (400 MHz, $CDCl_3$) δ 7.25–7.10 (3H, m), 7.11 (1H, d, J = 7.1 Hz), 6.64 (1H, d, J = 15.7 Hz), 5.97 (1H, dd, J = 15.7, 9.3 Hz), 3.77 (1H, d, J = 5.3 Hz), 2.74–2.67 (1H, m), 2.35 (3H, s), 1.90–1.79 (1H, m), 1.76–1.65 (1H, m), 1.00 (3H, t, J = 7.4 Hz); **^{13}C NMR** (125 MHz, $CDCl_3$) δ 138.4, 136.5, 135.6, 129.3, 128.7, 127.4, 124.4, 124.0, 111.9, 111.7, 46.4, 28.9, 25.5, 21.4, 11.5; **IR** (neat, cm^{-1}) 2967 (m), 2924 (m), 1604 (m), 1460 (s), 1355 (w), 967 (s), 777 (s), 694 (s); **HRMS** (ESI$^+$) [M+H]$^+$ calc'd for $C_{15}H_{16}N_2$: 225.1386, found: 225.1381.

$[\alpha]_D^{27}$ = −14.8 (c = 1.0, $CHCl_3$) for a sample of 97.5:2.5 er.

HPLC: Column: Lux amylose-2 (3 μm, 4.60 mm X 250 mm). Mobile phase: 10:90 *i*-PrOH:Hexanes, 1.0 mL/min. Detection wavelength: 254 nm. Er = 97.5:2.5.

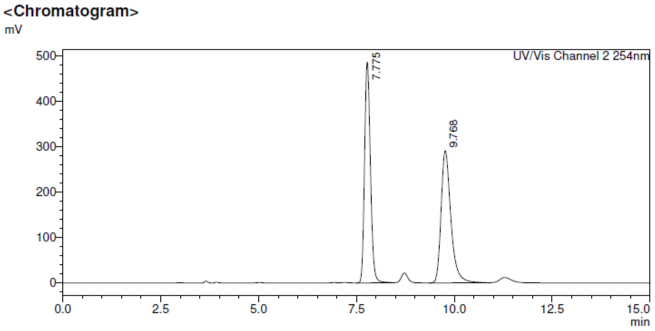

<Peak Table>
UV/Vis Channel 2 254nm

Peak#	Ret. Time	Area	Area%
1	7.775	5081417	50.026
2	9.768	5076036	49.974
Total		10157453	100.000

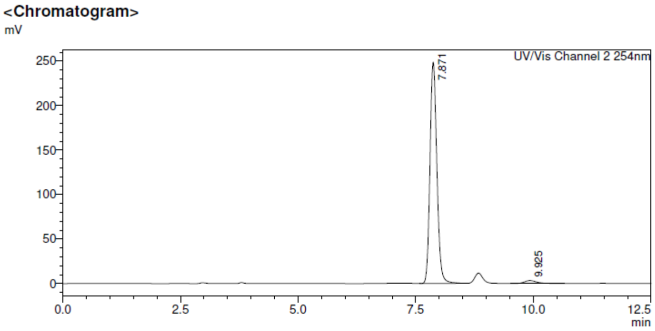

<Peak Table>
UV/Vis Channel 2 254nm

Peak#	Ret. Time	Area	Area%
1	7.871	2525611	97.712
2	9.925	59147	2.288
Total		2584758	100.000

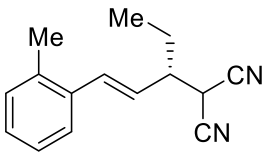

2f

(*S*,*E*)-2-(1-(*o*-Tolyl)pent-1-en-3-yl)malononitrile (2f): Prepared by The General Method using **Pd-1** at 22 ˚C for 1 h. The material was purified by flash silica gel chromatography (100% hexanes to 90:10 hexanes:EtOAc) to yield **2f** as a colorless oil (39.0 mg, 0.174 mmol, 86.9 % yield). **^{1}H NMR** (400 MHz, $CDCl_3$) δ 7.46–7.40 (1H, m), 7.23–7.14 (3H, m), 6.90 (1H, d, *J* = 15.6 Hz), 5.85 (1H, dd, *J* = 15.6, 9.3 Hz), 3.78 (1H, d, J = 5.5 Hz, 1H), 2.83–2.76 (1H, m), 2.37 (3H, s), 1.96–1.86 (1H, m), 1.82–1.71 (1H, m), 1.03 (3H, t, *J* = 7.4 Hz); **^{13}C NMR** (125 MHz, $CDCl_3$) δ 135.7, 135.1, 134.6, 130.4, 128.4, 126.3, 126.1, 112.0, 111.7, 46.5, 28.9, 25.4, 19.8, 11.5; **IR** (neat, cm^{-1}) 2967 (m), 2929 (m), 1484 (m), 1460 (s), 1356 (w), 968 (s), 750 (s); **HRMS** (ESI^{+}) $[M+H]^{+}$ calc'd for $C_{15}H_{17}N_2$: 225.1386, found: 225.1386.

$[\alpha]_D^{27}$ = –14.7 (*c* = 1.0, $CHCl_3$) for a sample of 96:4 er.

HPLC: Column: Lux amylose-2 (3 μm, 4.60 mm X 250 mm). Mobile phase: 3:97 *i*-PrOH:Hexanes, 1.0 mL/min. Detection wavelength: 254 nm. Er = 96:4.

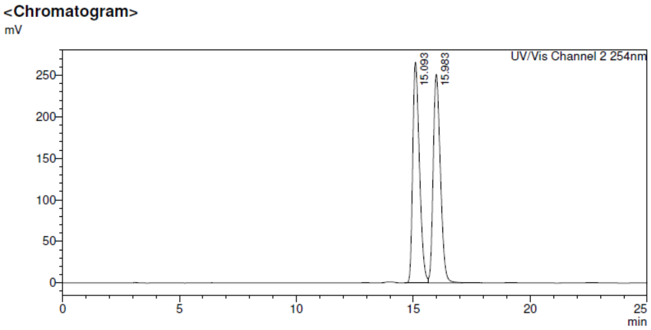

<Peak Table>
UV/Vis Channel 2 254nm

Peak#	Ret. Time	Area	Area%
1	15.093	5307988	49.652
2	15.983	5382308	50.348
Total		10690295	100.000

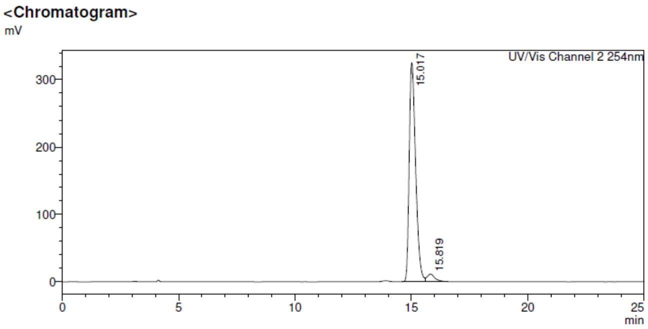

<Peak Table>
UV/Vis Channel 2 254nm

Peak#	Ret. Time	Area	Area%
1	15.017	6230733	95.886
2	15.819	267344	4.114
Total		6498077	100.000

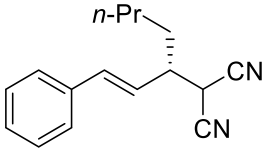

2g

(*S*,*E*)-2-(1-phenylhex-1-en-3-yl)malononitrile (2g): Prepared by General Method using **Pd-1** at 22 ˚C for 1 h. The material was purified by flash silica gel chromatography (100% hexanes to 80:20 hexanes:EtOAc) to yield **2g** as a colorless oil (37.2 mg, 0.156 mmol, 78.0% yield). **^{1}H NMR** (400 MHz, $CDCl_3$) δ 7.40 (2H, d, J = 7.0 Hz), 7.34 (2H, dd, J = 7.3, 7.3 Hz), 7.31–7.26 (1H, m), 6.66 (1H, d, J = 15.7 Hz), 6.00 (1H, J = 15.7, 9.3 Hz), 3.77 (1H, d, J = 5.2 Hz), 2.80 (1H, tt, J = 9.7, 4.9 Hz), 1.83–1.64 (2H, m), 1.43–1.25 (4H, m), 0.91 (3H, t, J = 6.8 Hz); **^{13}C NMR** (125 MHz, $CDCl_3$) δ 136.1, 135.6, 128.8, 128.5, 126.8, 125.0, 111.9, 111.7, 44.8, 32.0, 29.1, 29.0, 22.3, 13.9; **IR** (neat, cm^{-1}) 3028 (w), 2957 (m), 2930 (m), 2859 (m), 1495 (m), 1450 (m), 967 (s), 749 (s), 694 (s); **HRMS** (ESI^{+}) $[M+H]^{+}$ calc'd for $C_{16}H_{19}N_2$: 239.1543, found: 239.1541.
$[\alpha]_D^{27}$ = –15.0 (c = 1.0, $CHCl_3$) for a sample of 95.5:4.5 er.

HPLC: Column: Lux Amylose-2 (3 μm, 4.60 mm X 250 mm). Mobile phase: 10:90 *i*-PrOH:Hexanes, 1.0 mL/min. Detection wavelength: 254 nm. Er = 95.5:4.5.

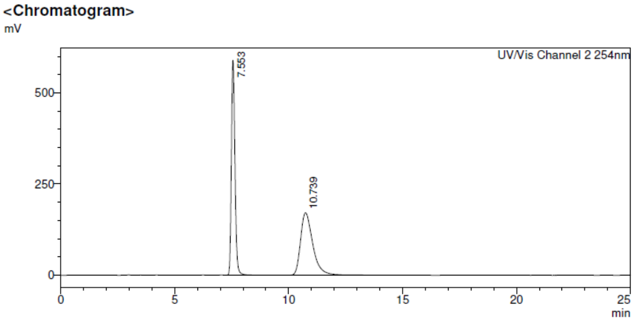

<Peak Table>
UV/Vis Channel 2 254nm

Peak#	Ret. Time	Area	Area%
1	7.553	6413052	49.993
2	10.739	6414741	50.007
Total		12827794	100.000

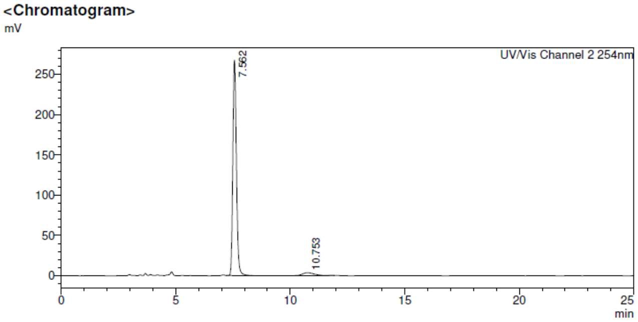

<Peak Table>
UV/Vis Channel 2 254nm

Peak#	Ret. Time	Area	Area%
1	7.562	2896427	95.628
2	10.753	132406	4.372
Total		3028833	100.000

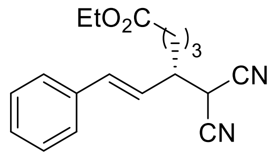

2h

Ethyl (*S*,*E*)-5-(dicyanomethyl)-7-phenylhept-6-enoate (2h): Prepared by The General Method using **Pd-1** at 22 ˚C for 1 h. The material was purified by flash silica gel chromatography (100% hexanes to 80:20 hexanes:EtOAc) to yield **2h** as a colorless oil (37.0 mg, 0.125 mmol, 62.4% yield). **^{1}H NMR** (500 MHz, $CDCl_3$) δ 7.39 (2H, d, J = 7.2 Hz), 7.33 (2H, t, J = 7.3 Hz), 7.28 (1H, t, J = 7.2 Hz), 6.68 (1H, d, J = 15.7 Hz), 5.99 (1H, dd, J = 15.7, 9.3 Hz), 4.12 (2H, q, J = 7.1 Hz), 3.80 (1H, d, J = 5.2 Hz), 2.85–2.79 (1H, m), 2.34 (2H, td, J = 6.8, 2.9 Hz), 1.85–1.58 (4H, m), 1.24 (3H, t, J = 7.1 Hz); **^{13}C NMR** (125 MHz, $CDCl_3$) δ 172.8, 136.7, 135.5, 128.8, 128.7, 126.8, 124.3, 111.7, 60.7, 44.7, 33.6, 31.6, 29.1, 22.3, 14.3; **IR** (neat, cm^{-1}) 2908 (m), 1724 (s), 1449 (m), 1184 (s), 1154 (s), 1028 (m), 968 (s), 750 (s), 695 (s); **HRMS** (ESI^{+}) $[M+H]^{+}$ calc'd for $C_{18}H_{20}N_2O_2$: 297.1598, found: 297.1603.
$[\alpha]_D^{27}$ = –14.8 (c = 1.0, $CHCl_3$) for a sample of 95.5:4.5 er.

HPLC: Column: Lux amylose-1 (3 μm, 4.60 mm X 250 mm). Mobile phase: 5:95 *i*-PrOH:Hexanes, 1.0 mL/min. Detection wavelength: 254 nm. Er = 95.5:4.5.

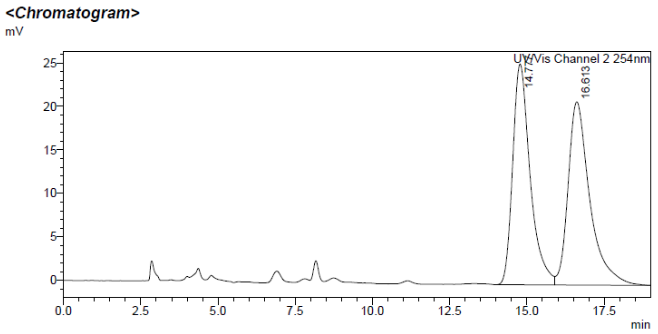

<Peak Table>
UV/Vis Channel 2 254nm

Peak#	Ret. Time	Area	Area%
1	14.777	999355	49.230
2	16.613	1030616	50.770
Total		2029971	100.000

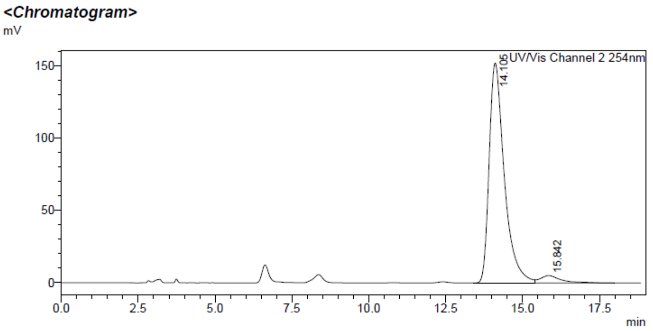

<Peak Table>
UV/Vis Channel 2 254nm

Peak#	Ret. Time	Area	Area%
1	14.105	5200053	95.598
2	15.842	239446	4.402
Total		5439498	100.000

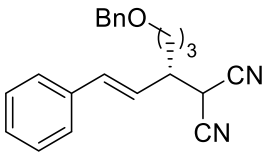

2i

(*S*,*E*)-2-(6-(Benzyloxy)-1-phenylhex-1-en-3-yl)malononitrile (2i): Prepared by The General Method using **Pd-1** at 22 ˚C for 1 h. The material was purified by flash silica gel chromatography (100% hexanes to 80:20 hexanes:EtOAc) to yield **2i** as a colorless oil (50.0 mg, 0.151 mmol, 75.7% yield). **^{1}H NMR** (500 MHz, $CDCl_3$) δ 7.39–7.26 (10H, m), 6.62 (1H, d, J = 15.7 Hz), 5.98 (1H, dd, J = 15.7, 9.3 Hz), 4.43 (1H, d, J_{AB} = 11.9 Hz), 4.42 (1H, d, J_{AB} = 11.9 Hz), 3.82 (1H, d, J = 5.1 Hz), 3.49 (2H, t, J = 5.8 Hz), 2.86–2.80 (1H, m), 1.94–1.85 (1H, m), 1.82–1.69 (2H, m), 1.67–1.58 (1H, m); **^{13}C NMR** (125 MHz, $CDCl_3$) δ 138.2, 136.5, 135.6, 128.8, 128.6, 128.6, 127.8, 126.8, 124.6, 111.9, 111.6, 73.2, 69.3, 44.6, 29.4, 29.0, 27.1 (one sp^2 carbon missing due to overlap); **IR** (neat, cm^{-1}) 2908 (m), 1724 (s), 1449 (m), 1184 (s), 1154 (s), 1028 (m), 968 (s), 750 (s), 695 (s); **HRMS** (ESI$^+$) [M+H]$^+$ calc'd for $C_{23}H_{23}N_2O$: 331.1805, found: 331.1811.
$[\alpha]_D^{27}$ = −14.7 (c = 1.0, $CHCl_3$) for a sample of 99.5:0.5 er.

HPLC: Column: Lux amylose-2 (3 μm, 4.60 mm X 250 mm). Mobile phase: 5:95 *i*-PrOH:Hexanes, 1.0 mL/min. Detection wavelength: 254 nm. Er = 99.5:0.5.

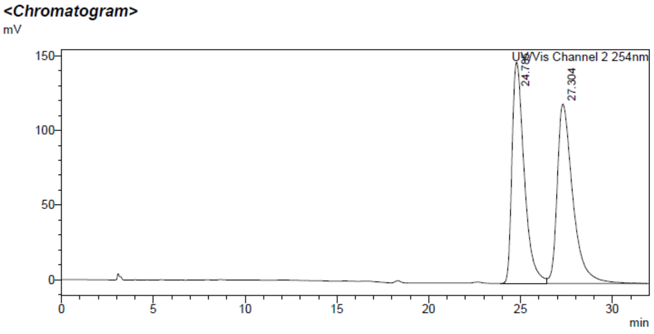

<Peak Table>
UV/Vis Channel 2 254nm

Peak#	Ret. Time	Area	Area%
1	24.785	6855604	49.029
2	27.304	7127289	50.971
Total		13982894	100.000

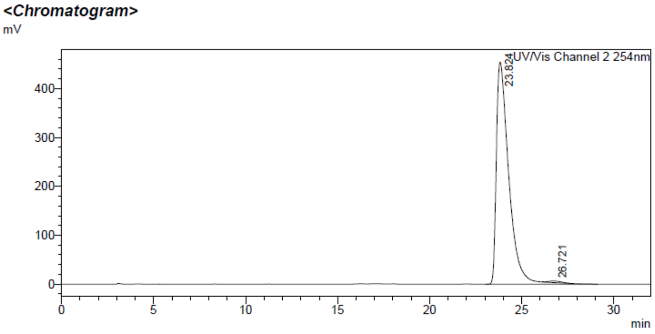

<Peak Table>
UV/Vis Channel 2 254nm

Peak#	Ret. Time	Area	Area%
1	23.824	21408564	99.310
2	26.721	148823	0.690
Total		21557387	100.000

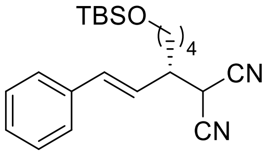

2j

(*S*,*E*)-2-(7-((*tert*-butyldimethylsilyl)oxy)-1-phenylhept-1-en-3-yl)malononitrile (2j): Prepared by The General Method using **Pd-1** at 22 ˚C for 20 h. The material was purified by flash silica gel chromatography (100% hexanes to 90:10 hexanes:EtOAc) to yield **2j** as a colorless oil (60.0 mg, 0.163 mmol, 81.4% yield). **^{1}H NMR** (400 MHz, $CDCl_3$) δ 7.39 (2H, d, J = 6.9 Hz), 7.36–7.26 (3H, m), 6.66 (1H, d, J = 15.7 Hz), 5.99 (1H, dd, J = 15.8, 9.3 Hz), 3.78 (1H, d, J = 5.2 Hz), 3.59 (2H, t, J = 6.0 Hz), 2.85–2.78 (1H, m), 1.82–1.68 (2H, m), 1.58–1.34 (4H, m), 0.85 (9H, s), 0.01 (6H, s); **^{13}C NMR** (125 MHz, $CDCl_3$) δ 136.3, 135.6, 128.8, 128.6, 126.8, 124.8, 111.9, 111.7, 62.6, 44.9, 32.2, 32.1, 29.1, 26.0, 23.4, 18.4, -5.3; **IR** (neat, cm^{-1}) 2928 (s), 2856 (m), 1462 (m), 1252 (s), 1097 (s), 967 (s), 834 (s), 774 (s), 748 (s), 694 (s); **HRMS** (ESI$^+$) $[M+H]^+$ calc'd for $C_{22}H_{33}N_3OSi$: 369.2357, found: 369.2360.
$[\alpha]_D^{27}$ = –15.1 (c = 1.0, $CHCl_3$) for a sample of 96:4 er.

HPLC: Column: Lux Amylose-2 (3 μm, 4.60 mm X 250 mm). Mobile phase: 10:90 *i*-PrOH:Hexanes, 0.3 mL/min. Detection wavelength: 254 nm. Er = 96:4.

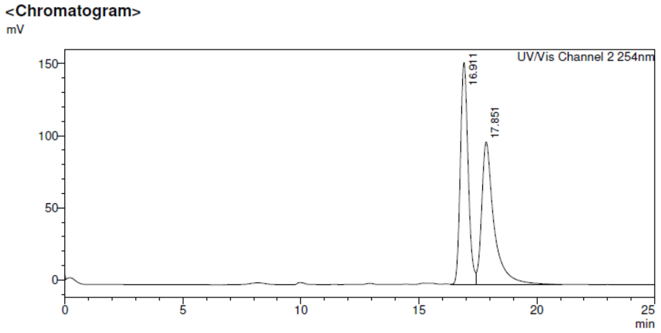

<Peak Table>
UV/Vis Channel 2 254nm

Peak#	Ret. Time	Area	Area%
1	16.911	3510119	49.486
2	17.851	3583019	50.514
Total		7093139	100.000

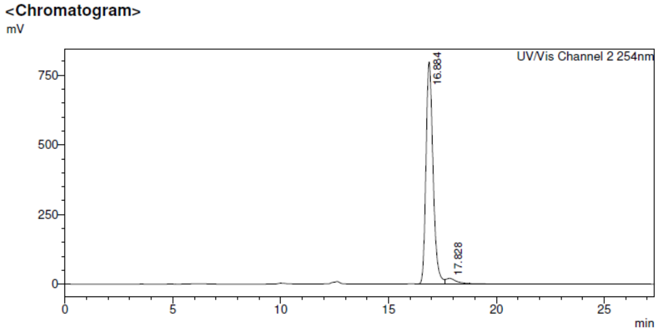

<Peak Table>
UV/Vis Channel 2 254nm

Peak#	Ret. Time	Area	Area%
1	16.884	18201887	95.999
2	17.828	758604	4.001
Total		18960491	100.000

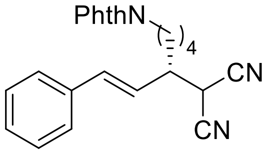

2k

(*S*,*E*)-2-(7-(1,3-dioxoisoindolin-2-yl)-1-phenylhept-1-en-3-yl)malononitrile (2k): Prepared by The General Method using **Pd-1** at 22 ˚C for 20 h. The material was purified by flash silica gel chromatography (100% hexanes to 80:20 hexanes:EtOAc) to yield **2k** as a colorless oil (38.0 mg, 0.100 mmol, 49.6% yield). **^{1}H NMR** (400 MHz, $CDCl_3$) δ 7.78 (2H, dd, *J* = 5.4, 3.0 Hz), 7.67 (2H, dd, *J* = 5.5, 3.0 Hz), 7.35 (2H, dd, *J* = 8.0, 1.5 Hz), 7.33–7.25 (3H, m), 6.63 (1H, *J* = 15.7 Hz), 5.96 (1H, dd, *J* = 15.7, 9.4 Hz), 3.79 (1H, d, *J* = 5.3 Hz), 3.67 (2H, t, *J* = 7.0 Hz), 2.83–2.76 (1H, m), 1.87–1.62 (4H, m), 1.53–1.30 (2H, m); **^{13}C NMR** (125 MHz, $CDCl_3$) δ 168.4, 136.5, 135.5, 134.0, 132.0, 128.7, 128.5, 126.8, 124.5, 123.3, 111.8, 111.6, 44.7, 37.3, 31.6, 29.1, 28.0, 24.1; **IR** (neat, cm^{-1}) 2937 (w), 1704 (s), 1437 (m), 1395 (s), 1371 (m), 1241 (m), 1044 (m), 968 (m), 718 (s); **HRMS** (ESI^{+}) $[M+H]^{+}$ calc'd for $C_{24}H_{21}N_3O_2$: 384.1707, found: 384.1708.

$[\alpha]_D^{27}$ = −15.2 (*c* = 1.0, $CHCl_3$) for a sample of 90.5:9.5 er.

HPLC: Column: Lux Amylose-1 (3 μm, 4.60 mm X 250 mm). Mobile phase: 15:85 *i*-PrOH:Hexanes, 1.0 mL/min. Detection wavelength: 254 nm. Er = 90.5:9.5.

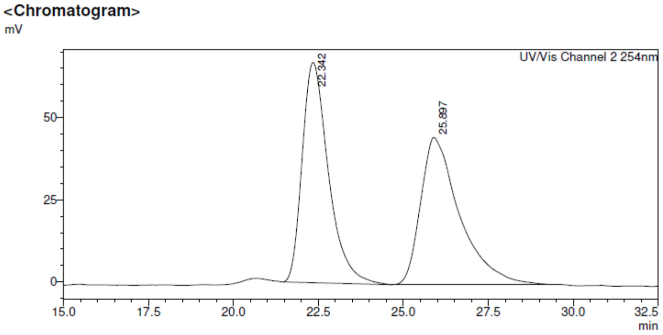

<Peak Table>
UV/Vis Channel 2 254nm

Peak#	Ret. Time	Area	Area%
1	22.342	3607326	50.191
2	25.897	3579804	49.809
Total		7187130	100.000

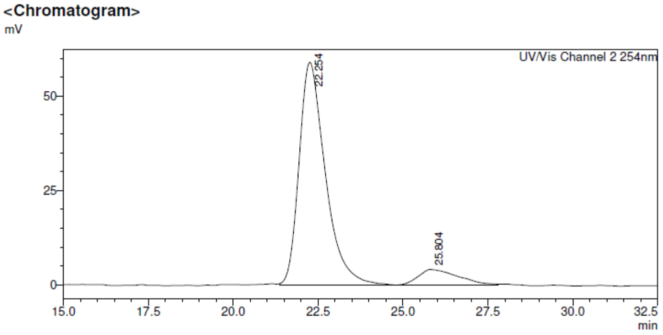

<Peak Table>
UV/Vis Channel 2 254nm

Peak#	Ret. Time	Area	Area%
1	22.254	3173875	90.451
2	25.804	335068	9.549
Total		3508944	100.000

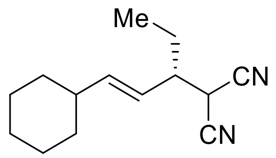

2l

(*S*,*E*)-2-(1-Cyclohexylpent-1-en-3-yl)malononitrile (2l): Prepared by The General Method using **Pd-1** at 22 ˚C for 3 h. The material was purified by flash silica gel chromatography (100% hexanes to 90:10 hexanes:EtOAc) to yield **2l** as a colorless oil (31.0 mg, 0.143 mmol, 71.6 % yield). **^{1}H NMR** (400 MHz, $CDCl_3$) δ 5.71 (1H, dd, *J* = 15.4, 6.8 Hz), 5.19 (1H, dd, *J* = 15.4, 9.1 Hz), 3.65 (1H, d, *J* = 5.6 Hz), 2.49–2.42 (1H, m), 2.04–1.96 (1H, m), 1.78–1.46 (7H, m), 1.33–1.01 (6H, m), 0.93 (3H, t, *J* = 7.4 Hz); **^{13}C NMR** (125 MHz, $CDCl_3$) δ 144.1, 122.9, 112.1, 111.9, 46.0, 40.7, 32.8, 29.1, 26.1, 25.9, 25.3, 11.4; **IR** (neat, cm^{-1}) 2966 (m), 2923 (s), 2851 (s), 1461 (s), 1350 (w), 970 (s), 891 (w); **HRMS** (ESI^+) $[M+H]^+$ calc'd for $C_{14}H_{21}N_2$: 217.1699, found: 217.1699.
$[\alpha]_D^{27}$ = –14.8 (*c* = 1.0, $CHCl_3$) for a sample of 94:6 er.

HPLC: Column: Lux amylose-2 (3 μm, 4.60 mm X 250 mm). Mobile phase: 10:90 *i*-PrOH:Hexanes, 1.0 mL/min. Detection wavelength: 200 nm. Er = 94:6.

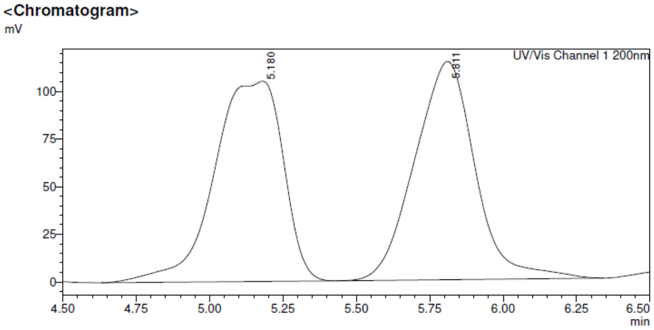

<Peak Table>
UV/Vis Channel 1 200nm

Peak#	Ret. Time	Area	Area%
1	5.180	1716708	51.085
2	5.811	1643806	48.915
Total		3360514	100.000

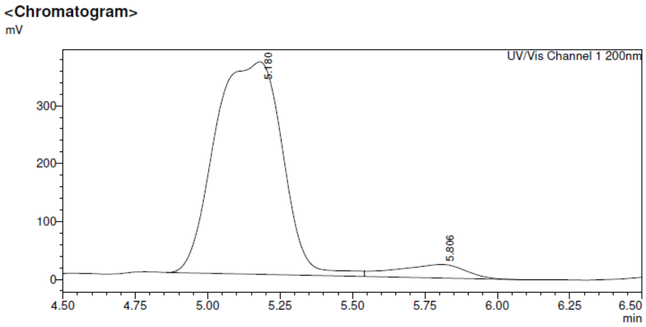

<Peak Table>
UV/Vis Channel 1 200nm

Peak#	Ret. Time	Area	Area%
1	5.180	5948483	93.968
2	5.806	381832	6.032
Total		6330315	100.000

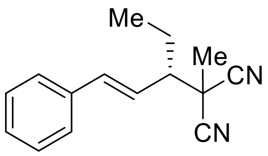

5a

(*S*,*E*)-2-Methyl-2-(1-phenylpent-1-en-3-yl)malononitrile (5a): Prepared by The General Method using **Pd-1** at 22 ˚C for 1 h. The material was purified by flash silica gel chromatography (100% hexanes to 80:20 hexanes:EtOAc) to yield **5a** as a colorless oil (44.5 mg, 0.199 mmol, 99.2% yield). **^{1}H NMR** (500 MHz, $CDCl_3$) δ 7.39 (2H, dd, *J* = 6.7, 6.7 Hz), 7.34 (2H, d, *J* = 7.4, 7.4 Hz), 7.28 (1H, t, *J* = 7.2 Hz), 6.59 (1H, d, *J* = 15.7 Hz), 5.89 (1H, dd, *J* = 15.7, 9.7 Hz), 2.45–2.39 (1H, m), 2.09–2.00 (1H, m), 1.76 (3H, s), 1.73–1.62 (1H, m), 0.99 (3H, t, *J* = 7.4 Hz); **^{13}C NMR** (125 MHz, $CDCl_3$) δ 137.5, 135.7, 128.8, 128.6, 126.7, 123.7, 116.2, 115.4, 53.1, 36.4, 24.6, 23.6, 11.9; **IR** (neat, cm^{-1}) 3028 (w), 2969 (m), 2933 (m), 2877 (w), 1451 (s), 1382 (m), 1129 (m), 970 (s), 910 (s), 755 (s), 691 (s); **HRMS** (ESI^+) $[M+H]^+$ calc'd for $C_{15}H_{17}N_2$: 225.1386, found: 225.1392.

$[\alpha]_D^{27}$ = –14.8 (*c* = 1.0, $CHCl_3$) for a sample of 96:4 er.

HPLC: Column: Lux amylose-1 (3 μm, 4.60 mm X 250 mm). Mobile phase: 5:95 *i*-PrOH:Hexanes, 0.7 mL/min. Detection wavelength: 254 nm. Er = 96:4.

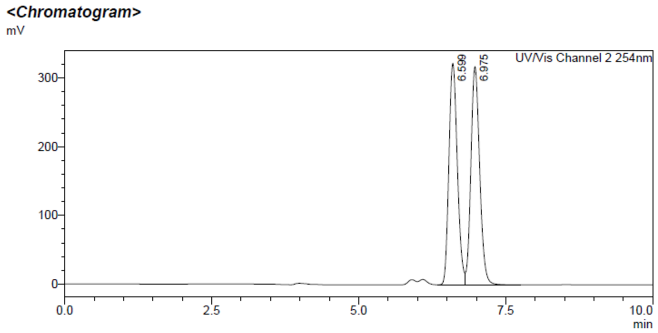

<Peak Table>

UV/Vis Channel 2 254nm

Peak#	Ret. Time	Area	Area%
1	6.599	3242115	49.427
2	6.975	3317264	50.573
Total		6559380	100.000

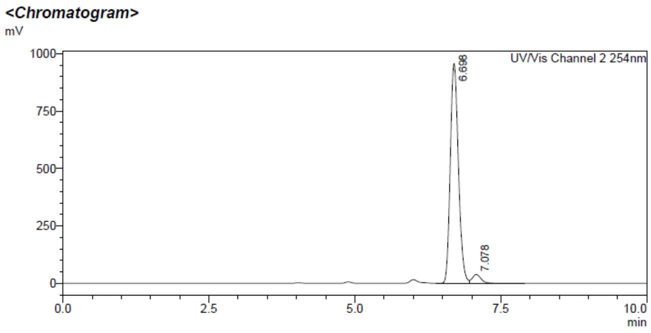

<Peak Table>

UV/Vis Channel 2 254nm

Peak#	Ret. Time	Area	Area%
1	6.698	9299039	95.803
2	7.078	407406	4.197
Total		9706444	100.000

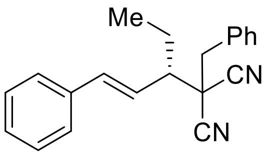

5b

(*S*,*E*)-2-Benzyl-2-(1-phenylpent-1-en-3-yl)malononitrile (5b): Prepared by General Method C using **Pd-1** at 22 ˚C for 1 h. The material was purified by flash silica gel chromatography (100% hexanes to 90:10 hexanes:EtOAc) to yield **5b** as a white solid (54.0 mg, 0.180 mmol, 89.9% yield). **^{1}H NMR** (400 MHz, $CDCl_3$) δ 7.52–7.27 (10H, m), 6.64 (1H, d, J = 15.7 Hz), 6.03 (1H, dd, J = 15.7, 9.8 Hz), 3.27 (1H d, J_{AB} = 13.8 Hz), 3.09 (1H d, J_{AB} = 13.8 Hz), 2.61–2.51 (1H, m), 2.18–2.09 (1H, m), 1.82–71 (1H, m), 1.02 (3H, t, J = 7.4 Hz); **^{13}C NMR** (125 MHz, $CDCl_3$) δ 137.5, 135.6, 132.4, 130.2, 128.9, 128.8, 128.7, 128.6, 126.7, 123.9, 115.1, 114.4, 52.1, 44.6, 42.1, 24.8, 11.8. Spectral data matched those previously reported.[7]

$[\alpha]_D^{27}$ = –15.2 (c = 1.0, $CHCl_3$) for a sample of 95.5:4.5 er.

Compound **5b** was prepared in reference 7 in enantiomerically enriched form. The sign of the rotation in that reference is the opposite to what we observed for **2a** in this work. Thus, we have assigned **2a** as the (R)-enantiomer and make all other stereochemical assignments by inference. The observed enantiomer matches that in our terminal diene hydroalkylation as well; see reference 3.

HPLC: Column: Lux cellulose-1 (3 μm, 4.60 mm X 250 mm). Mobile phase: 10:90 i-PrOH:Hexanes, 1.0 mL/min. Detection wavelength: 254 nm. Er = 95.5:4.5.

<Chromatogram>

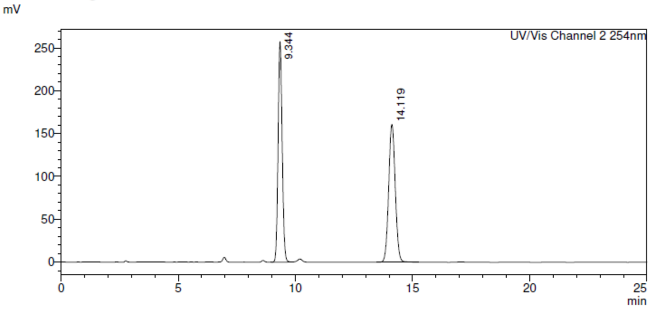

<Peak Table>

UV/Vis Channel 2 254nm

Peak#	Ret. Time	Area	Area%
1	9.344	3139523	49.876
2	14.119	3155171	50.124
Total		6294694	100.000

<Chromatogram>

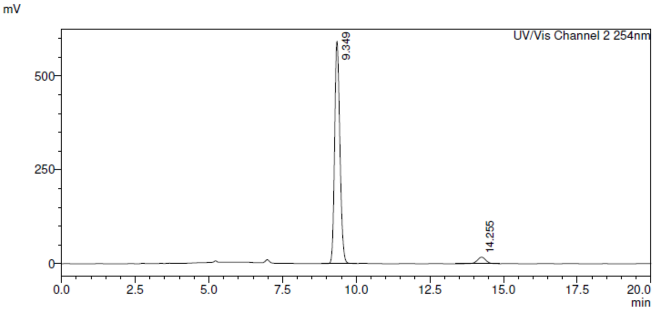

<Peak Table>

UV/Vis Channel 2 254nm

Peak#	Ret. Time	Area	Area%
1	9.349	7381907	95.532
2	14.255	345226	4.468
Total		7727132	100.000

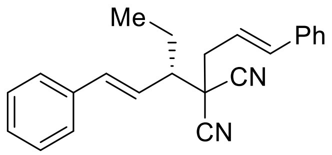

5c

2-Cinnamyl-2-((*S*,*E*)-1-(4-methoxyphenyl)pent-1-en-3-yl)malononitrile (5c): Prepared by The General Method using **Pd-1** at 22 ˚C for 1 h. The material was purified by flash silica gel chromatography (100% hexanes to 90:10 hexanes:EtOAc) to yield **5c** as a colorless oil (51.0 mg, 0.156 mmol, 78.1% yield). **^{1}H NMR** (400 MHz, $CDCl_3$) δ 7.48–7.27 (10H, m), 6.67 (1H, d, J = 15.7 Hz,), 6.62 (1H d, J = 15.7 Hz,), 6.25 (1H, dt, J = 15.4, 7.4 Hz), 5.97 (1H, dd, J = 15.7, 9.8 Hz), 2.93 (1H, dd, J_{AB} = 13.6, J_{AX} = 6.6 Hz), 2.80 (1H, dd, J_{AB} = 13.9, J_{AX} = 7.8 Hz), 2.60–2.52 (1H, m), 2.15–2.04 (1H, m), 1.81–1.70 (1H, m), 1.01 (3H, t, J = 7.3 Hz); **^{13}C NMR** (125 MHz, $CDCl_3$) δ 137.6, 137.4, 135.9, 135.6, 128.8, 128.7, 128.6, 128.4, 126.69, 126.67, 123.7, 119.4, 115.2, 114.5, 51.3, 42.7, 39.8, 24.7, 11.8; **IR** (neat, cm^{-1}) 3027 (m), 2967 (m), 2931 (m), 1494 (s), 1449 (s), 1355 (m), 966 (s), 908 (s), 731 (s), 691 (s); **HRMS** (ESI^+) $[M+NH_4]^+$ calc'd for $C_{23}H_{26}N_3$: 344.2121, found: 344.2119.
$[\alpha]_D^{27}$ = −15.7 (c = 1.0, $CHCl_3$) for a sample of 95.5:4.5 er.

HPLC: Column: Lux cellulose-1 (3 μm, 4.60 mm X 250 mm). Mobile phase: 10:90 *i*-PrOH:Hexanes, 1.0 mL/min. Detection wavelength: 254 nm. Er = 95.5:4.5.

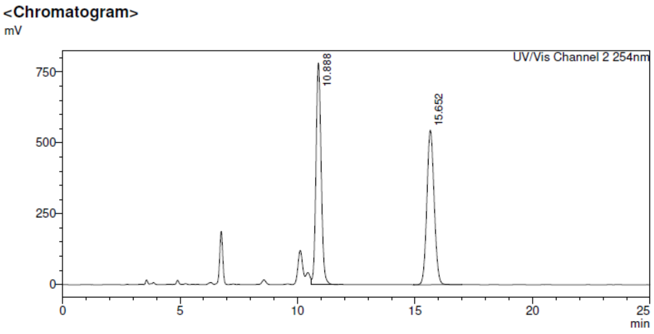

<Peak Table>
UV/Vis Channel 2 254nm

Peak#	Ret. Time	Area	Area%
1	10.888	11938209	50.416
2	15.652	11741344	49.584
Total		23679553	100.000

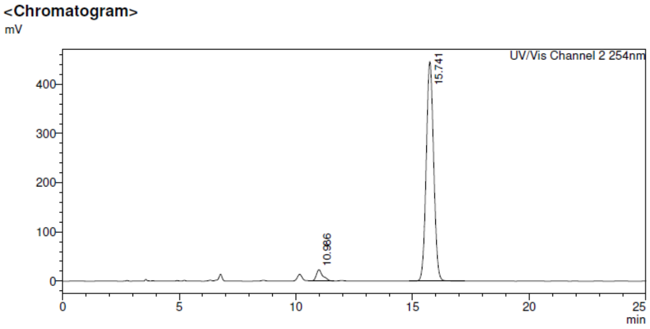

<Peak Table>
UV/Vis Channel 2 254nm

Peak#	Ret. Time	Area	Area%
1	10.986	447330	4.350
2	15.741	9835190	95.650
Total		10282520	100.000

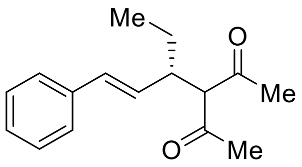

5d

(*S,E*)-3-(1-Phenylpent-1-en-3-yl)pentane-2,4-dione (5d): Prepared by The General Method using **Pd-1** at 22 ˚C for 20 h. The material was purified by flash silica gel chromatography (100% hexanes to 90:10 hexanes:EtOAc) to yield **5d** as a white solid (25.0 mg, 0.102 mmol, 51.2 % yield). **^{1}H NMR** (400 MHz, $CDCl_3$) δ 7.36–7.25 (4H, m), 7.22–7.16 (1H, m), 6.42 (1H, d, J = 15.8 Hz), 5.82 (1H, dd, J = 15.8, 9.5 Hz), 3.76 (1H, d, J = 10.6 Hz), 2.98–2.89 (1H, m), 2.21 (3H, s), 2.09 (3H, s), 1.49–1.41 (1H, m), 1.30–1.20 (1H, m), 0.87 (3H, t, J = 7.4 Hz); **^{13}C NMR** (125 MHz, $CDCl_3$) δ 203.7, 203.6, 136.8, 133.0, 129.0, 128.6, 127.6, 126.3, 74.7, 45.4, 30.3, 29.7, 26.1, 11.6; **IR** (neat, cm^{-1}) 2936 (m), 1688 (s), 1459 (m), 1356 (s), 1277 (s), 1198 (s), 1151 (s), 982 (s), 743 (s), 687 (s); **MP =** 37–38 ^{o}C; **HRMS** (ESI^{+}) $[M+NH_4]^{+}$ calc'd for $C_{16}H_{24}NO_2$: 262.1802, found: 262.1801.

$[\alpha]_D^{27}$ = –17.3 (c = 1.0, $CHCl_3$) for a sample of 92.5:7.5 er.

HPLC: Column: Lux cellulose-3 (3 μm, 4.60 mm X 250 mm). Mobile phase: 5:95 i-PrOH:Hexanes, 1.0 mL/min. Detection wavelength: 254 nm. Er = 92.5:7.5.

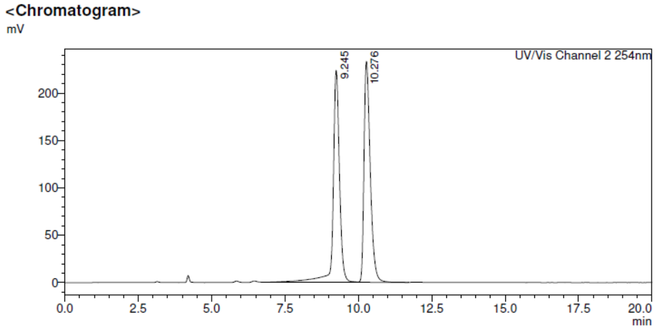

<Peak Table>
UV/Vis Channel 2 254nm

Peak#	Ret. Time	Area	Area%
1	9.245	3385379	49.595
2	10.276	3440605	50.405
Total		6825984	100.000

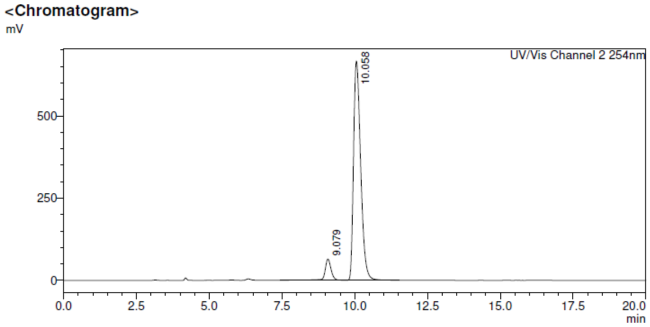

<Peak Table>
UV/Vis Channel 2 254nm

Peak#	Ret. Time	Area	Area%
1	9.079	933263	7.600
2	10.058	11346803	92.400
Total		12280066	100.000

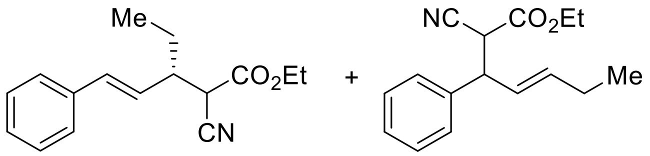

5e + 6e

Ethyl (3*S*,*E*)-2-cyano-3-ethyl-5-phenylpent-4-enoate (5e) and ethyl (*E*)-2-cyano-3-methyl-6-phenylhex-4-enoate (6e): Prepared by General Method using **Pd-1** at 22 ˚C for 20 h. The material was purified by flash silica gel chromatography (100% hexanes to 90:10 hexanes:EtOAc) to yield (60:40 **5e**:**6e**) as a colorless oil (28.8 mg, 0.112 mmol, 56.0 % yield). **5e** (60:40 dr): **^{1}H NMR** (500 MHz, $CDCl_3$) *δ* 7.35–7.22 (5H, m), 6.52 (0.4H, d, *J* = 15.8 Hz), 6.48 (0.6H, d, *J* = 15.7 Hz), 6.04 (0.6H, dd, *J* = 15.7, 9.6 Hz), 5.97 (0.4H, dd, *J* = 15.7, 9.4 Hz), 4.26–4.11 (2.4H, m), 3.64 (0.6 H, d, *J* = 4.6 Hz), 3.53 (0.4H, d, *J* = 6.5 Hz), 2.84–2.78 (1H, m), 2.10–2.02 (0.6H, m), 1.80–1.54 (1.4H, m), 1.26 (1,2H, t, *J* = 7.1 Hz), 1.21 (1.8H, t, *J* = 7.1 Hz), 0.97 (1.8H, t, *J* = 7.3 Hz), 0.94 (1.2H, t, *J* = 7.4 Hz); **6e**: **^{1}H NMR** (500 MHz, $CDCl_3$) *δ* 7.35–7.22 (5H, m), 5.76–5.69 (2H, m), 4.26–4.11 (4H, m), 4.00–3.94 (1H, m), 3.78–3.74 (1H, m), 1.31–1.15 (3H, m), 1.00–0.93 (3H, m); **5e** and **6e** mixture: **^{13}C NMR** (125 MHz, $CDCl_3$) *δ* 165.6, 165.4, 165.1, 139.3, 138.7, 137.5, 136.4, 136.3, 134.3, 134.0, 129.0, 128.9, 127.8, 127.6, 126.7, 126.5, 125.3, 115.7, 115.6, 115.3, 62.8, 62.7, 49.2, 49.1, 46.0, 45.9, 45.2, 44.8, 43.7, 43.3, 26.4, 25.6, 25.5, 14.1, 14.0, 13.4, 11.8, 11.6; **5e** and **6e** mixture: **IR** (neat, cm^{-1}) 2965 (m), 2932 (m), 2875 (w), 1740 (s), 1450 (m), 1368 (m), 1247 (s), 1189 (s), 1029 (s), 967 (s), 748 (s), 694 (s); **HRMS** (ESI^{+}) $[M+H]^{+}$ calc'd for $C_{16}H_{20}O_2N$: 258.1489, found: 258.1490.

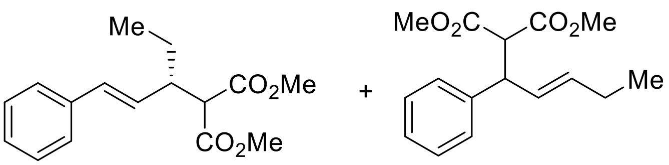

5f + 6f

Dimethyl (*S*,*E*)-2-(1-phenylpent-1-en-3-yl)malonate (5f) and dimethyl (*E*)-2-(5-phenylpent-3-en-2-yl)malonate (6f): Prepared by The General Method using **Pd-1** at 22 ˚C for 20 h. The material was purified by flash silica gel chromatography (100% hexanes to 90:10 hexanes:EtOAc) to yield (57:43 **5f**:**6f**) as a colorless oil (27.1 mg, 0.0980 mmol, 49.0 % yield). **5f**: **^{1}H NMR** (500 MHz, $CDCl_3$) δ 7.38–7.23 (5H, m), 6.48 (1H, d, J = 15.8 Hz), 6.03 (1H, dd, J = 15.8, 9.6 H), 3.77 (3H, s), 3.51–3.49 (1H, m), 2.92–2.86 (1H, m), 1.65–1.38 (2H, m), 1.01–0.91 (3H, m). **6f**: **^{1}H NMR** (500 MHz, $CDCl_3$) δ 7.38–7.23 (5H, m), 5.64–5.58 (2H, m), 4.09–4.04 (1H, m), 3.85 (1H, d, J = 11.1 Hz), 3.75 (3H, s), 3.51 (3H, s), 2.02–1.98 (2H, m), 0.98–0.92 (3H, m); **HRMS** (ESI$^+$) $[M+H]^+$ calc'd for $C_{16}H_{20}O_4$: 277.1434, found: 277.1437. Spectral data matched those previously reported.[8]

V. Additonal Experiments

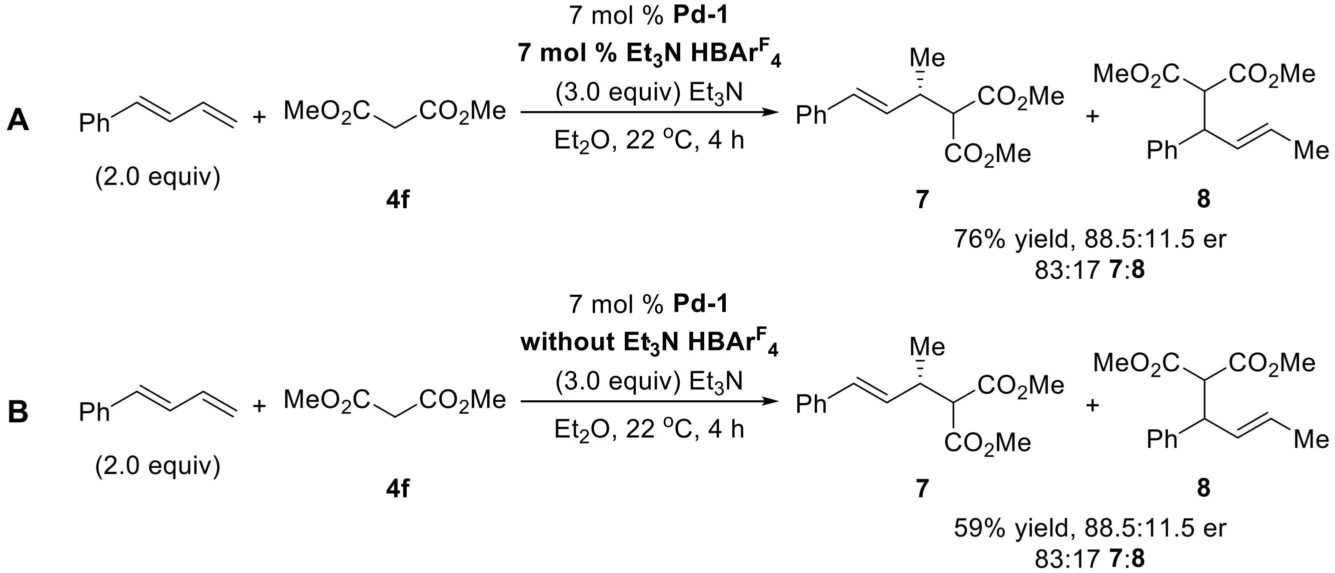

Dimethyl (*S*,*E*)-2-(4-phenylbut-3-en-2-yl)malonate (7): Prepared by The General Method using **Pd-1** with dimethyl malonate (26.2 mg, 0.200 mmol, 1.0 equiv) at 22 ˚C for 4 h. The material was purified by flash silica gel chromatography (100% hexanes to 90:10 hexanes:EtOAc) to yield (83:17 **8**:**9**) as a colorless oil (40.0 mg, 0.152 mmol, 76.2% yield). **7**: ^{1}**H NMR** (500 MHz, $CDCl_3$) δ 7.31 (2H, d, J = 7.2 Hz), 7.27 (2H, dd, J = 7.6, 7.6 Hz), 7.22–7.16 (1H, m), 6.43 (1H, d, J = 15.8 Hz), 6.10 (1H, dd, J = 15.8, 8.5 Hz), 3.73 (3H, s), 3.65 (3H, s), 3.38 (1H, d, J = 8.9 Hz), 3.15–3.06 (1H, m), 1.17 (3H, d, J = 6.8 Hz); **8**: ^{1}**H NMR** (500 MHz, $CDCl_3$) δ 7.28–7.18 (5H, m), 5.63–5.50 (2H, m), 4.05–3.99 (1H, m), 3.80 (1H, d, J = 11.0 Hz), 3.71 (s, 3H), 3.46 (s, 3H), 1.61 (d, J = 5.3 Hz, 3H); **HRMS** (ESI^+) $[M+H]^+$ calc'd for $C_{15}H_{19}O_4$: 263.1278, found: 263.1282. Spectral data matched those previously reported.[9]
$[\alpha]_D^{27}$ = −15.8 (c = 1.0, $CHCl_3$) for a sample of 88.5:11.5 er.
HPLC: Column: Lux amylose-1 (3 μm, 4.60 mm X 250 mm). Mobile phase: 97:3 *i*-PrOH: Hexanes, 0.8 mL/min. Detection wavelength: 254 nm. **A-2e**: Er = 88.5:11.5; **B-2e**: Er = 88.5:11.5.

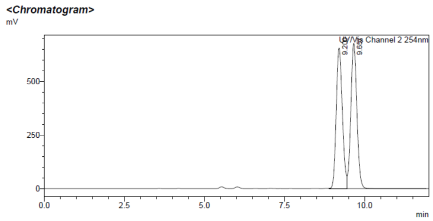

<Peak Table>
UV/Vis Channel 2 254nm

Peak#	Ret. Time	Area	Area%
1	9.200	8730645	49.509
2	9.657	8903724	50.491
Total		17634369	100.000

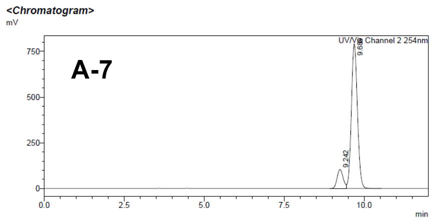

<Peak Table>
UV/Vis Channel 2 254nm

Peak#	Ret. Time	Area	Area%
1	9.242	1361748	11.551
2	9.689	10426924	88.449
Total		11788671	100.000

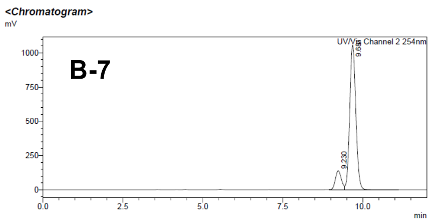

<Peak Table>
UV/Vis Channel 2 254nm

Peak#	Ret. Time	Area	Area%
1	9.230	1813788	11.447
2	9.681	14031383	88.553
Total		15845170	100.000

VI. Product Derivatizations

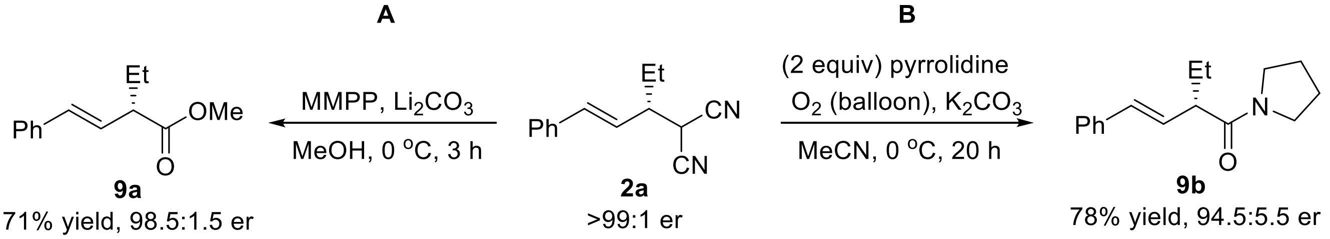

Application A: Methyl (*S*,*E*)-2-ethyl-4-phenylbut-3-enoate (9a): Compound **9a** was prepared according to a previously established method.[10] To a 2-dram vial equipped with a magnetic stirring rod were added **2a** (42.1 mg, 0.200 mmol, 1.00 equiv) and Li_2CO_3 (22.0 mg, 0.300 mmol, 1.50 equiv). The mixture was suspended in MeOH (1.3 mL) and allowed to cool to 0 °C. To this mixture was added magnesium monoperoxyphthalate (MMPP, 80% tech grade, 72.5 mg, 0.150 mmol, 0.750 equiv). After allowing to stir vigorously at 0 °C for 3 h, the suspension was filtered through a pad of celite followed by a plug of silica gel (eluting with excess Et_2O in both cases) and then the filtrate was concentrated *in vacuo*. The material was purified by flash silica gel chromatography (100% hexanes to 95:5 hexanes:EtOAc) to yield **9a** as a colorless oil (29.0 mg, 0.142 mmol, 71.0% yield). **^{1}H NMR** (500 MHz, $CDCl_3$) δ 7.35 (2H, d, J = 7.4 Hz), 7.29 (2H, dd, J = 7.6, 7.6 Hz), 7.21 (1H, t, J = 7.3 Hz), 6.45 (1H, d, J = 15.9 Hz), 6.17 (1H, dd, J = 15.9, 9.0 Hz), 3.69 (3H, s), 3.07 (1H, ddd, J = 7.4, 7.4, 7.4 Hz), 1.90–1.61 (1H, m), 1.70–1.60 (1H, m), 0.93 (3H, t, J = 7.4 Hz); **^{13}C NMR** (125 MHz, $CDCl_3$) δ 174.6, 136.9, 132.4, 128.6, 127.6, 127.6, 126.4, 51.9, 51.3, 26.0, 11.8; **IR** (neat, cm^{-1}) 3026 (w), 2964 (m), 2932 (m), 2875 (w), 1732 (s), 1449 (m), 1160 (s), 965 (s), 742 (s), 692 (s); **HRMS** (ESI$^+$) [M+H]$^+$ calc'd for $C_{13}H_{16}O_2$: 205.1223 found: 205.1226. **$[\alpha]_D^{27}$** = −14.8 (*c* = 1.0, $CHCl_3$) for a sample of 98.5:1.5 er.

HPLC: Column: Lux cellulose-1 (3 μm, 4.60 mm X 250 mm). Mobile phase: 10:90 *i*-PrOH:Hexanes, 1.0 mL/min. Detection wavelength: 254 nm. Er = 98.5:1.5.

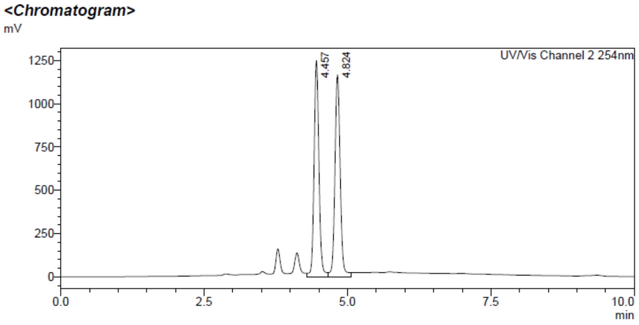

<Peak Table>

UV/Vis Channel 2 254nm

Peak#	Ret. Time	Area	Area%
1	4.457	7415141	49.593
2	4.824	7536925	50.407
Total		14952066	100.000

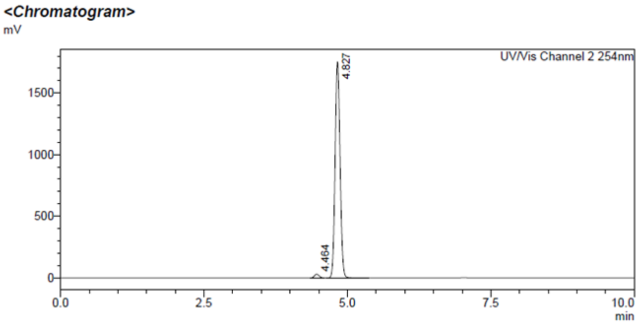

<Peak Table>

UV/Vis Channel 2 254nm

Peak#	Ret. Time	Area	Area%
1	4.464	164482	1.522
2	4.827	10640218	98.478
Total		10804700	100.000

Application B: (*S*,*E*)-2-Ethyl-4-phenyl-1-(pyrrolidin-1-yl)but-3-en-1-one (9b): Compound **9b** was prepared according to a previously established method.[11] To a 2-dram vial equipped with a magnetic stirring rod was added a solution of **2a** (42.1 mg, 0.200 mmol, 1.00 equiv) in CH_3CN (2.0 mL). Then, oxygen (balloon) was bubbled through the solution for 5 min at 0 ºC. Pyrrolidne (28.5 mg, 0.400 mmol, 2.00 equiv) and K_2CO_3 (55.3 mg, 0.400 mmol, 2.00 equiv) were added in one-portion sequentially. The atmosphere was refreshed under oxygen (balloon) at 0 ºC. After allowing the mixture to stir for 20 h at 0 ºC, $CHCl_3$ (2 mL) was added and the precipitate filtered through a short silica gel column and then the filtrate was concentrated *in vacuo*. The material was purified by flash silica gel chromatography (90:10 hexanes:EtOAc to 50:50 hexanes:EtOAc) to yield **9b** as a colorless oil (38.0 mg, 0.156 mmol, 78.0% yield). **^{1}H NMR** (500 MHz, $CDCl_3$) δ 7.34 (2H, d, J = 7.3 Hz), 7.27 (2H, dd, J = 7.6, 7.6 Hz), 7.19 (1H, t, J = 7.3 Hz), 6.40 (1H, d, J = 15.9 Hz), 6.21 (1H, dd, J = 15.9, 9.0 Hz), 3.56–3.43 (4H, m), 3.14–3.08 (1H, m), 1.97–1.77 (5H, m), 1.67–1.58 (1H, m), 0.91 (3H, t, J = 7.4 Hz); **^{13}C NMR** (125 MHz, $CDCl_3$) δ 171.9, 137.0, 131.5, 129.1, 128.5, 127.4, 126.3, 50.6, 46.4, 45.9, 26.1, 25.9, 24.3, 12.0; **IR** (neat, cm^{-1}) 2964 (s), 2872 (s), 1624 (s), 1427 (s), 968 (m), 910 (m), 727 (s), 692 (s); **HRMS** (ESI$^+$) $[M+H]^+$ calc'd for $C_{16}H_{21}ON$: 244.1696, found: 244.1701.

$[\alpha]_D^{27}$ = –14.9 (c = 1.0, $CHCl_3$) for a sample of 94.5:5.5 er.

HPLC: Column: Lux cellulose-1 (3 μm, 4.60 mm X 250 mm). Mobile phase: 10:90 *i*-PrOH:Hexanes, 1.0 mL/min. Detection wavelength: 254 nm. Er = 94.5:5.5.

<Chromatogram>

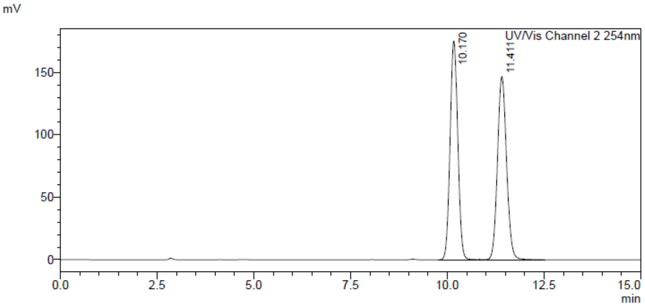

<Peak Table>

UV/Vis Channel 2 254nm

Peak#	Ret. Time	Area	Area%
1	10.170	2416247	50.090
2	11.411	2407563	49.910
Total		4823810	100.000

<Chromatogram>

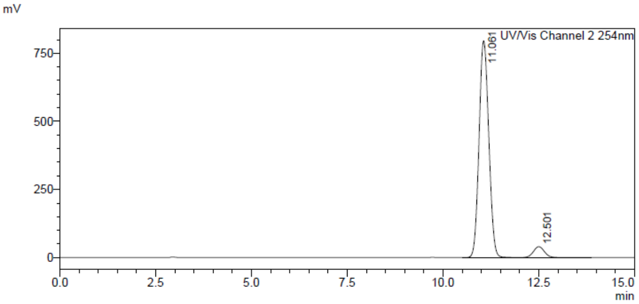

<Peak Table>

UV/Vis Channel 2 254nm

Peak#	Ret. Time	Area	Area%
1	11.061	14059266	94.524
2	12.501	814479	5.476
Total		14873745	100.000

VII. Reaction Outcome Dependence on Diene Stereochemistry

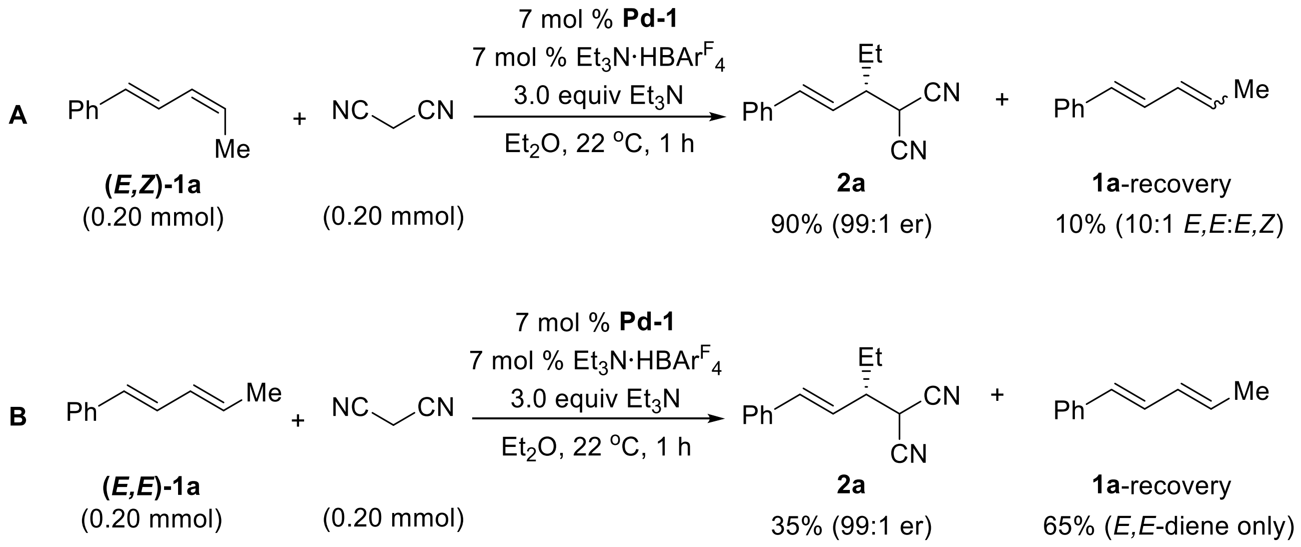

Reactions were carried as described in The General Method but with 0.20 mmol (1.0 equiv) **1a** at 22 ºC for 1 h. Products **2a** and **1a-**recovery were then purified by flash silica gel chromatography (100% hexanes to 80:20 hexanes:EtOAc).

HPLC: Column: Lux amylose-2 (3 μm, 4.60 mm X 250 mm). Mobile phase: 90:10 *i*-PrOH: Hexanes, 1.0 mL/min. Detection wavelength: 254 nm. **A-2e**: Er = 99:1; **B-2e**: Er = 99:1.

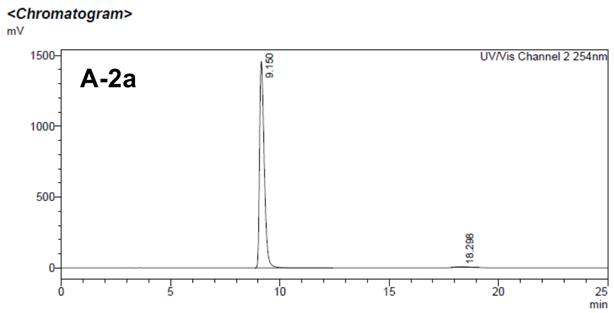

<Peak Table>
UV/Vis Channel 2 254nm

Peak#	Ret. Time	Area	Area%
1	9.150	21519087	98.926
2	18.298	233553	1.074
Total		21752640	100.000

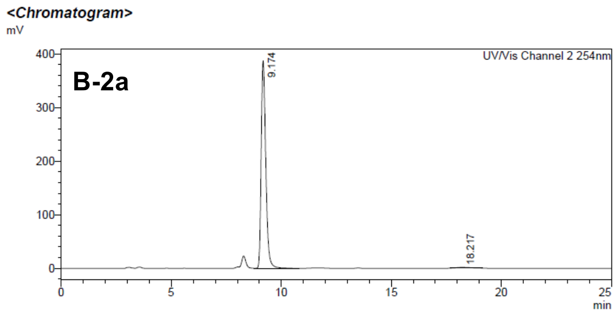

<Peak Table>
UV/Vis Channel 2 254nm

Peak#	Ret. Time	Area	Area%
1	9.174	5610376	98.870
2	18.217	64095	1.130
Total		5674471	100.000

VIII. Deuterium Labelling Studies

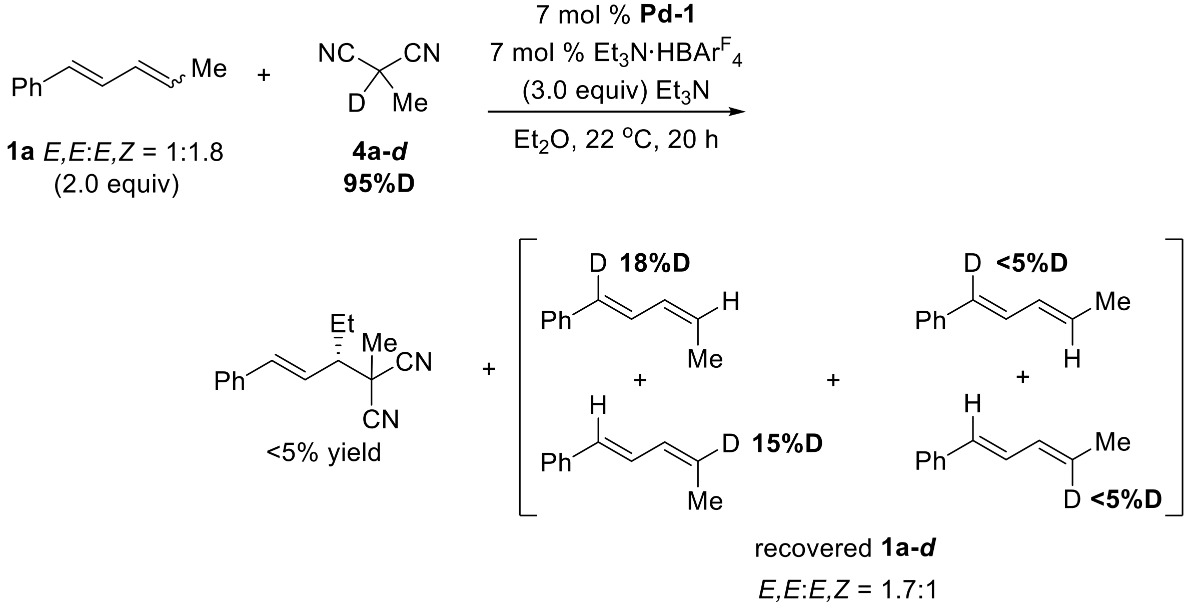

D-Methylmalononitrile was prepared by a known method.[12] Reactions were carried as described in The General Method at 22 ºC. Recovered **1a-*d*** was then purified by flash silica gel chromatography (100% hexanes to 90:10 hexanes:EtOAc).

IV. References

(1) M. Brookhart, B. Grant and F. Volpe, A, *Organometallics* 1992, **11**, 3920–3922.

(2) S. Park and S. J. Malcolmson, *ACS Catal*. 2018, **8**, 8468–8476.

(3) N. J. Adamson, E. Hull, and S. J. Malcolmson, *J. Am. Chem. Soc.* 2017, **139**, 7180−7183; N. J. Adamson, K. C. E. Wibur and S. J. Malcolmson, *J. Am. Chem. Soc.* 2018, **140**, 2761−2764.

(4) D.-Q. Shi and R.-Y. Chen, *Chinese Chemical Letters* 1999, **10**, 911–914.

(5) M. K. Ghorai, R. Talukdar and D. P. Tiwari, *Org. Lett*. 2014, **16**, 2204–2207; G. Dickmeiss, K. L. Jensen, D. Worgull, P. T. Franke and K. A. Jørgensen, *Angew. Chem., Int. Ed*. 2011, **50**, 1580–1583.

(6) M. Tiecco, L. Testaferri, M. Tingoli and D. Bartoli, *Tetrahedron* 1989, **45**, 6819–6832; A. Llevot, B. Monney, A. Sehlinger, S. Behrens and M. A. R. Meier, *Chem. Commun*. 2017, **53**, 5175–5178; X. Zhang, X. Jia, L. Fang, N. Liu, J. Wang and X. Fan, *Org. Lett*. 2011, **13**, 5024–5027.

(7) Y. Wang, Y.-N. Xu, G.-S. Fang, H.-J. Kang, Y. Gu and S.-K. Tian, *Org. Biomol. Chem*. 2015, **13**, 5367–5371.

(8) D. Laurenti, M. Feuerstein, G. Pèpe, H. Doucet, and M. Santelli, *J. Org. Chem*. 2001, **66**, 1633–1637.

(9) K. Balaraman, R. Vasanthan and V. Kesavan, *Tetrahedron* 2013, **69**, 6162–6169.

(10) S. Förster, O. Tverskoy, G. Helmchen, *Synlett* 2008, 2803–2806.

(11) J. Li, M. J. Lear and Y. Hayashi, *Angew. Chem. Int. Ed*. 2016, **55**, 9060–9064.

(12) B. H. Oh, I. Nakamura and Y. Yamamoto, *Tetrahedron Lett.* 2002, **43**, 9625–9628.

X. NMR Spectra

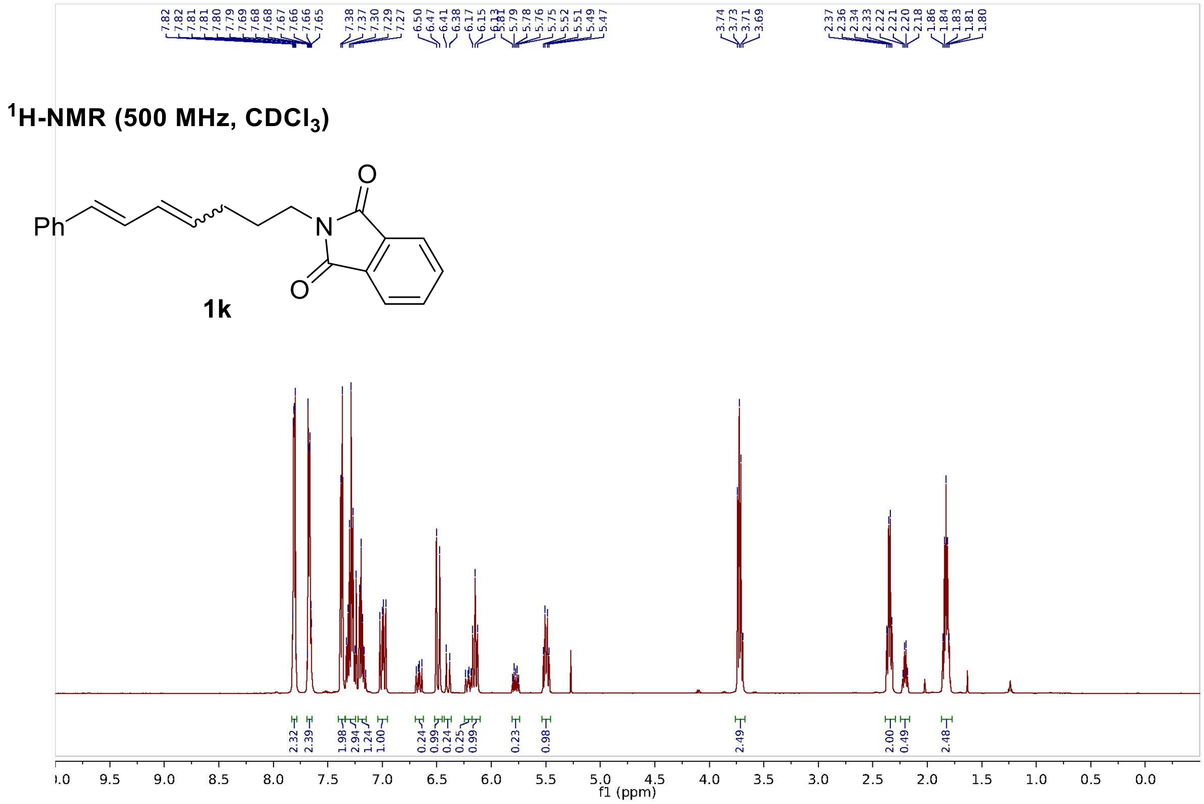

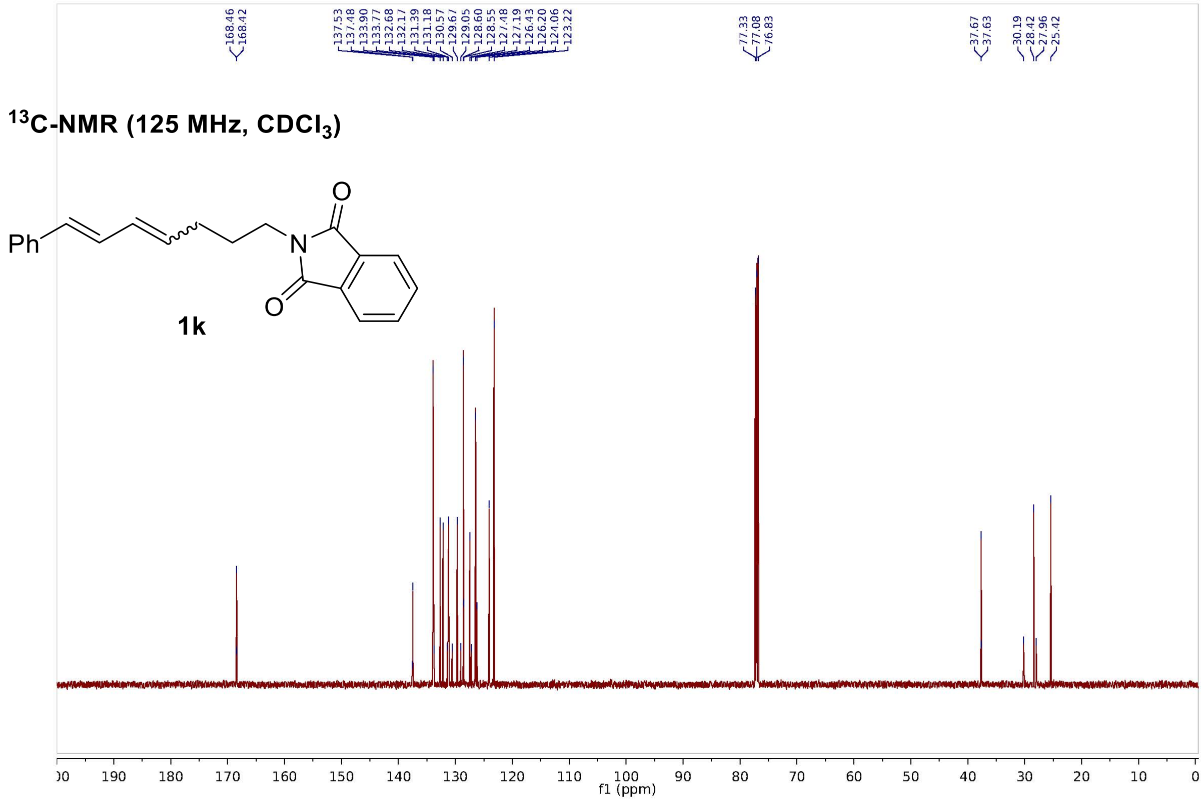

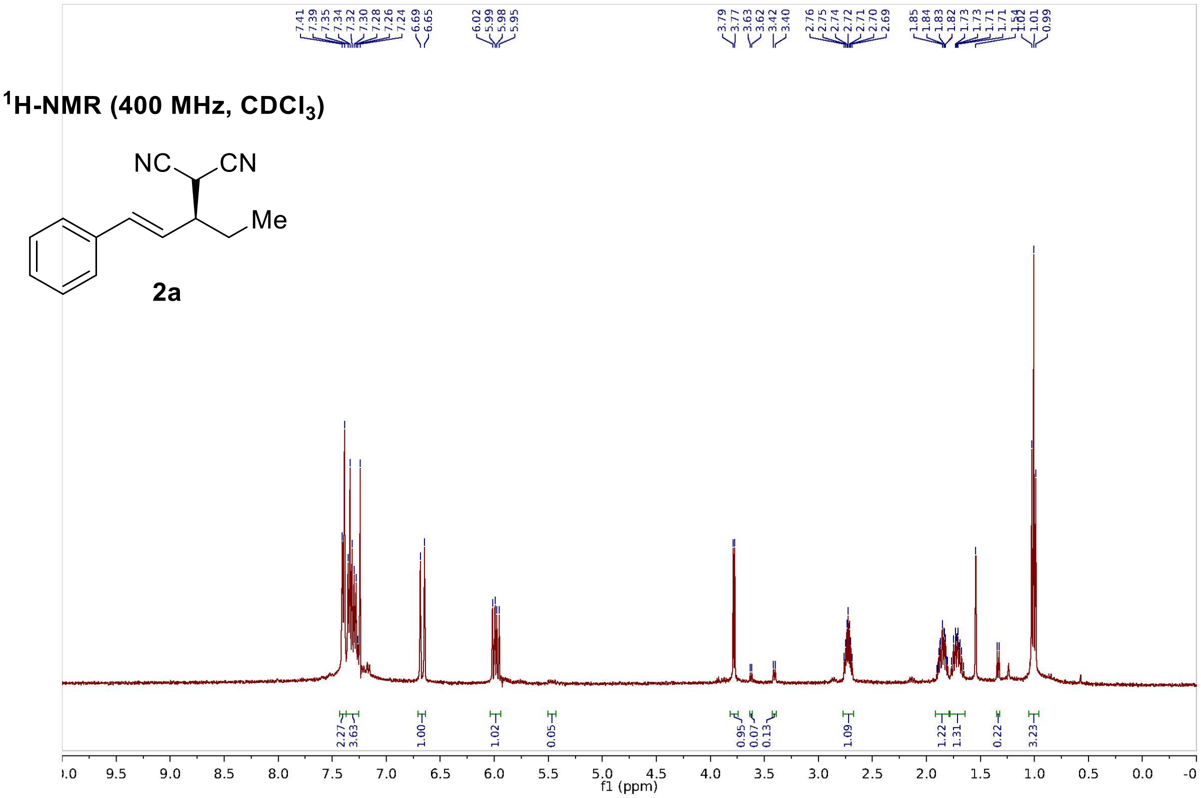

^{1}H-NMR (400 MHz, $CDCl_3$)
NC
CN
Me
2a
7.41 7.39 7.35 7.34 7.32 7.30 7.28 7.26 7.24 6.69 6.65
6.02 5.99 5.98 5.95
3.79 3.77 3.63 3.62 3.42 3.40
2.76 2.75 2.74 2.72 2.71 2.70 2.69
1.85 1.84 1.83 1.82 1.73 1.73 1.71 1.71 1.54 1.02 1.01 0.99
2.27 3.63 1.00 1.02 0.05 0.95 0.07 0.13 1.09 1.22 1.31 0.22 3.23
f1 (ppm)

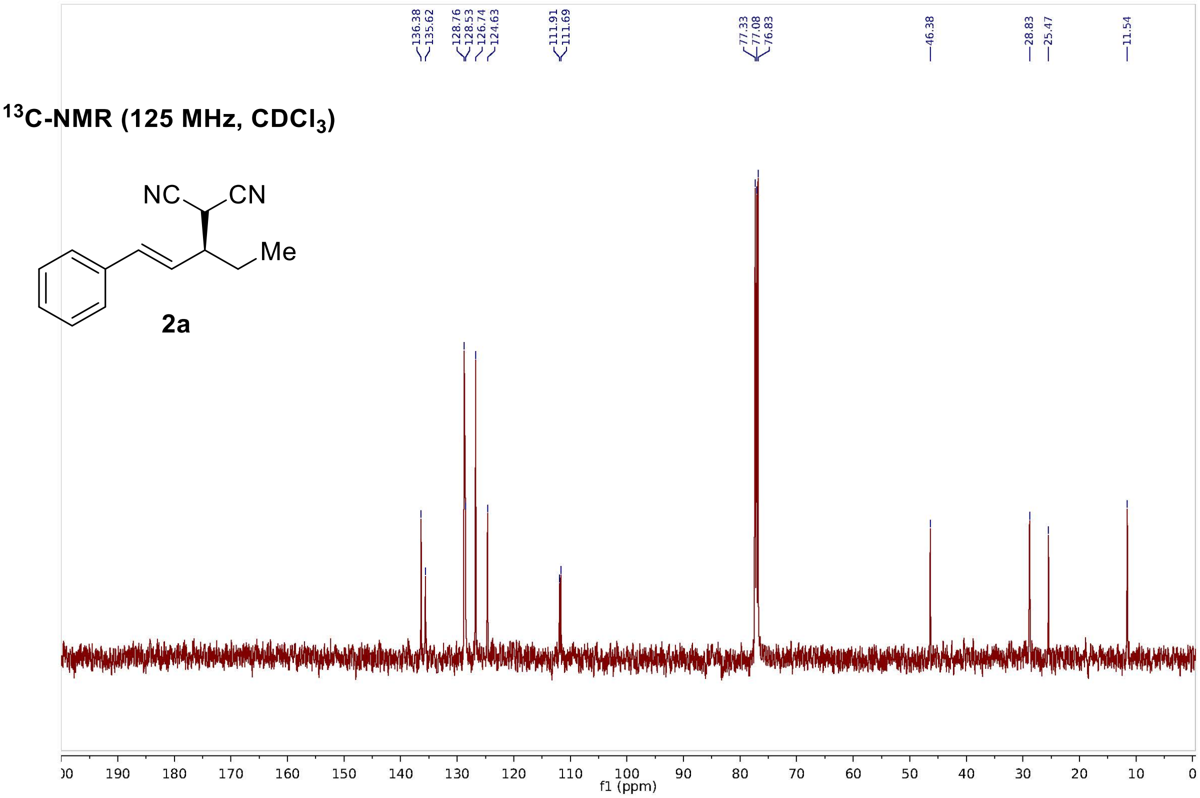

^{13}C-NMR (125 MHz, $CDCl_3$)
NC
CN
Me
2a
136.38 135.62 128.76 128.53 126.74 124.63 111.91 111.69
77.33 77.08 76.83
46.38
28.83 25.47
11.54
f1 (ppm)

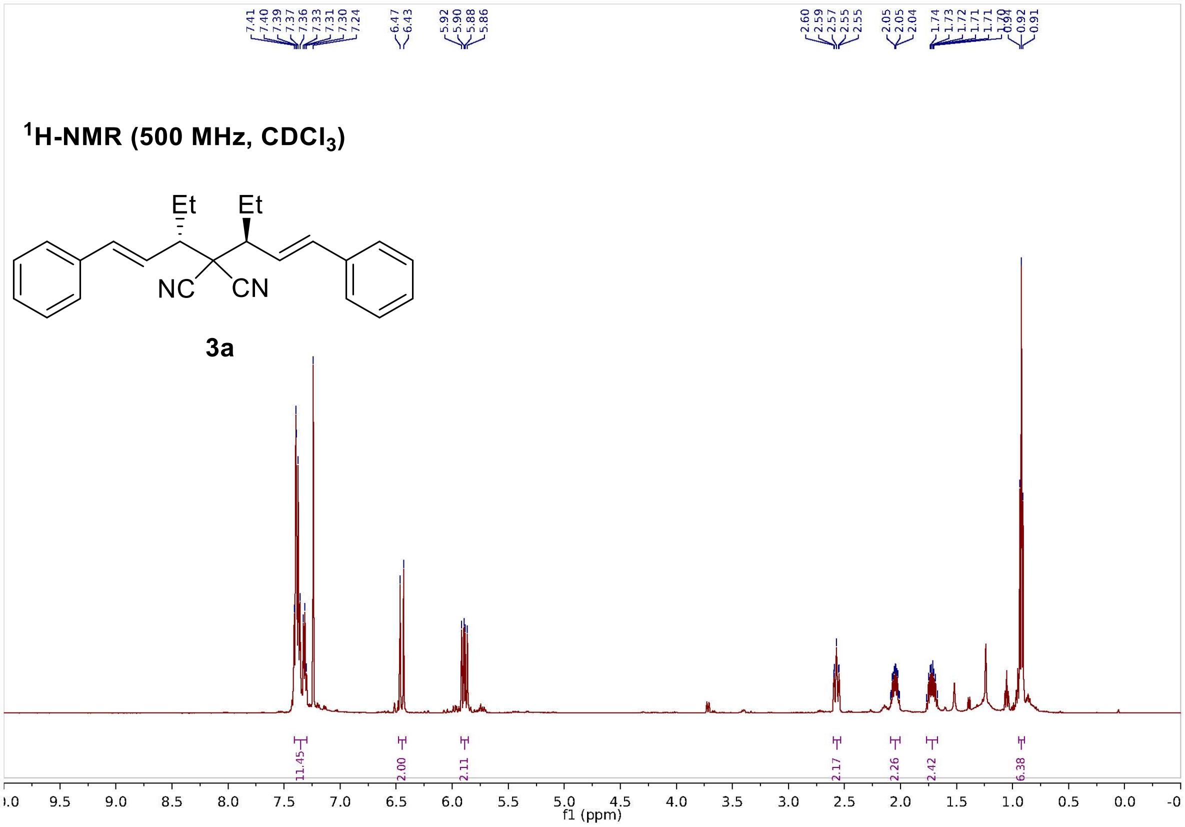
1H-NMR (500 MHz, CDCl3)
Et
Et
NC
CN
3a
7.41
7.40
7.39
7.37
7.36
7.33
7.31
7.30
7.24
6.47
6.43
5.92
5.90
5.88
5.86
2.60
2.59
2.57
2.55
2.55
2.05
2.05
2.04
1.74
1.73
1.72
1.71
1.71
0.92
0.91
11.45
2.00
2.11
2.17
2.26
2.42
6.38
f1 (ppm)

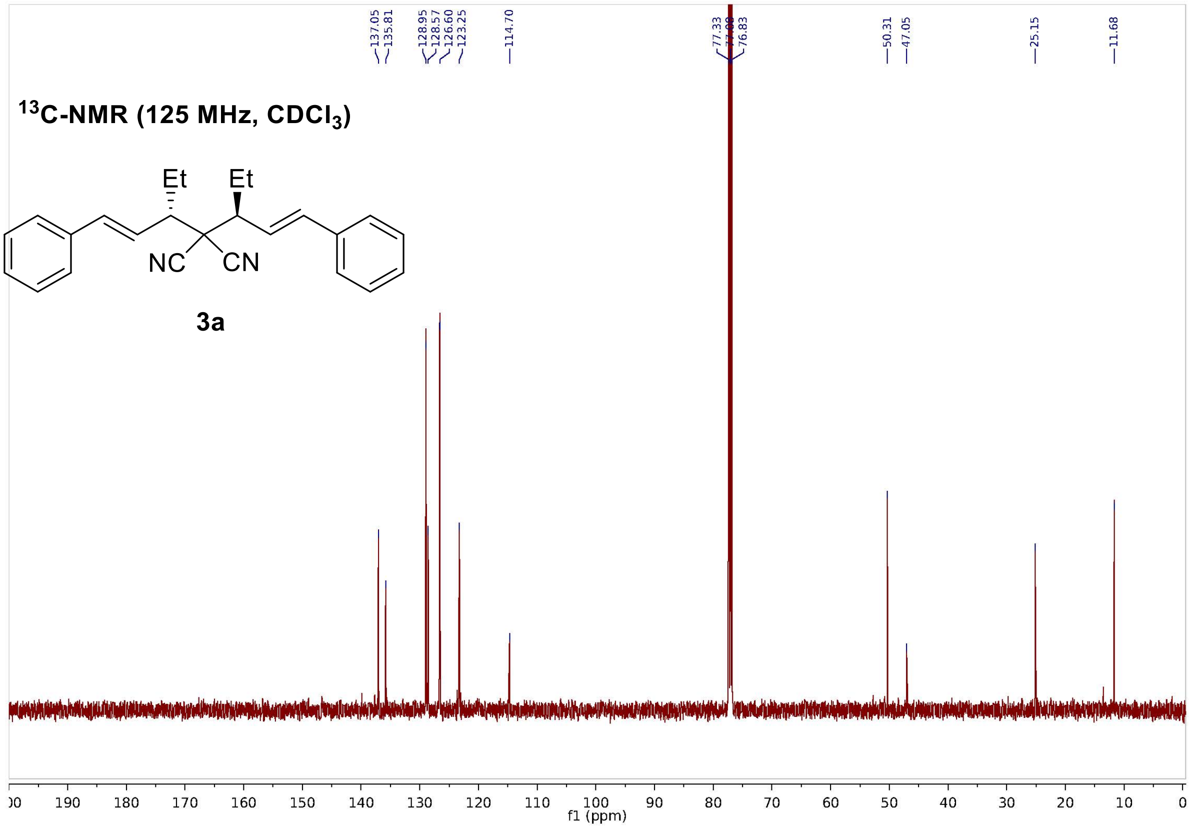
13C-NMR (125 MHz, CDCl3)
Et
Et
NC
CN
3a
137.05
135.81
128.95
128.57
126.60
123.25
114.70
77.33
76.83
50.31
47.05
25.15
11.68
f1 (ppm)

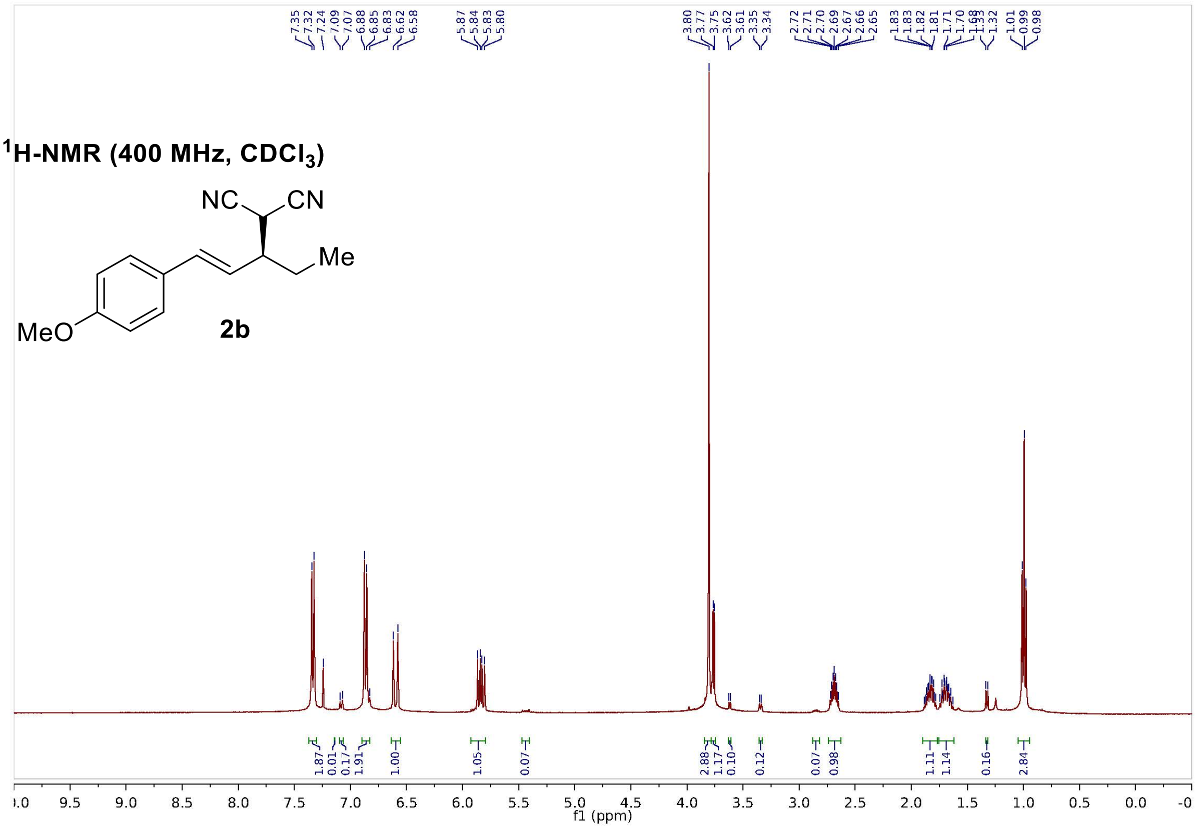
1H-NMR (400 MHz, CDCl3)
NC
CN
Me
MeO
2b
f1 (ppm)

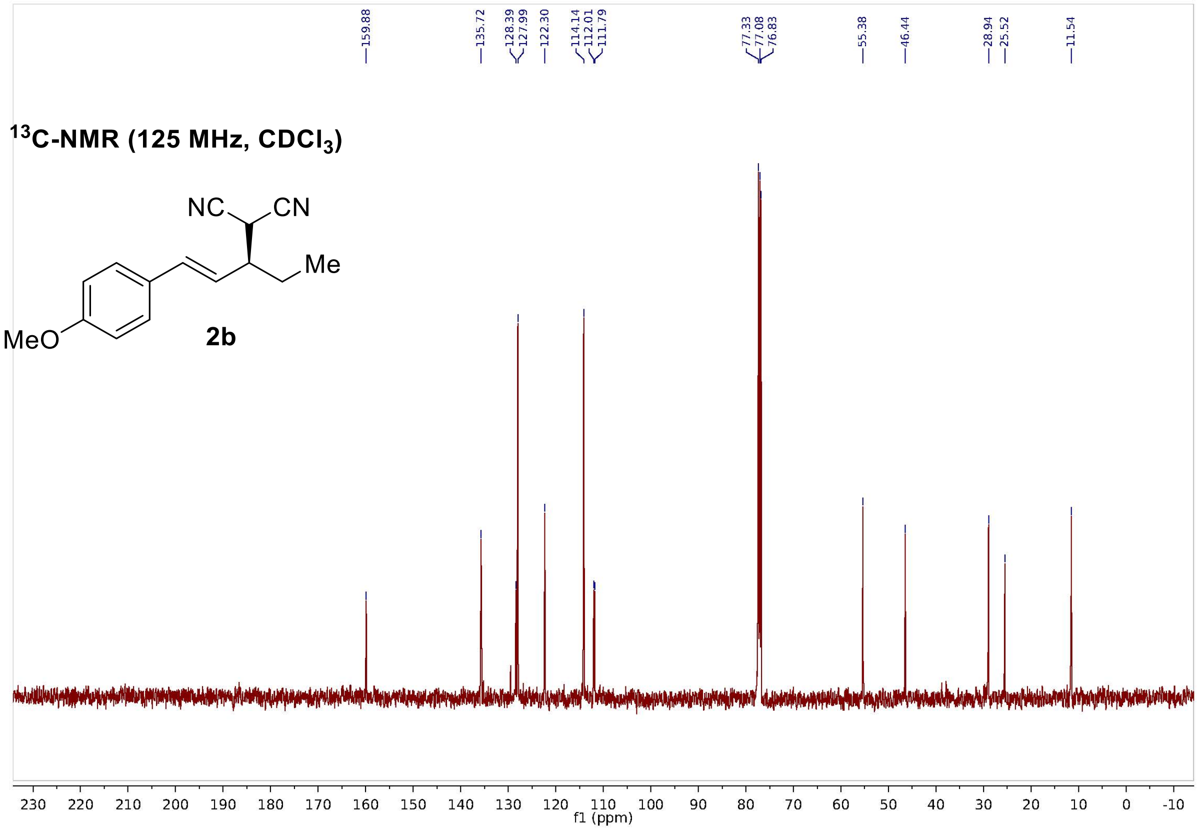
13C-NMR (125 MHz, CDCl3)
NC
CN
Me
MeO
2b
f1 (ppm)

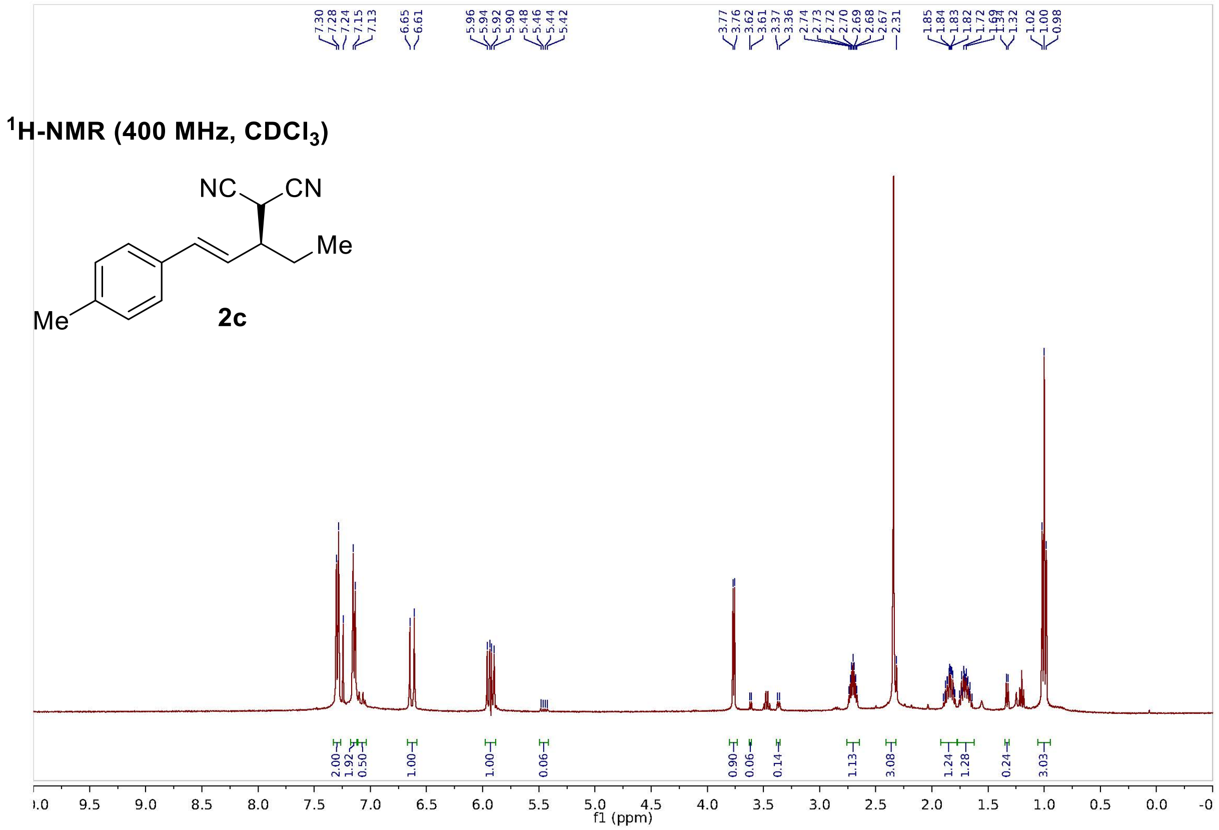

1H-NMR (400 MHz, CDCl3)
NC
CN
Me
Me
2c
f1 (ppm)

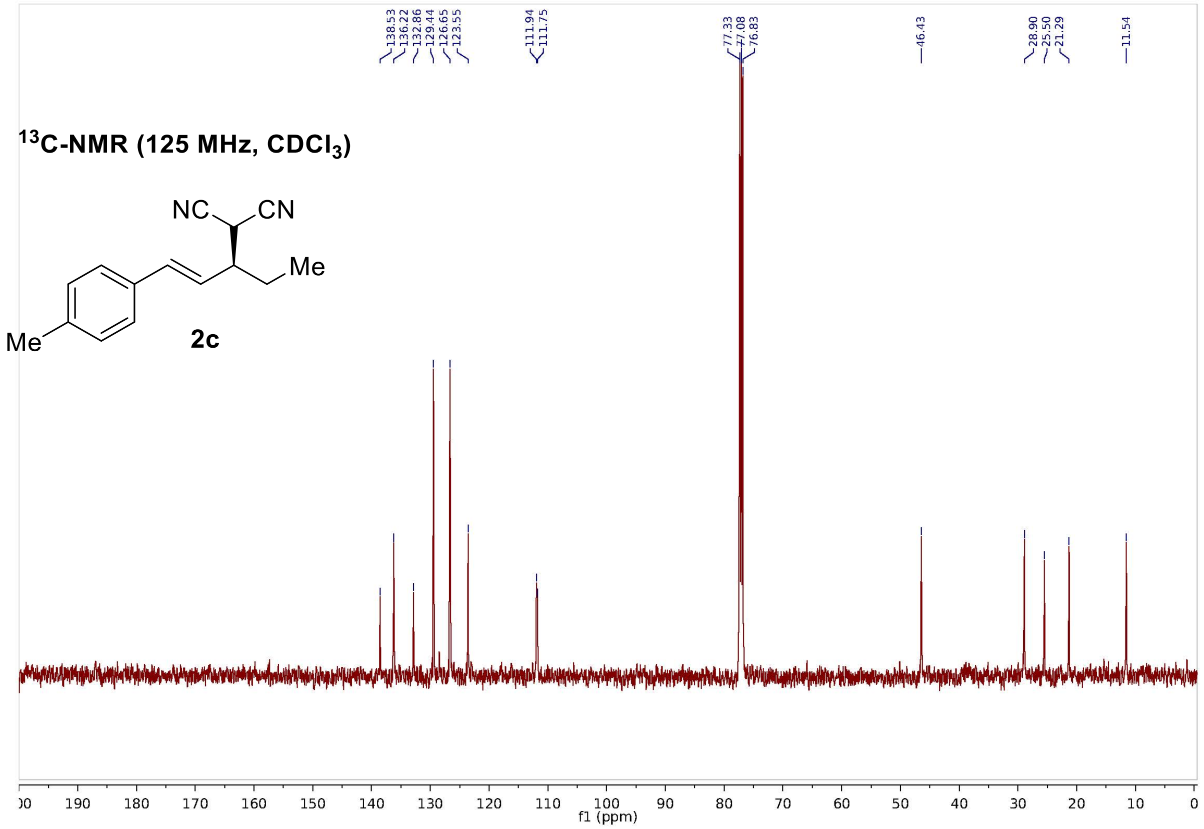

13C-NMR (125 MHz, CDCl3)
NC
CN
Me
Me
2c
f1 (ppm)

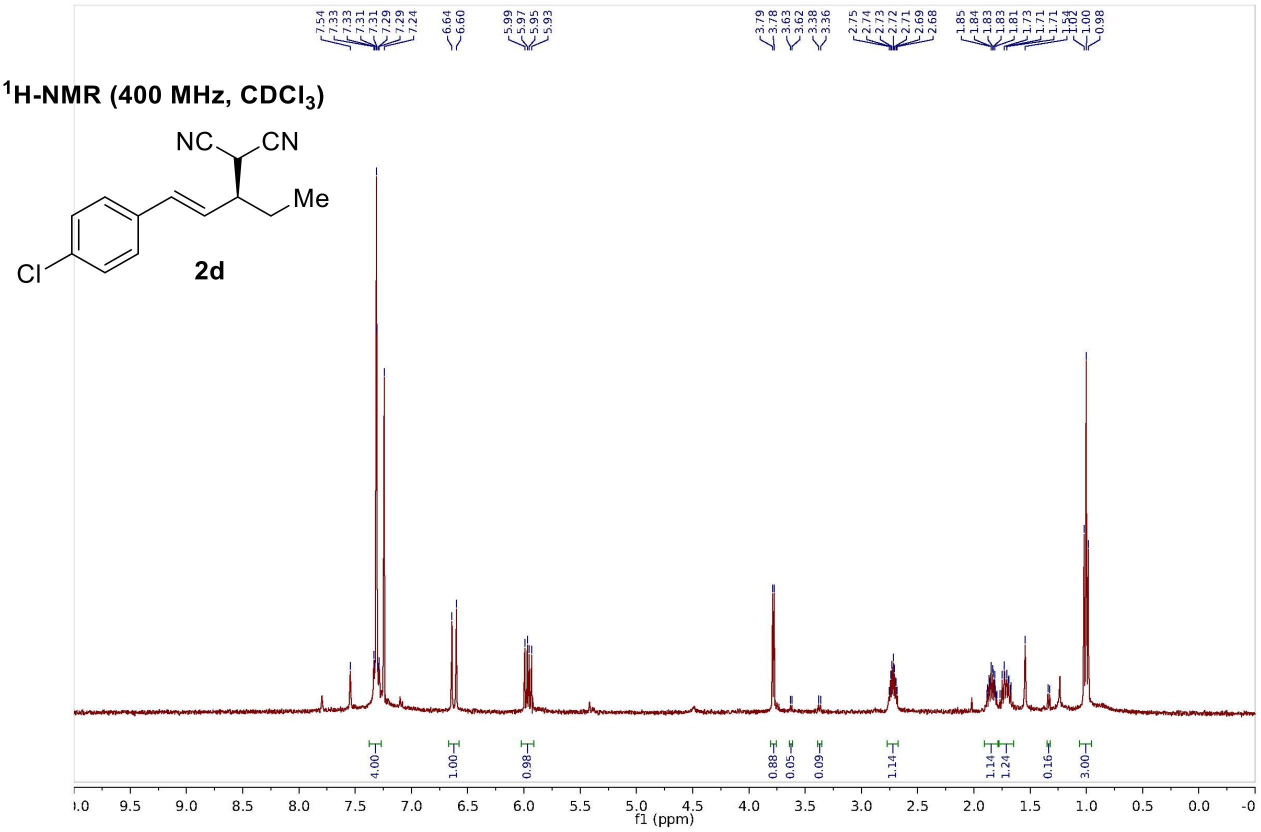
7.54
7.33
7.33
7.31
7.31
7.29
7.29
7.24
6.64
6.60
5.99
5.97
5.95
5.93
3.79
3.78
3.63
3.62
3.38
3.36
2.75
2.74
2.73
2.72
2.71
2.69
2.68
1.85
1.84
1.83
1.83
1.81
1.73
1.71
1.71
1.54
1.02
1.00
0.98
^{1}H-NMR (400 MHz, CDCl$_3$)
NC
CN
Me
Cl
2d
4.00
1.00
0.98
0.88
0.05
0.09
1.14
1.14
1.24
0.16
3.00
f1 (ppm)

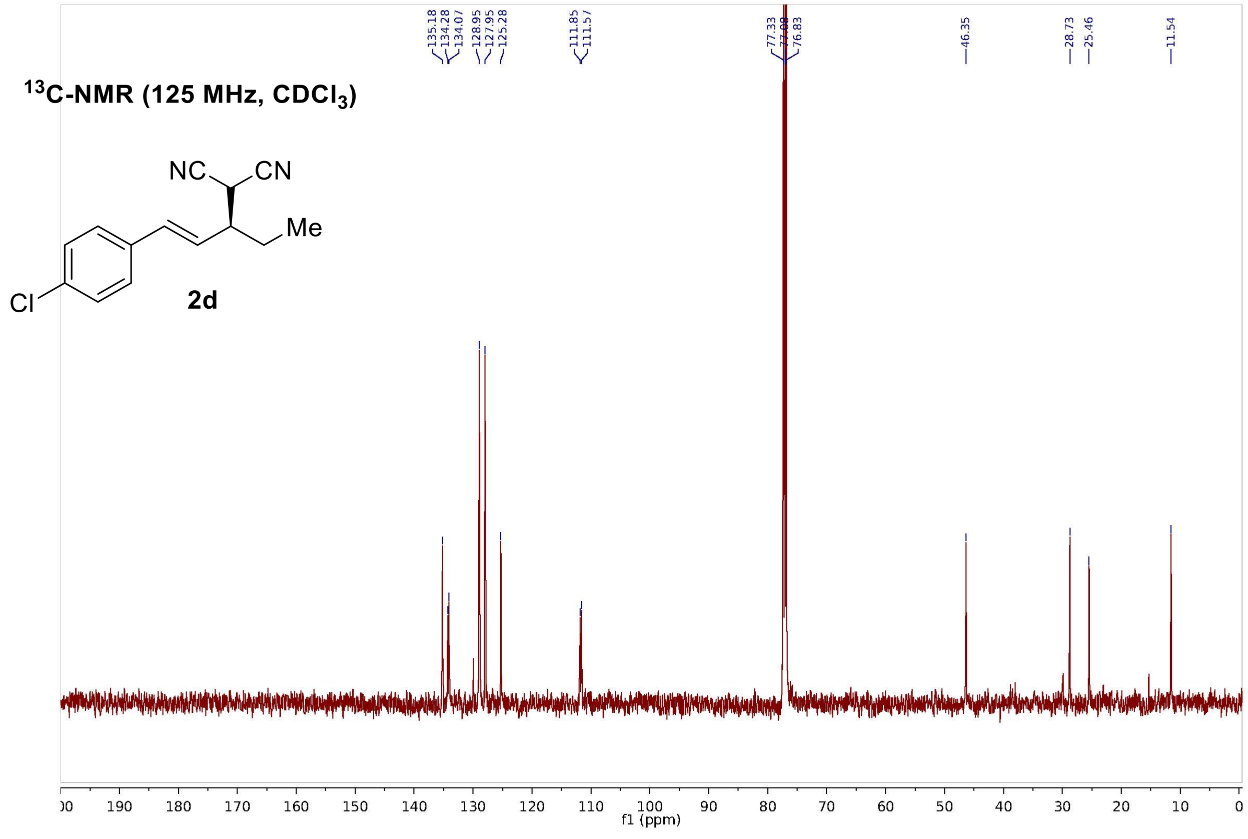
135.18
134.28
134.07
128.95
127.95
125.28
111.85
111.57
77.33
77.08
76.83
46.35
28.73
25.46
11.54
^{13}C-NMR (125 MHz, CDCl$_3$)
NC
CN
Me
Cl
2d
f1 (ppm)

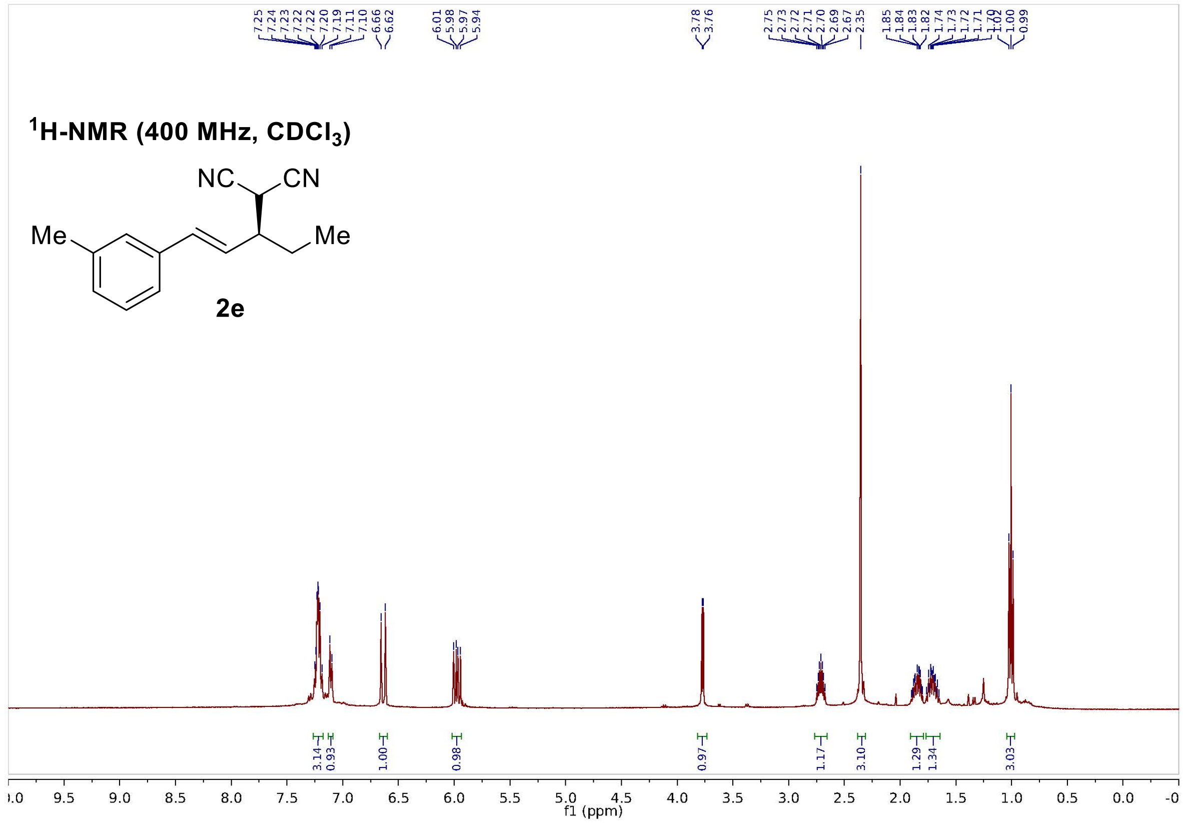

^{1}H-NMR (400 MHz, CDCl3)
NC
CN
Me
Me
2e
f1 (ppm)

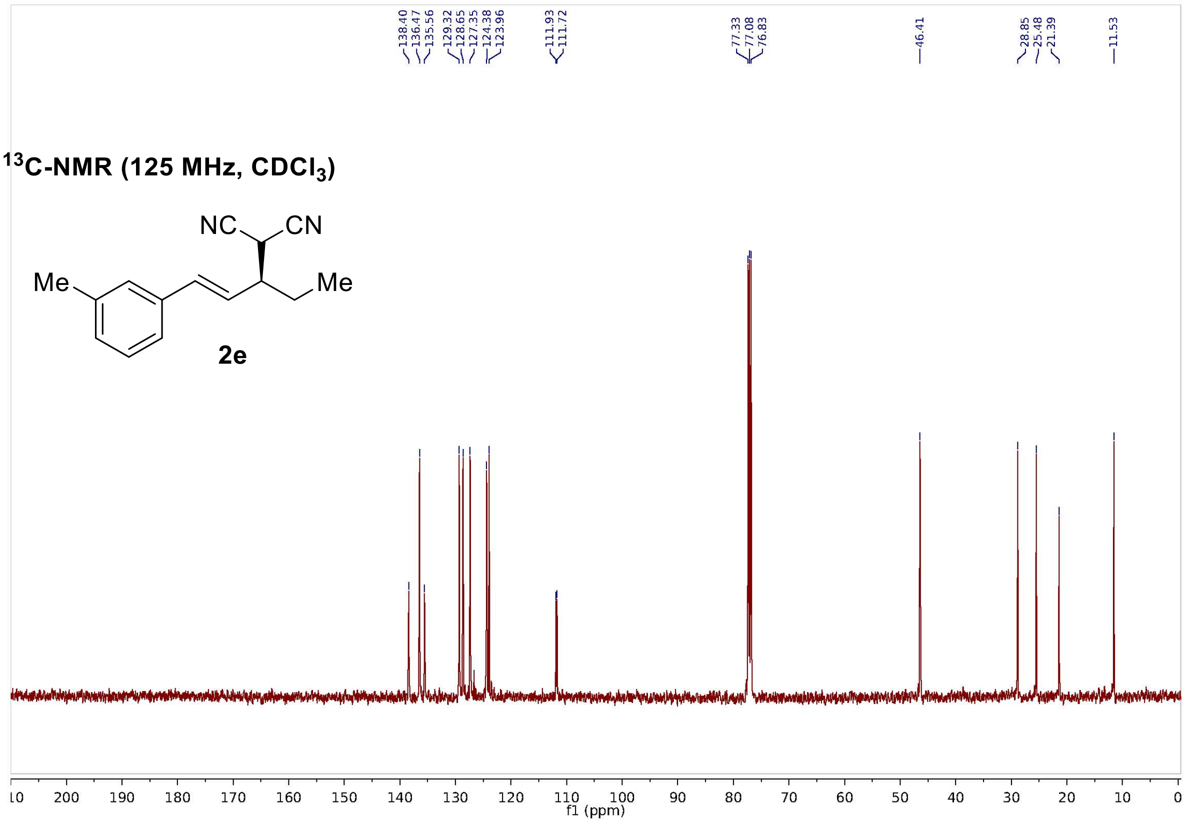

^{13}C-NMR (125 MHz, CDCl3)
NC
CN
Me
Me
2e
f1 (ppm)

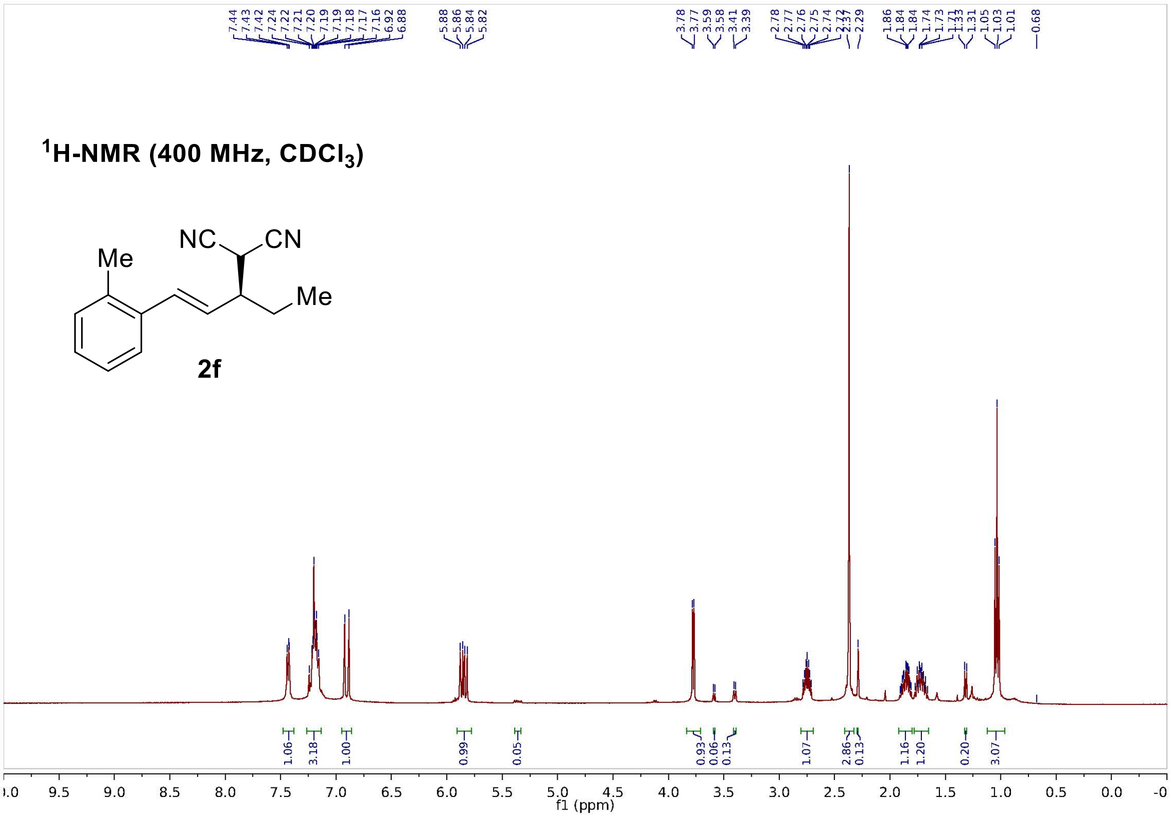
7.44 7.43 7.42 7.24 7.22 7.21 7.20 7.19 7.19 7.18 7.17 7.16 6.92 6.88
5.88 5.86 5.84 5.82
3.78 3.77 3.59 3.58 3.41 3.39
2.78 2.77 2.76 2.75 2.74 2.72 2.37 2.29
1.86 1.84 1.84 1.74 1.73 1.71 1.33 1.31 1.05 1.03 1.01
0.68
1H-NMR (400 MHz, CDCl3)
NC
CN
Me
Me
2f
1.06 3.18 1.00 0.99 0.05 0.93 0.06 0.13 1.07 2.86 0.13 1.16 1.20 0.20 3.07
f1 (ppm)

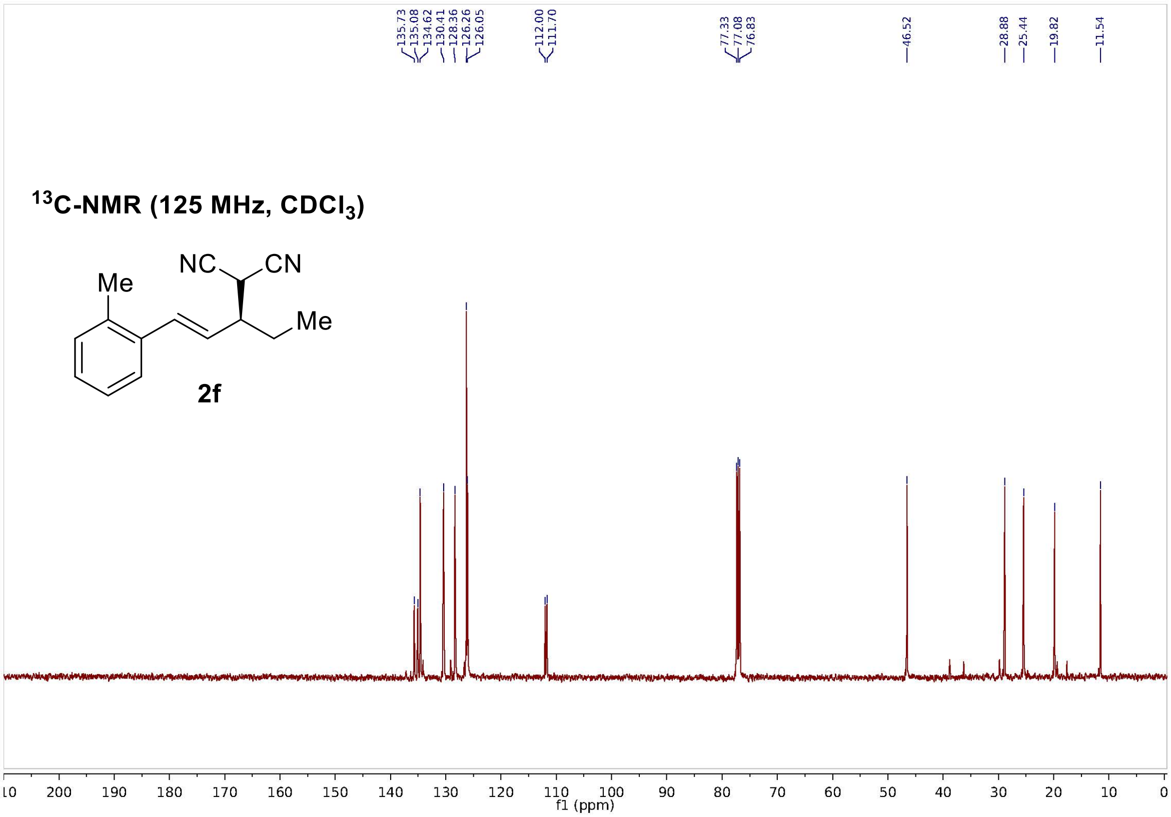
135.73 135.08 134.62 130.41 128.36 126.26 126.05
112.00 111.70
77.33 77.08 76.83
46.52
28.88 25.44 19.82 11.54
13C-NMR (125 MHz, CDCl3)
NC
CN
Me
Me
2f
f1 (ppm)

^{1}H-NMR (400 MHz, $CDCl_3$)

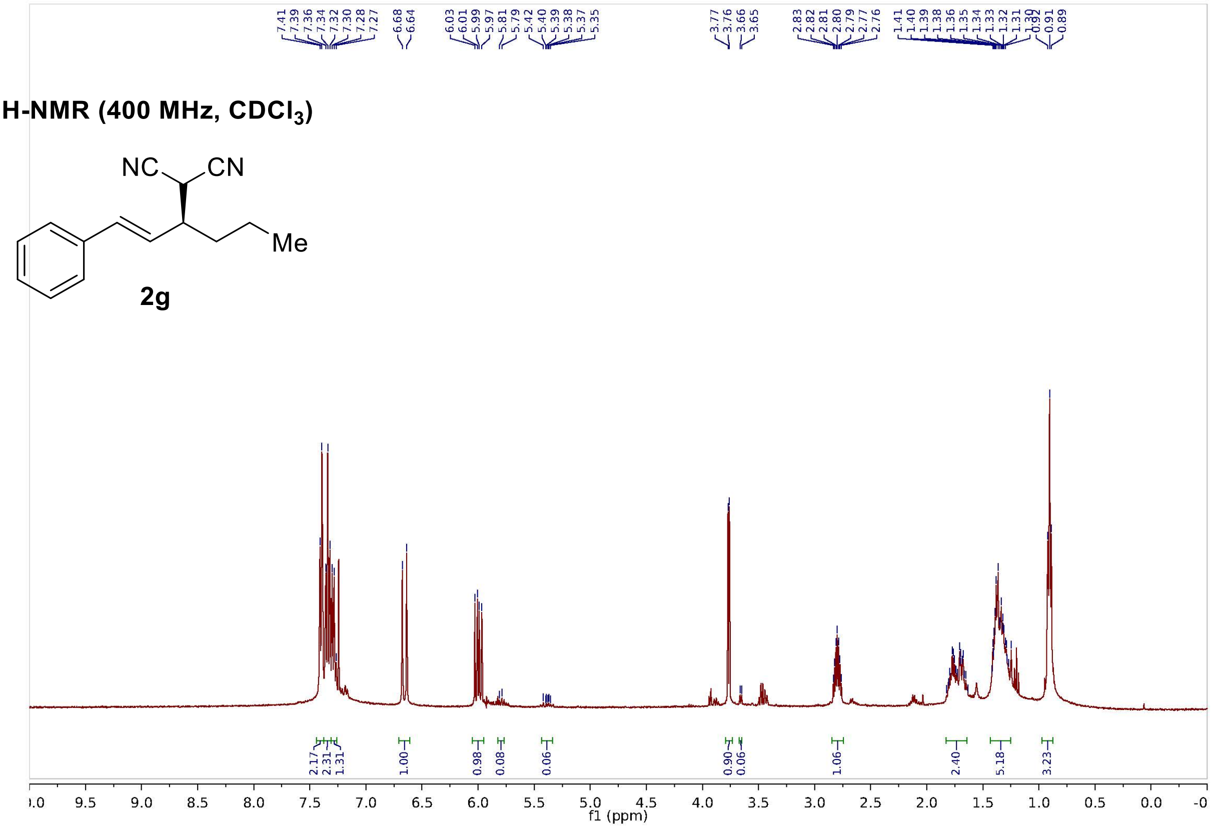

^{13}C-NMR (125 MHz, $CDCl_3$)

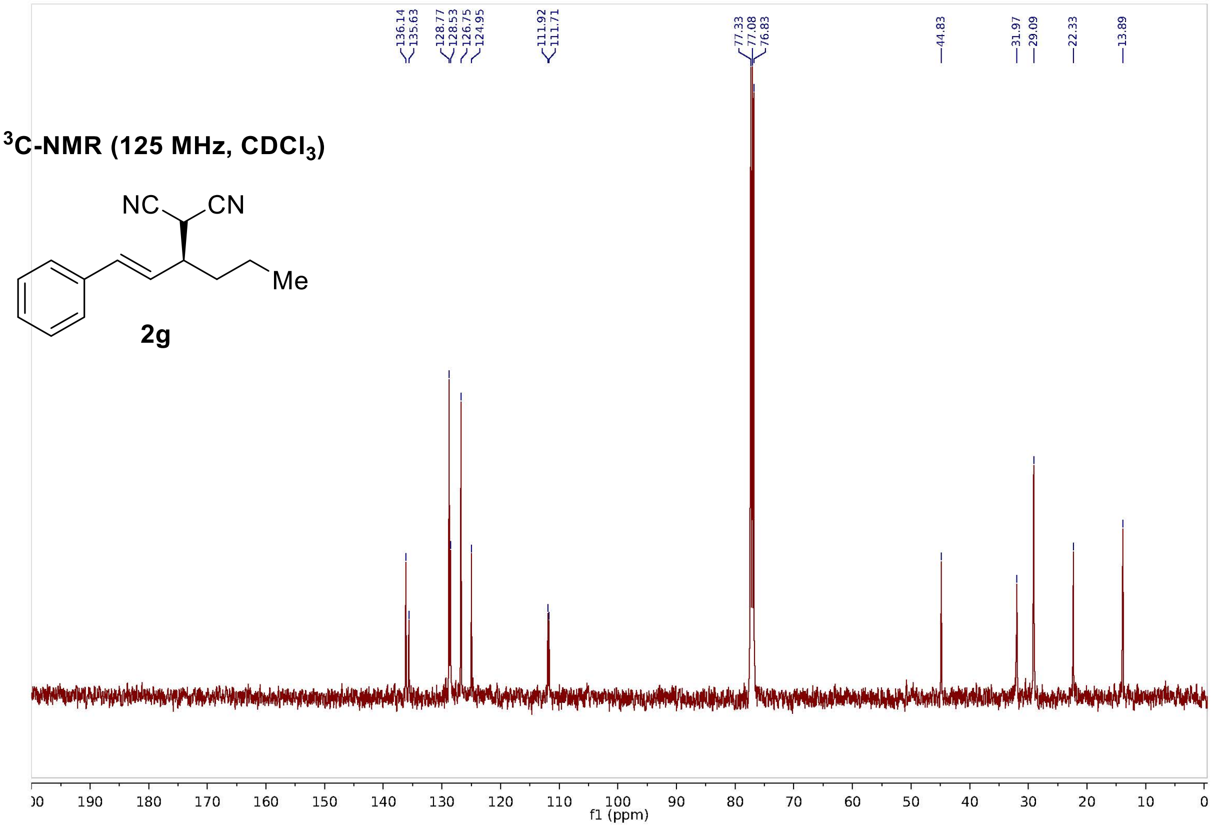

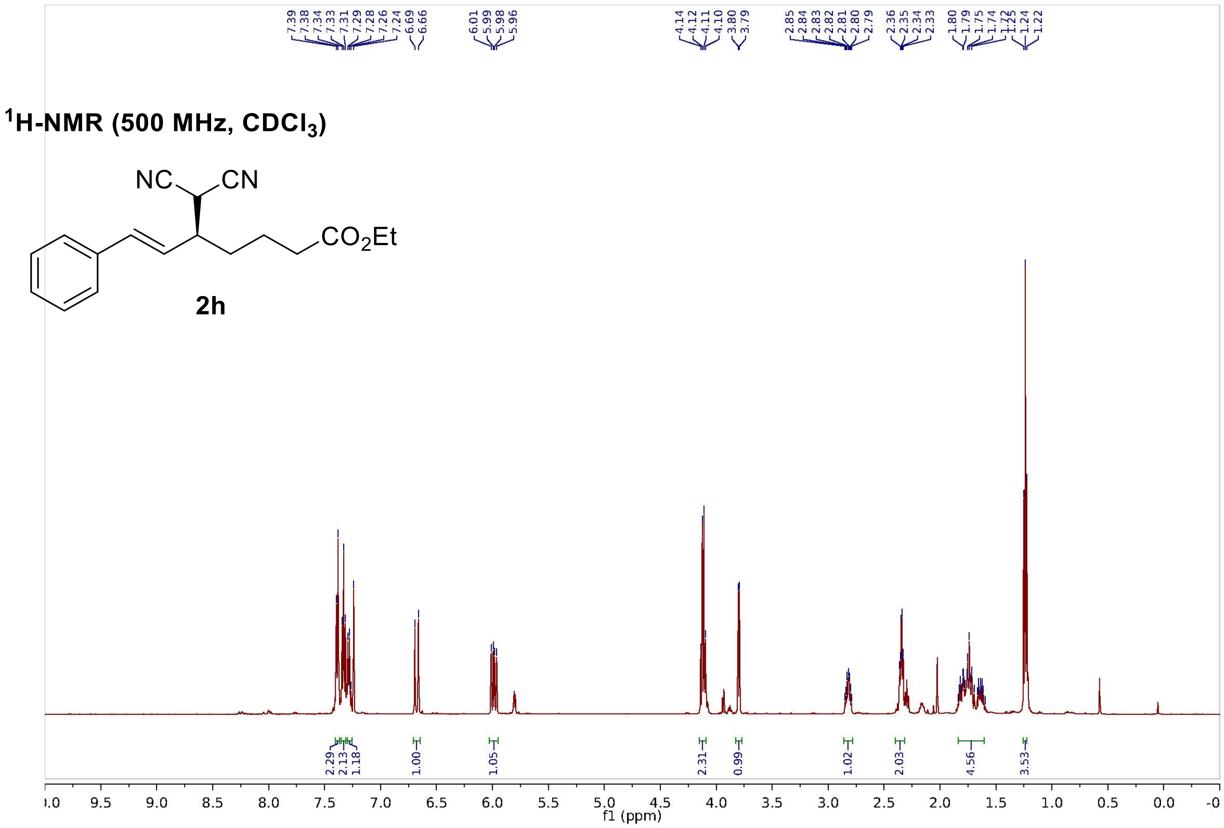

^{1}H-NMR (500 MHz, CDCl$_3$)
NC
CN
CO$_2$Et
2h
7.39 7.38 7.34 7.33 7.31 7.29 7.28 7.26 7.24 6.69 6.66
6.01 5.99 5.98 5.96
4.14 4.12 4.11 4.10 3.80 3.79
2.85 2.84 2.83 2.82 2.81 2.80 2.79 2.36 2.35 2.34 2.33
1.80 1.79 1.75 1.74 1.72 1.25 1.24 1.22
2.29 2.13 1.18 1.00 1.05 2.31 0.99 1.02 2.03 4.56 3.53
f1 (ppm)

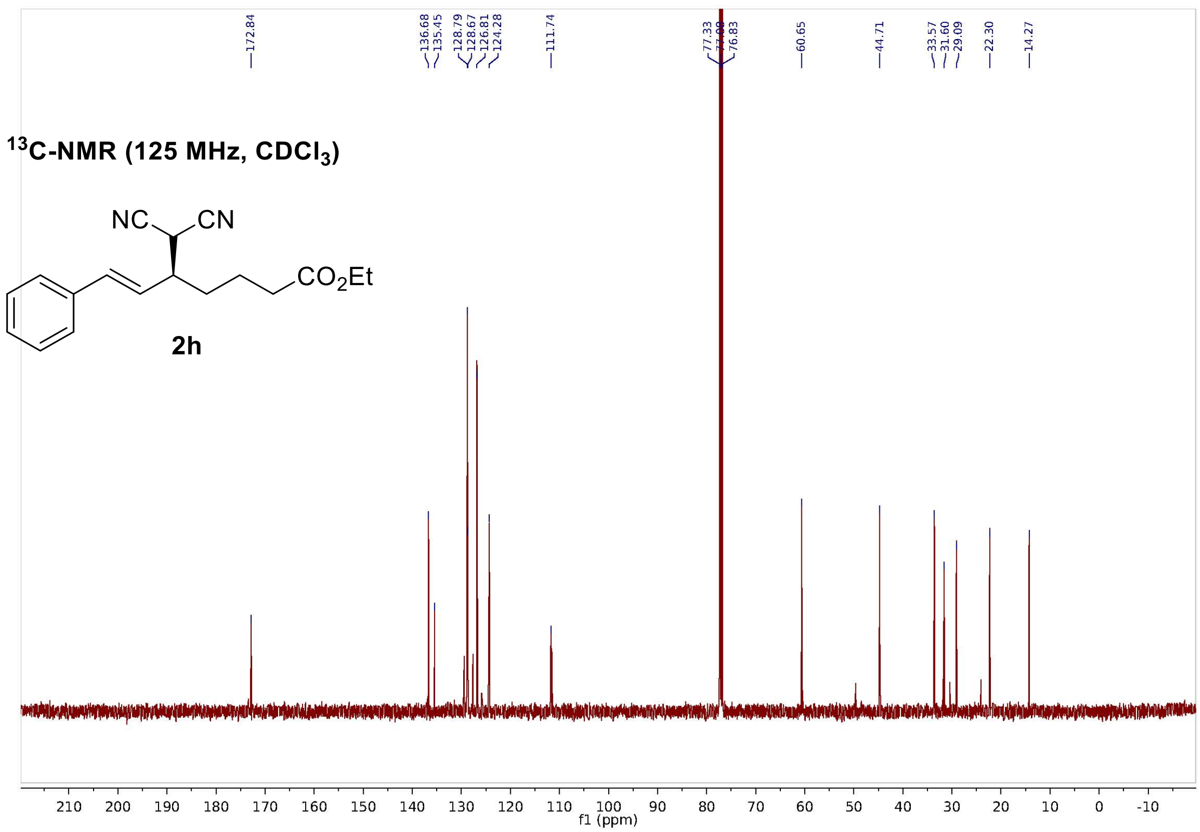

^{13}C-NMR (125 MHz, CDCl$_3$)
NC
CN
CO$_2$Et
2h
172.84
136.68 135.45 128.79 128.67 126.81 124.28
111.74
77.33 76.83
60.65
44.71
33.57 31.60 29.09
22.30
14.27
f1 (ppm)

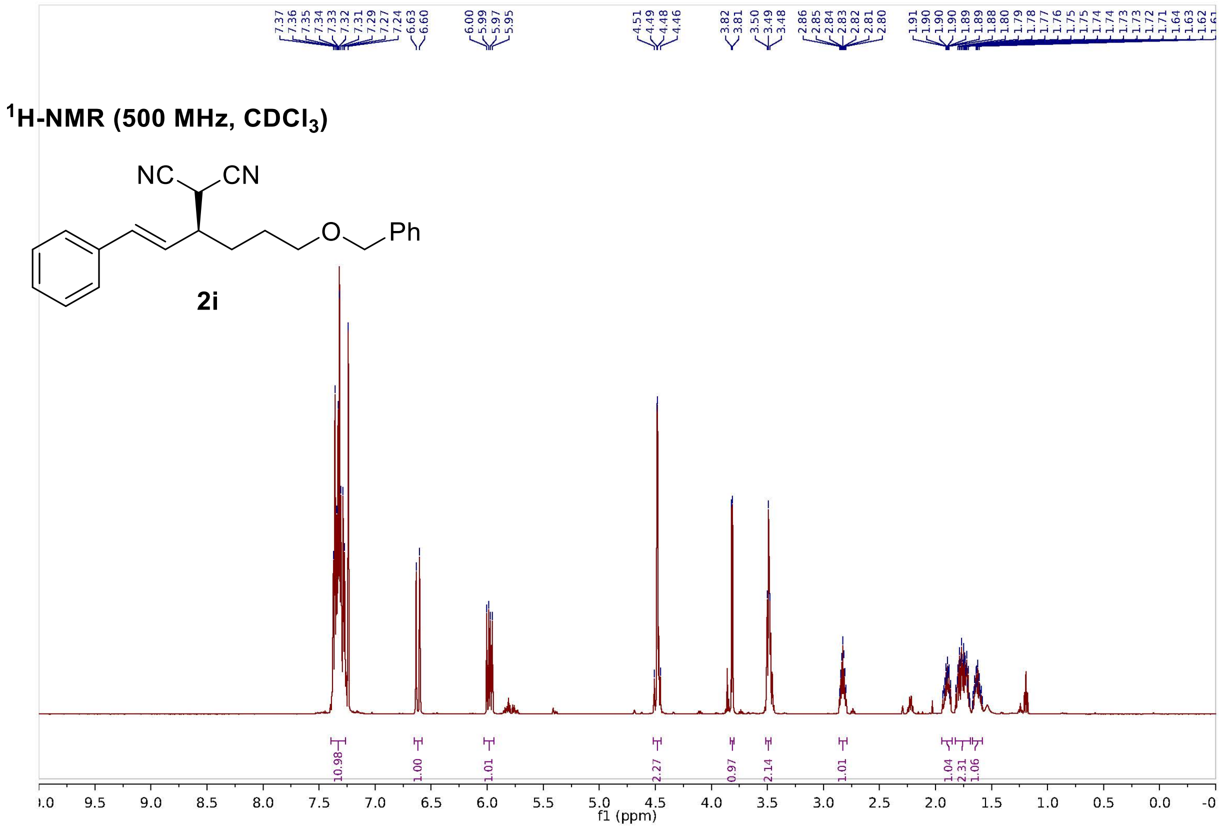
1H-NMR (500 MHz, CDCl3)
NC
CN
O
Ph
2i
f1 (ppm)

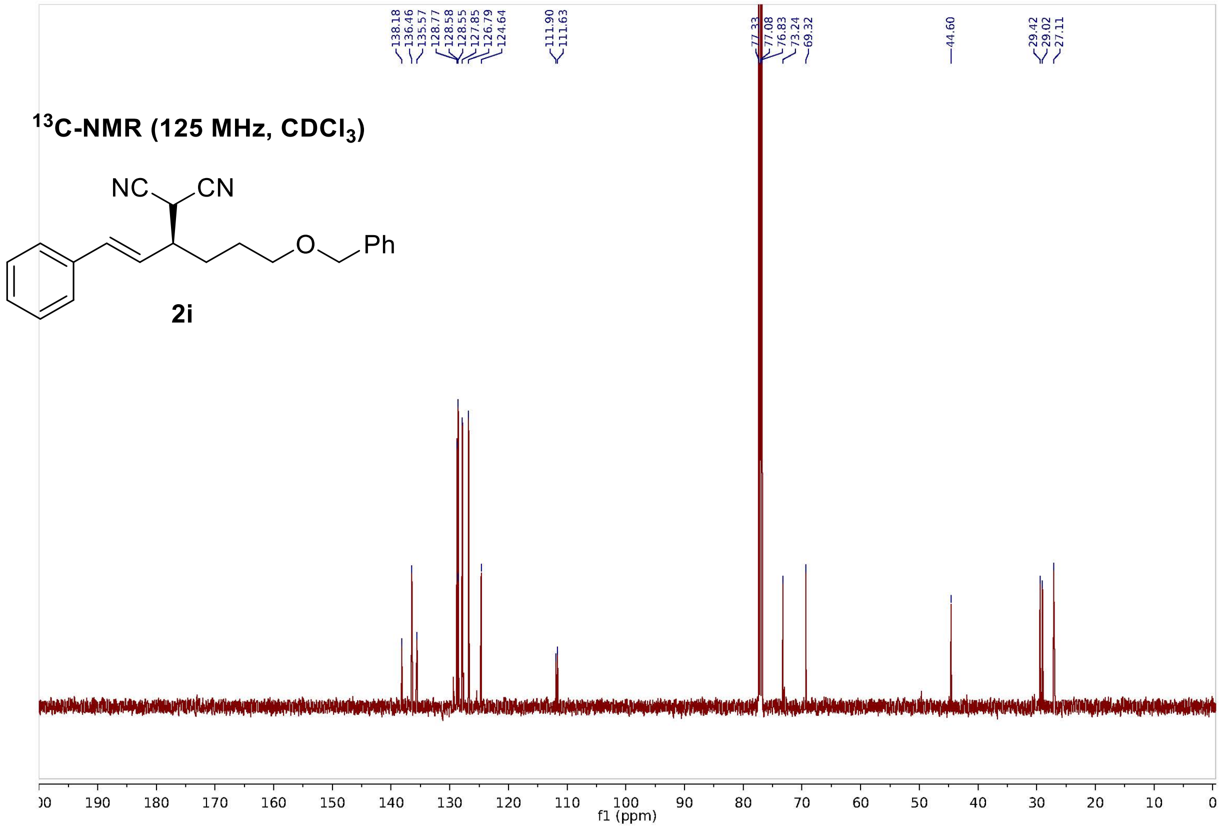
13C-NMR (125 MHz, CDCl3)
NC
CN
O
Ph
2i
f1 (ppm)

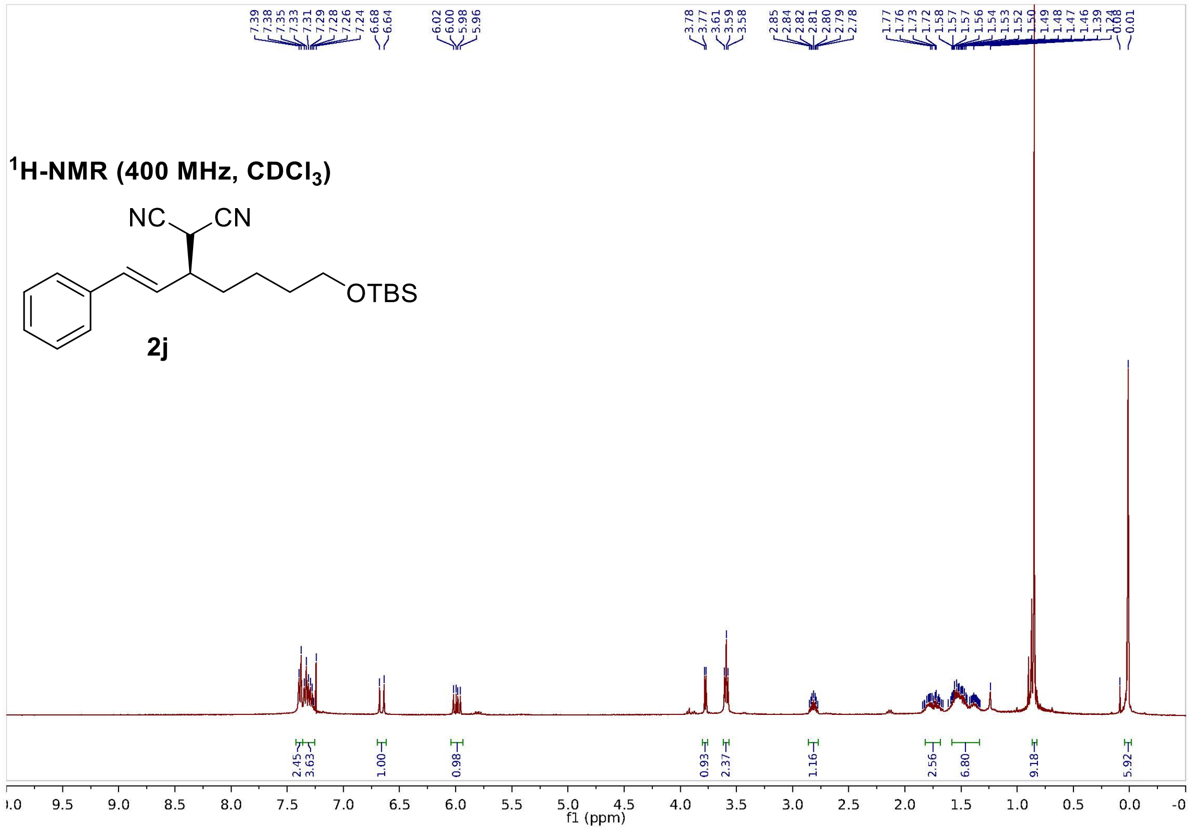
1H-NMR (400 MHz, CDCl3)
NC
CN
OTBS
2j
f1 (ppm)

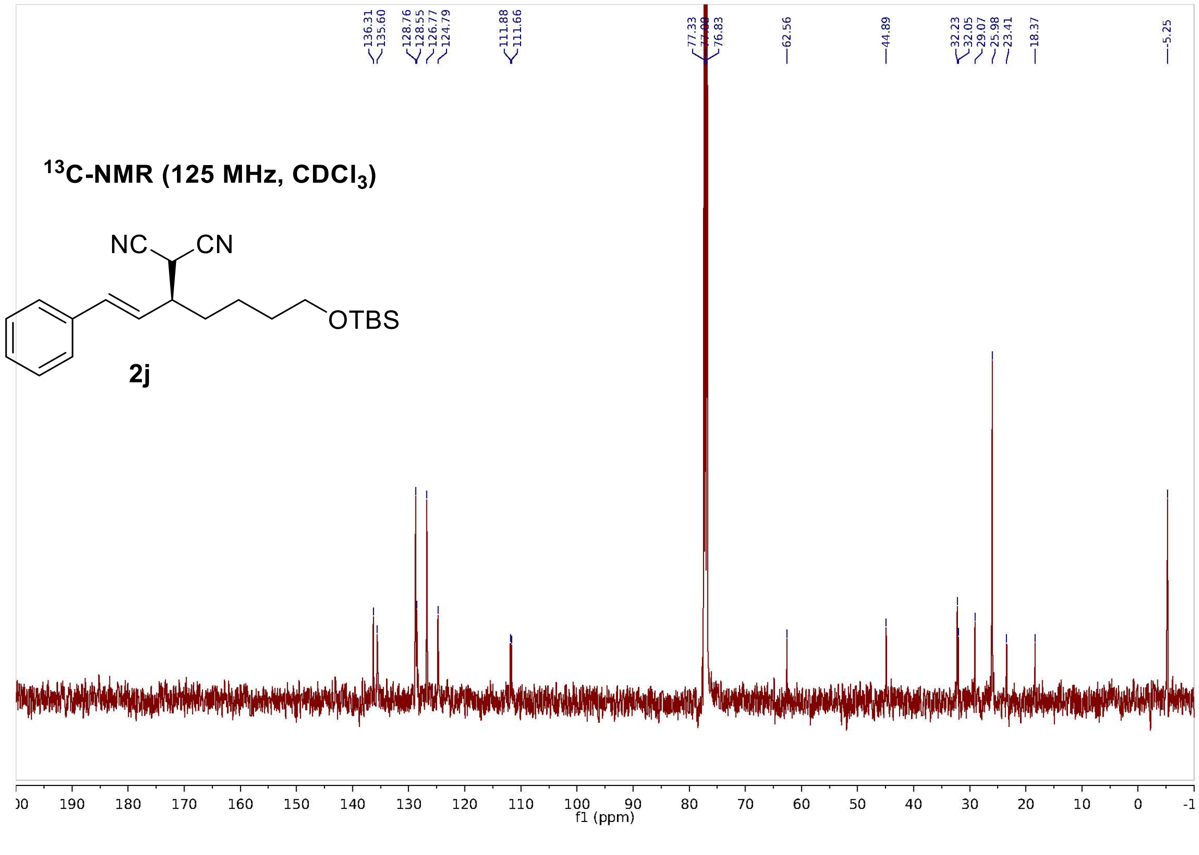
13C-NMR (125 MHz, CDCl3)
NC
CN
OTBS
2j
f1 (ppm)

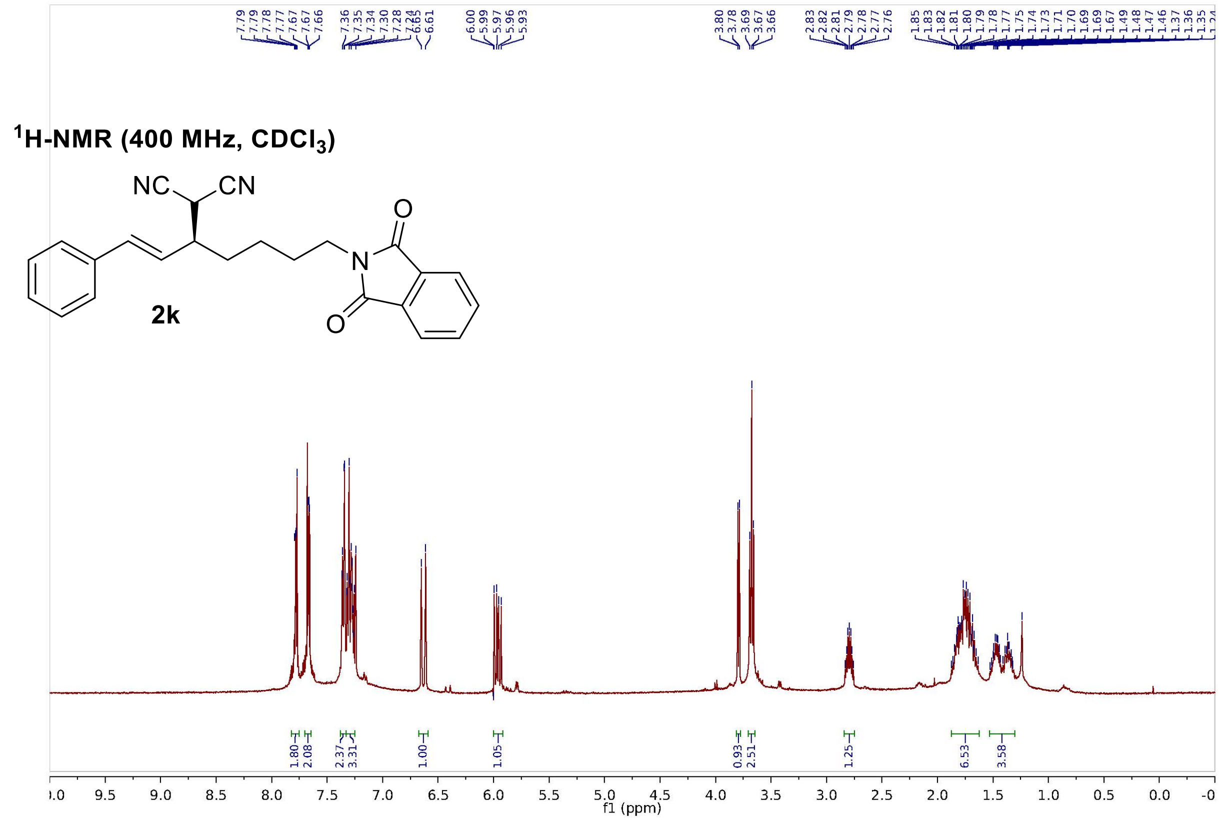
^{1}H-NMR (400 MHz, CDCl$_3$)
NC
CN
N
O
O
2k
f1 (ppm)

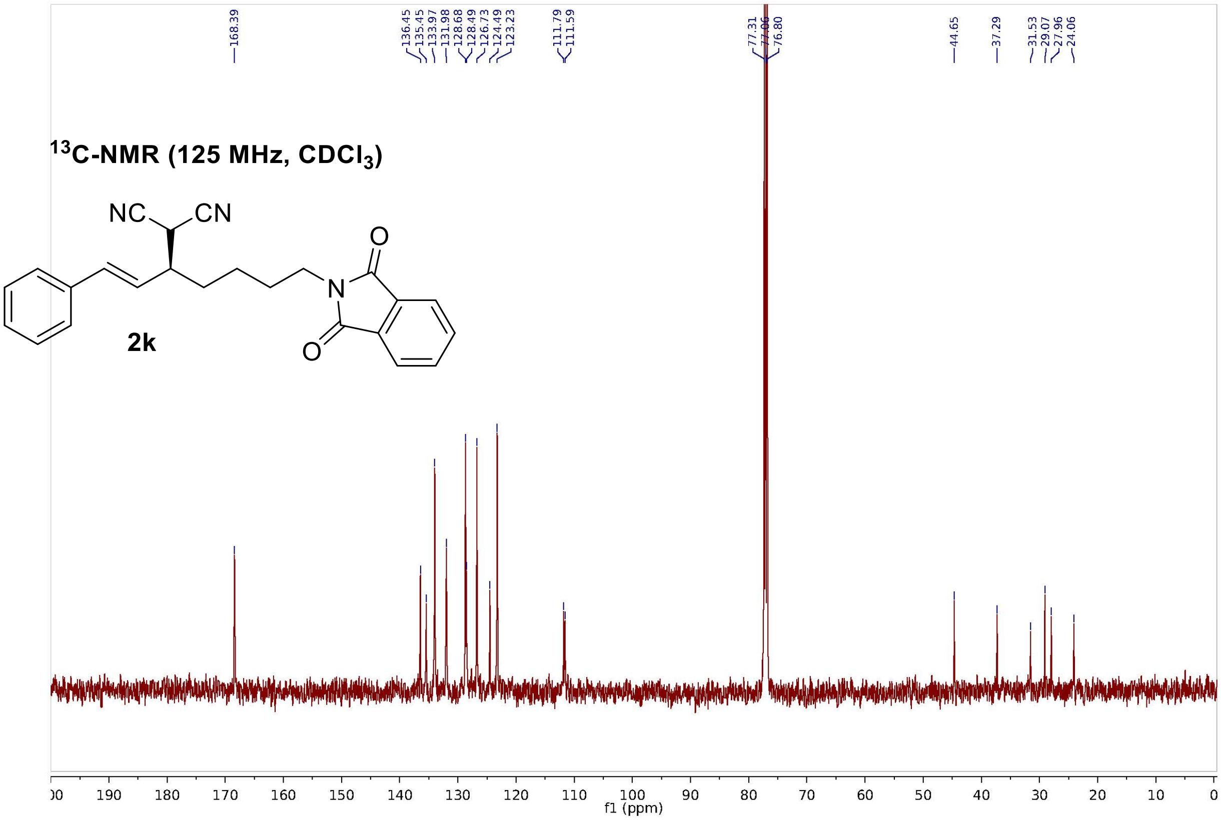
^{13}C-NMR (125 MHz, CDCl$_3$)
NC
CN
N
O
O
2k
f1 (ppm)

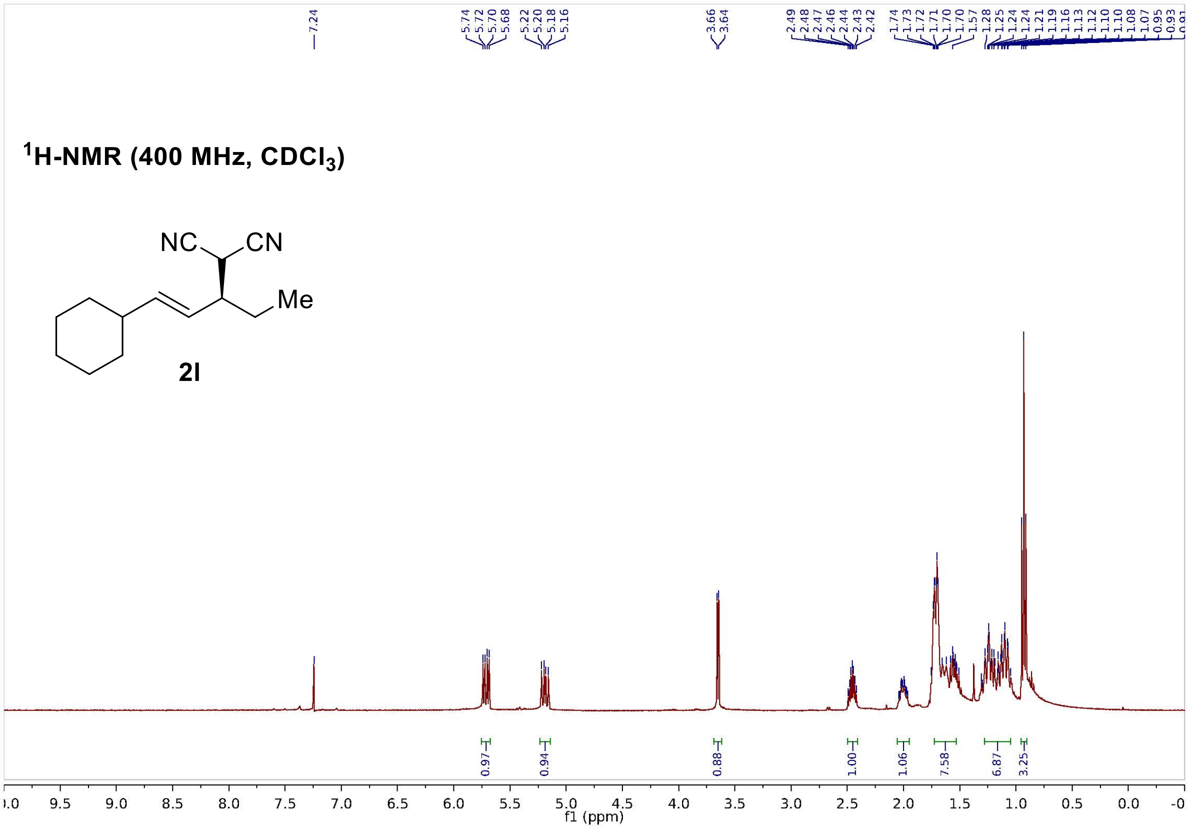

^{1}H-NMR (400 MHz, $CDCl_3$)
NC
CN
Me
2l
f1 (ppm)

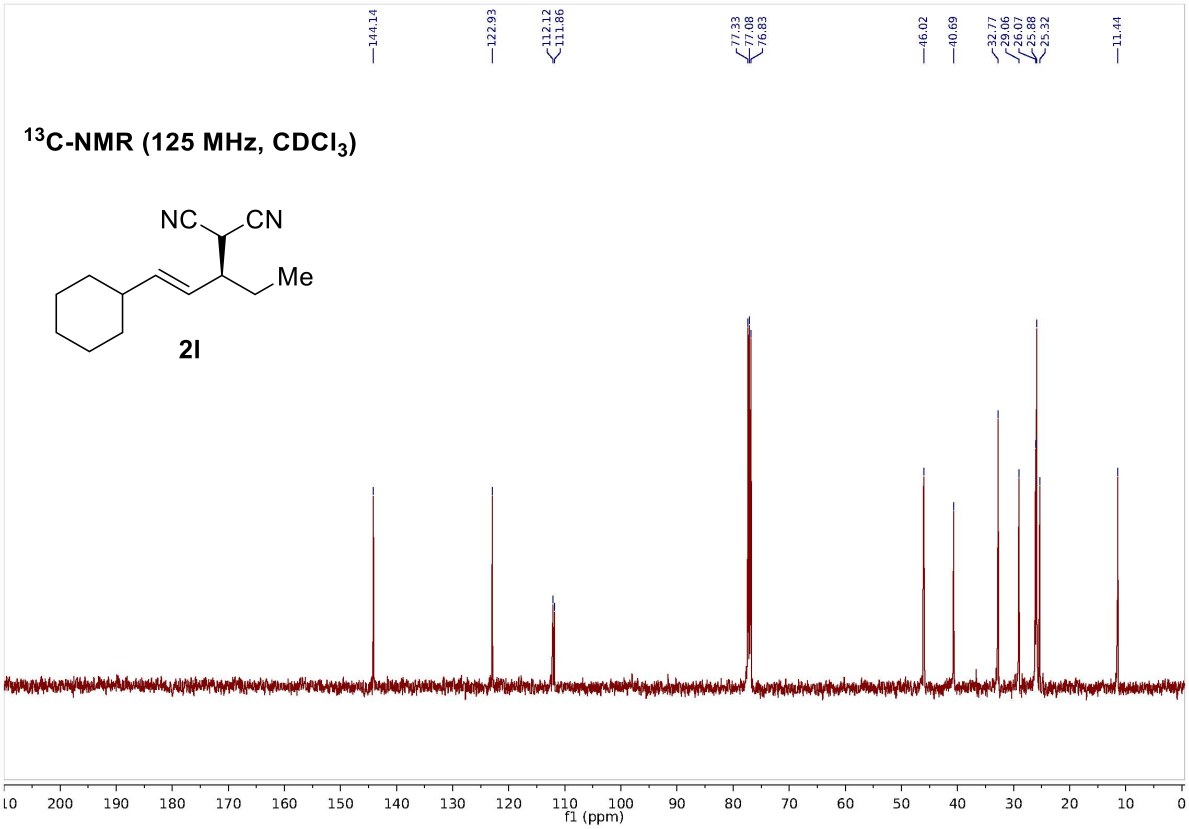

^{13}C-NMR (125 MHz, $CDCl_3$)
NC
CN
Me
2l
f1 (ppm)

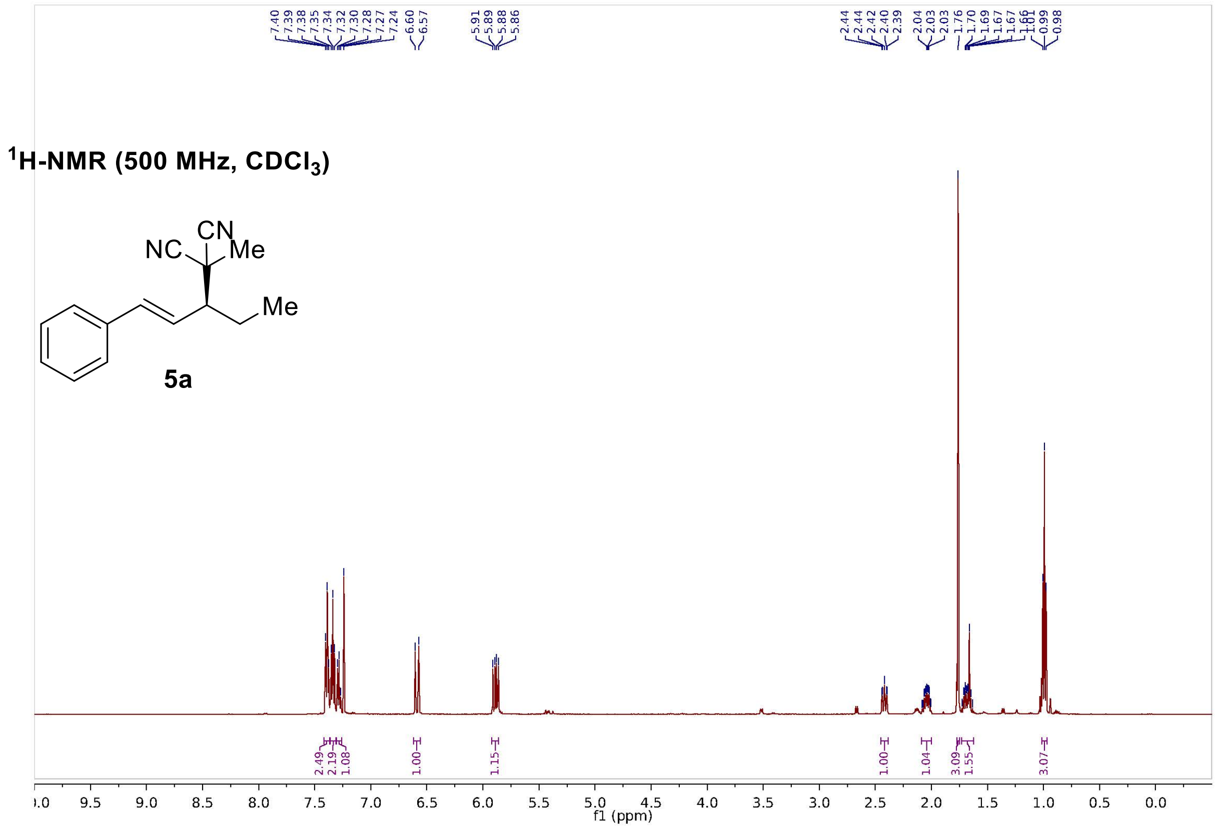

1H-NMR (500 MHz, CDCl3)
7.40 7.39 7.38 7.35 7.34 7.32 7.30 7.28 7.27 7.24 6.60 6.57 5.91 5.89 5.88 5.86 2.44 2.44 2.42 2.40 2.39 2.04 2.03 2.03 1.76 1.70 1.69 1.67 1.67 1.66 1.01 0.99 0.98
2.49 2.19 1.08 1.00 1.15 1.00 1.04 3.09 1.55 3.07
CN
NC
Me
Me
5a
f1 (ppm)

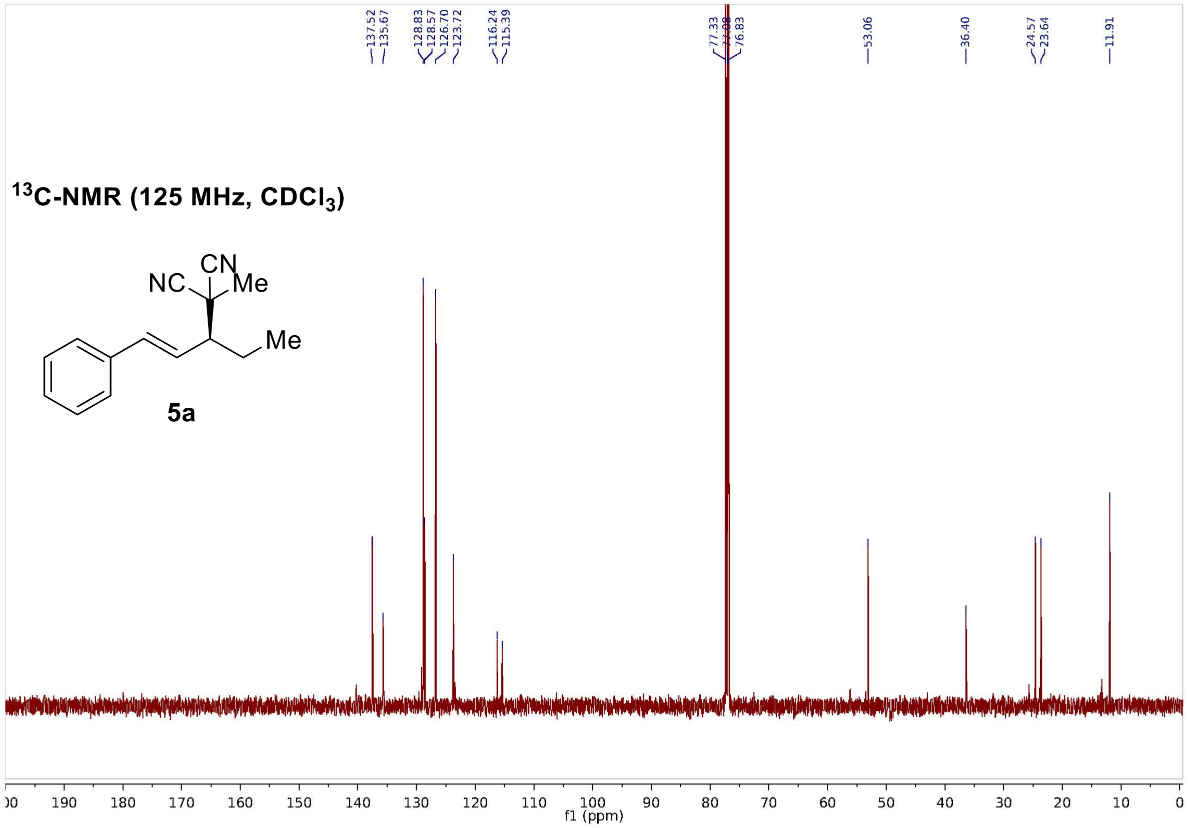

13C-NMR (125 MHz, CDCl3)
137.52 135.67 128.83 128.57 126.70 123.72 116.24 115.39 77.33 77.08 76.83 53.06 36.40 24.57 23.64 11.91
CN
NC
Me
Me
5a
f1 (ppm)

^{1}H-NMR (400 MHz, $CDCl_3$)

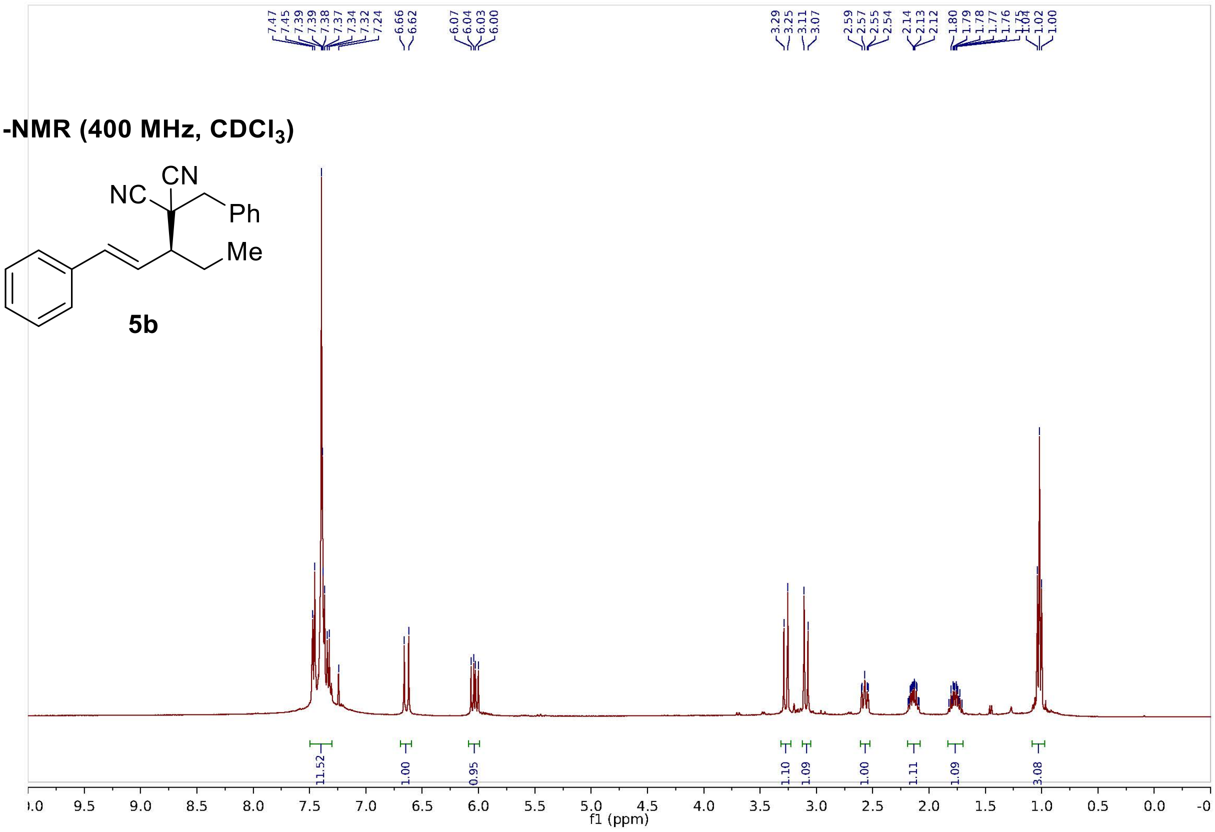

5b

^{13}C-NMR (125 MHz, $CDCl_3$)

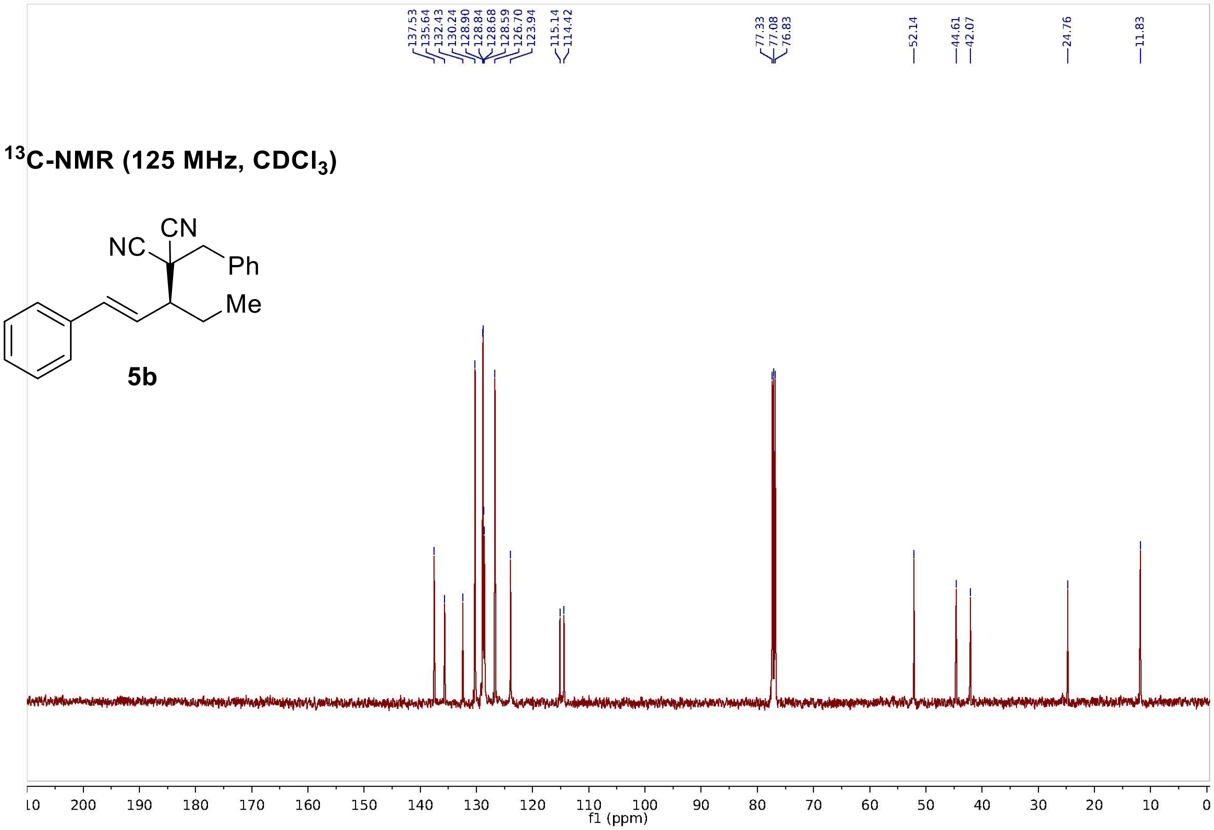

5b

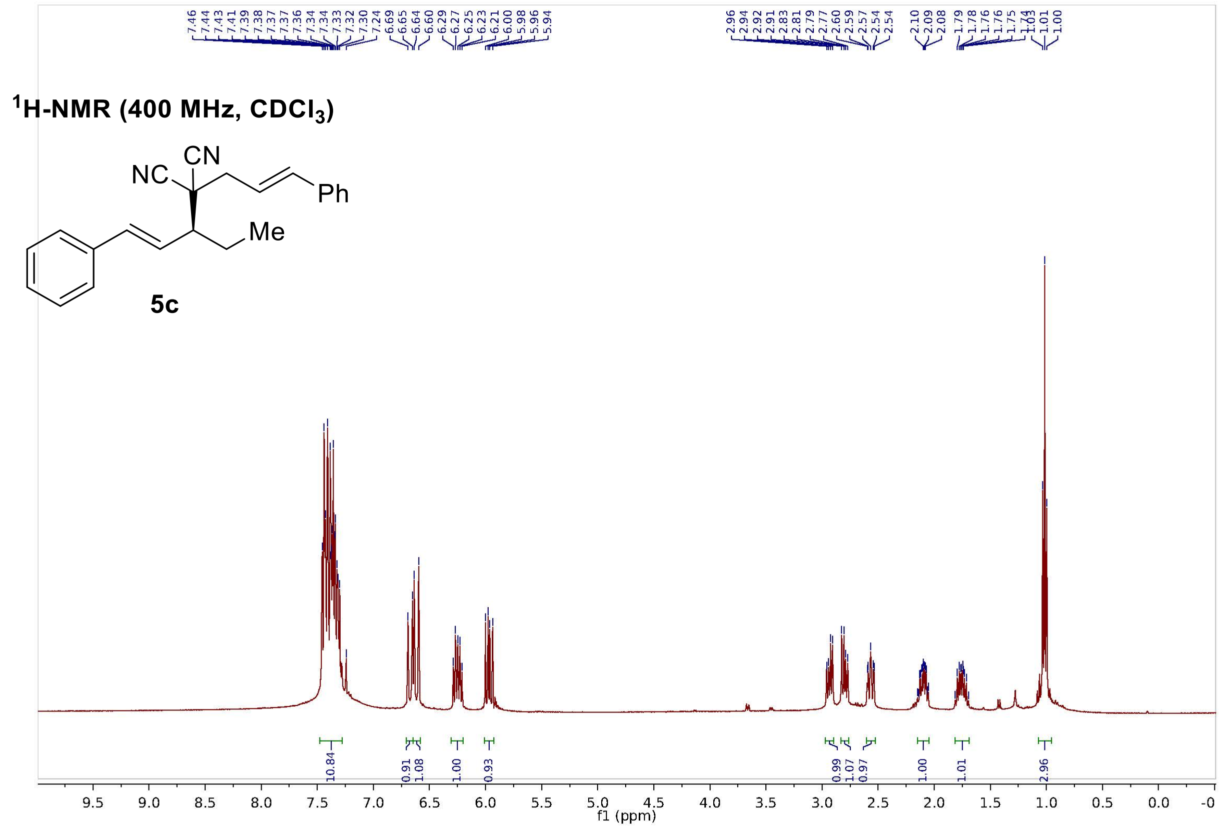
1H-NMR (400 MHz, CDCl3)
NC
CN
Ph
Me
5c
7.46 7.44 7.43 7.41 7.39 7.38 7.37 7.37 7.36 7.34 7.34 7.33 7.32 7.30 7.24 6.69 6.65 6.64 6.60 6.29 6.27 6.25 6.23 6.21 6.00 5.98 5.96 5.94
2.96 2.94 2.92 2.91 2.83 2.81 2.79 2.77 2.60 2.59 2.57 2.54 2.54 2.10 2.09 2.08 1.79 1.78 1.76 1.76 1.75 1.74 1.03 1.01 1.00
10.84 0.91 1.08 1.00 0.93 0.99 1.07 0.97 1.00 1.01 2.96
f1 (ppm)

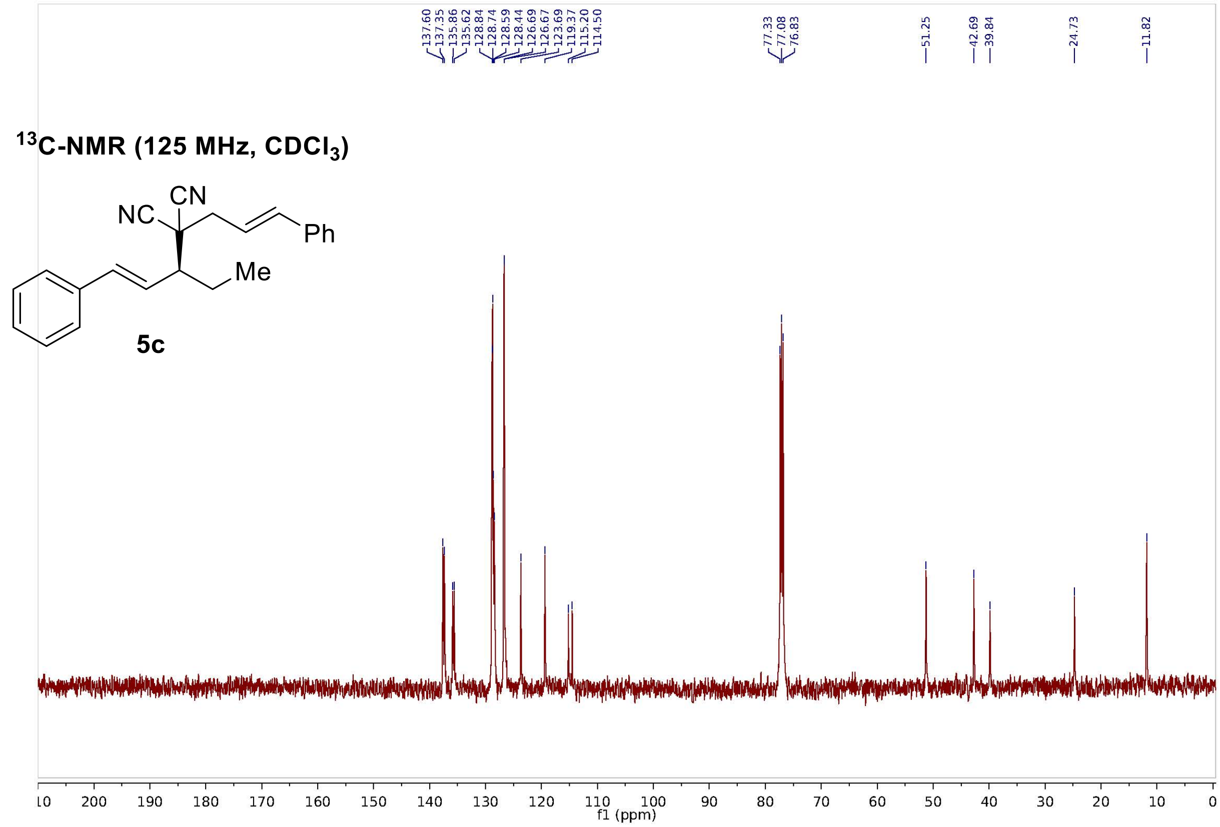
13C-NMR (125 MHz, CDCl3)
NC
CN
Ph
Me
5c
137.60 137.35 135.86 135.62 128.84 128.74 128.59 128.44 126.69 126.67 123.69 119.37 115.20 114.50
77.33 77.08 76.83
51.25
42.69 39.84
24.73
11.82
f1 (ppm)

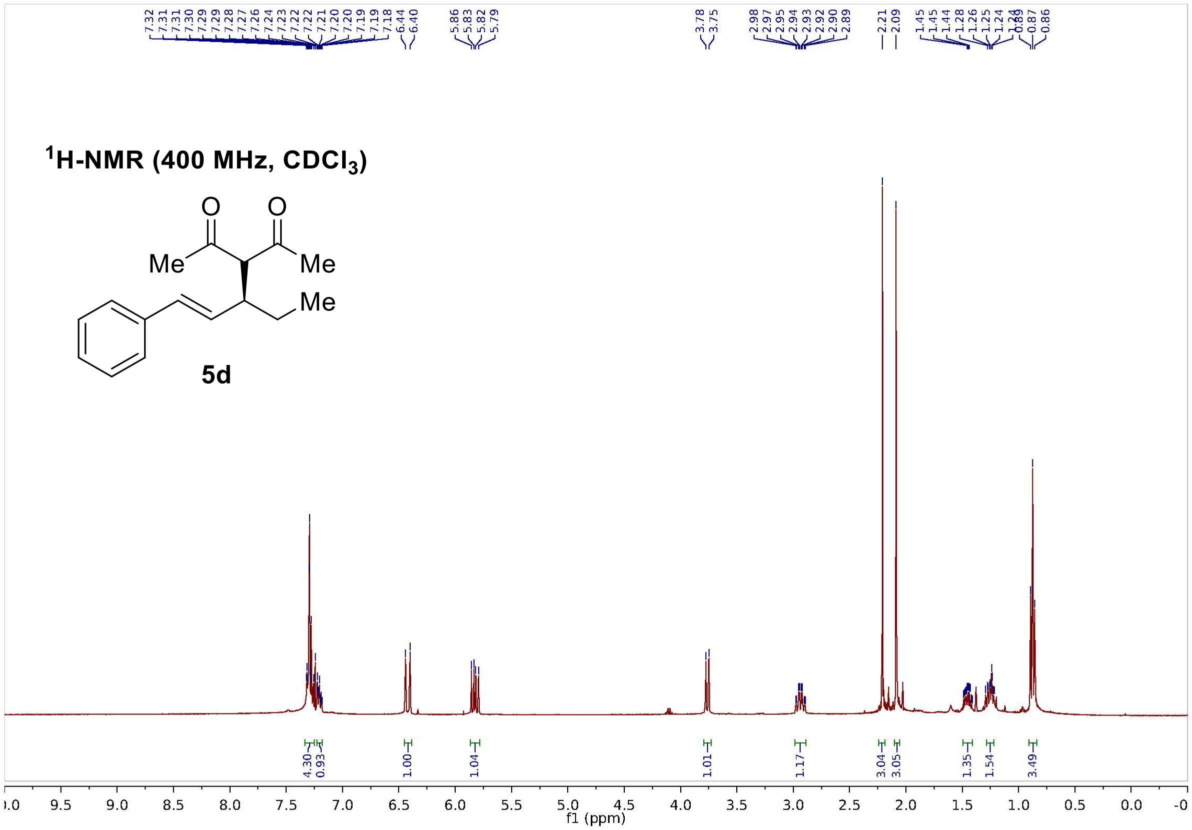
1H-NMR (400 MHz, CDCl3)
O
O
Me
Me
Me
5d
7.32 7.31 7.31 7.30 7.29 7.29 7.28 7.27 7.26 7.24 7.23 7.22 7.22 7.21 7.20 7.20 7.19 7.19 7.18 6.44 6.40
5.86 5.83 5.82 5.79
3.78 3.75
2.98 2.97 2.95 2.94 2.93 2.92 2.90 2.89
2.21 2.09
1.45 1.45 1.44 1.28 1.26 1.25 1.24 0.89 0.87 0.86
4.30 0.93 1.00 1.04 1.01 1.17 3.04 3.05 1.35 1.54 3.49
f1 (ppm)

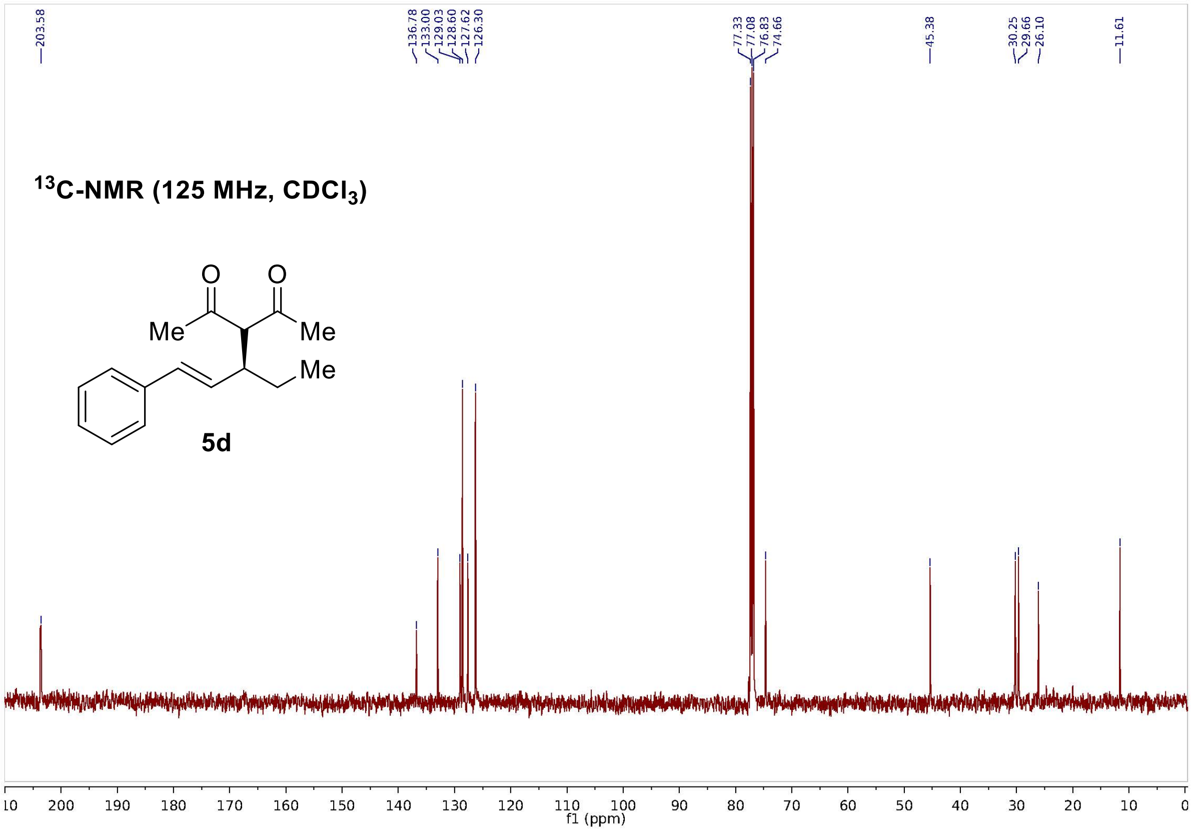
203.58
136.78 133.00 129.03 128.60 127.62 126.30
77.33 77.08 76.83 74.66
45.38
30.25 29.66 26.10
11.61
13C-NMR (125 MHz, CDCl3)
O
O
Me
Me
Me
5d
f1 (ppm)

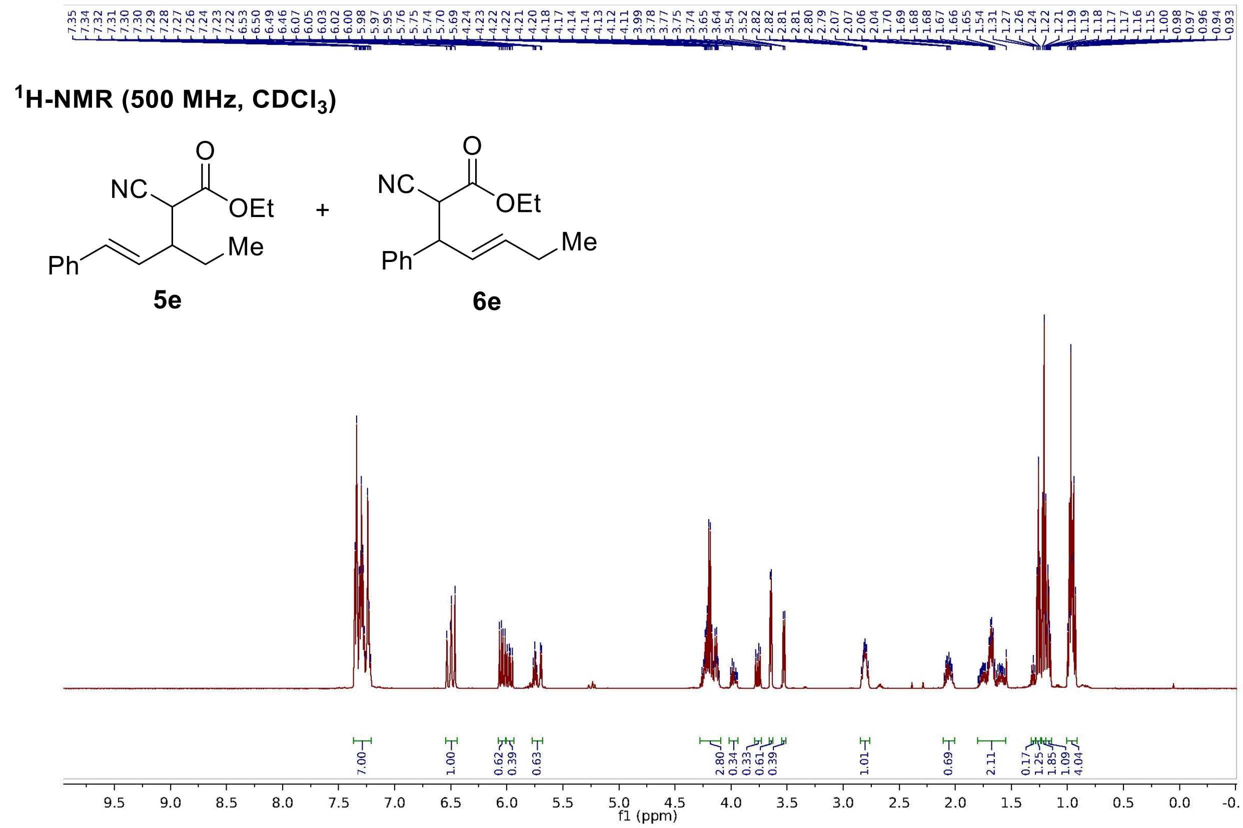
1H-NMR (500 MHz, CDCl3)
NC
O
OEt
Me
Ph
5e
+
6e
f1 (ppm)

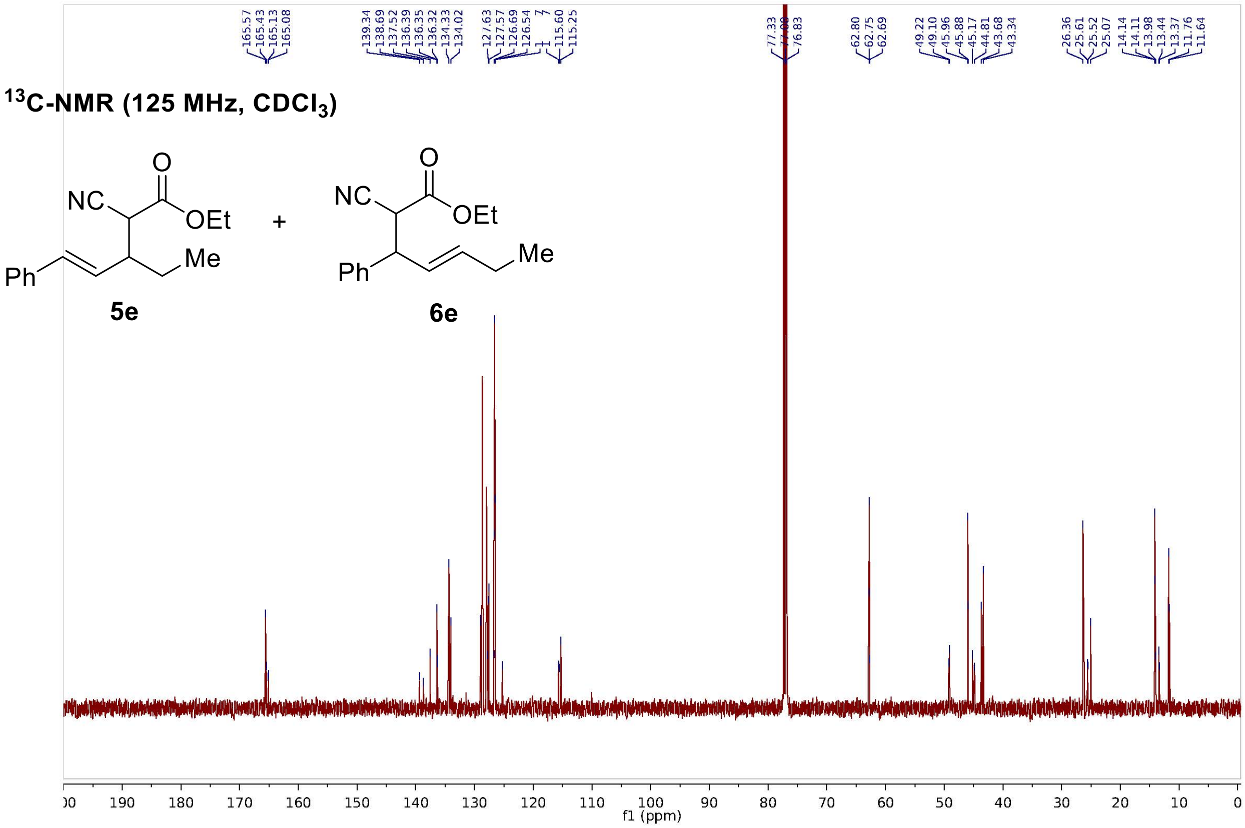
13C-NMR (125 MHz, CDCl3)
NC
O
OEt
Me
Ph
5e
+
6e
f1 (ppm)

^{1}H-COSY-NMR (500 MHz, CDCl$_3$)

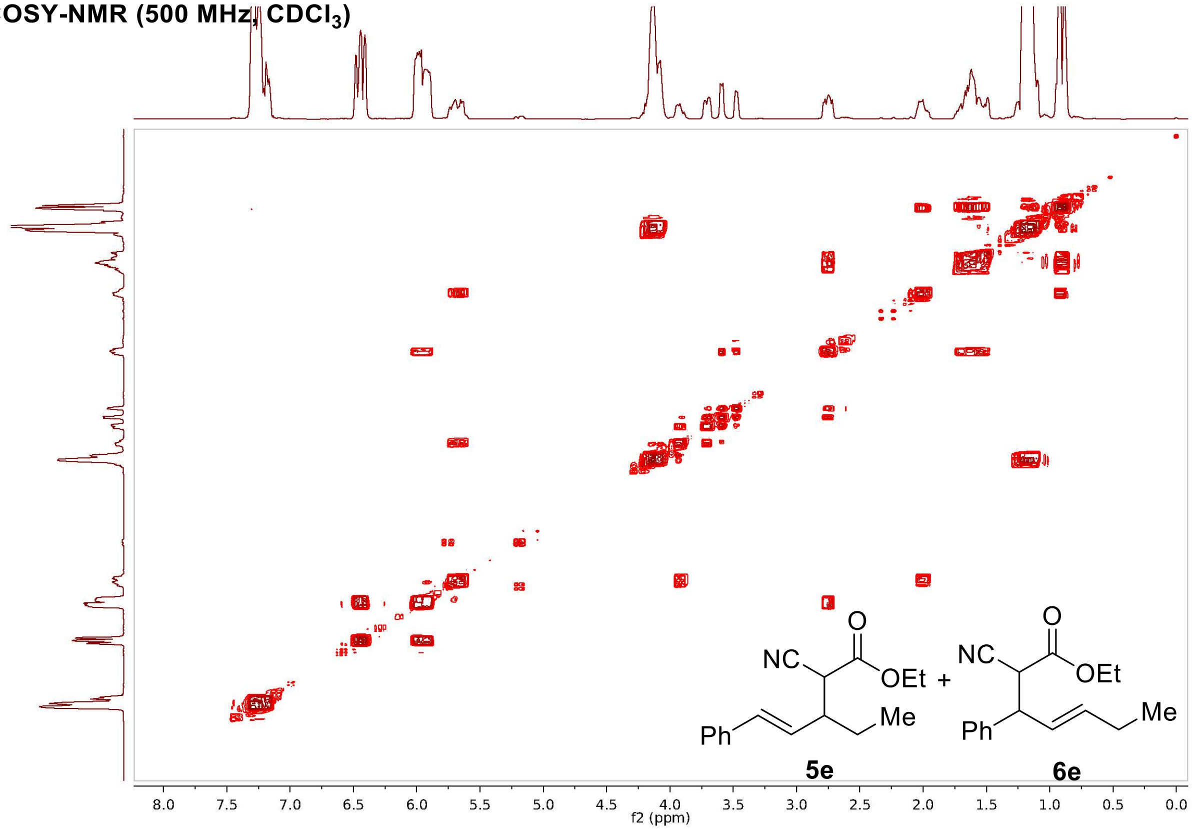

^{1}H-NMR (500 MHz, CDCl$_3$)

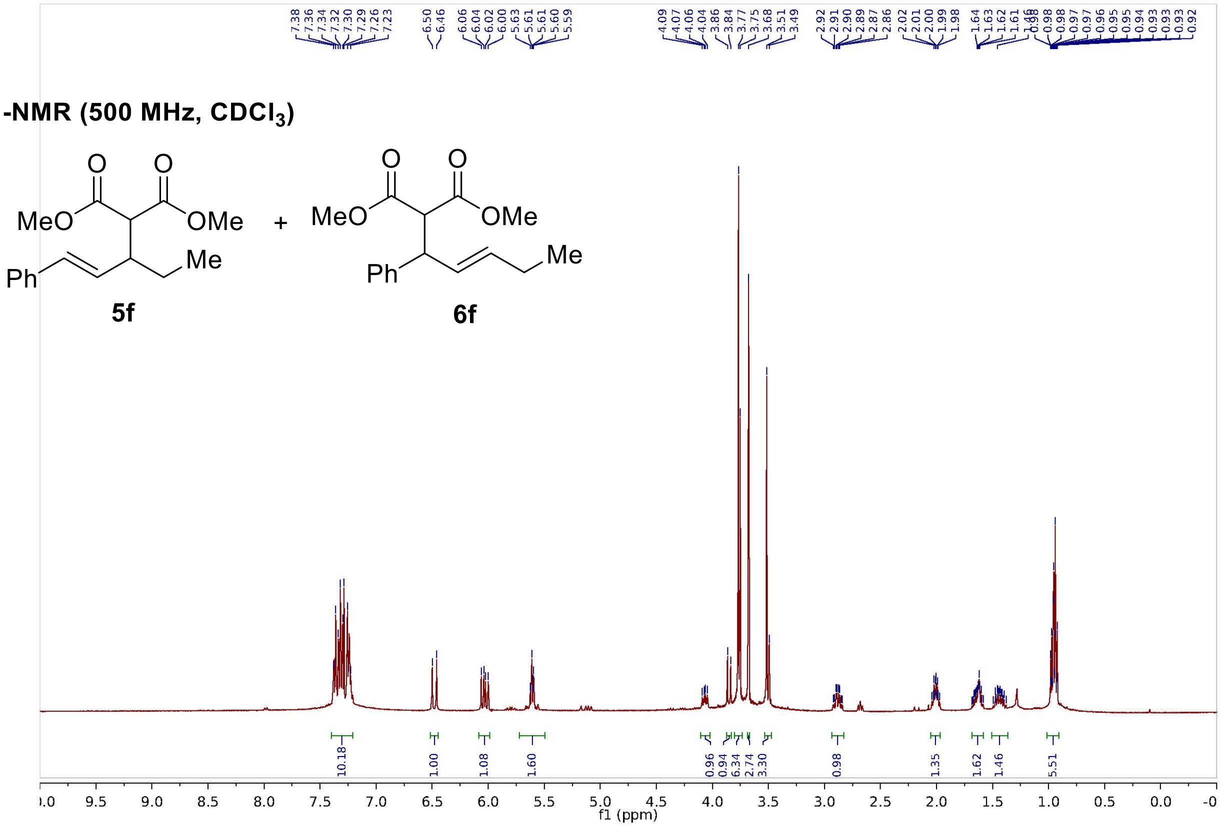

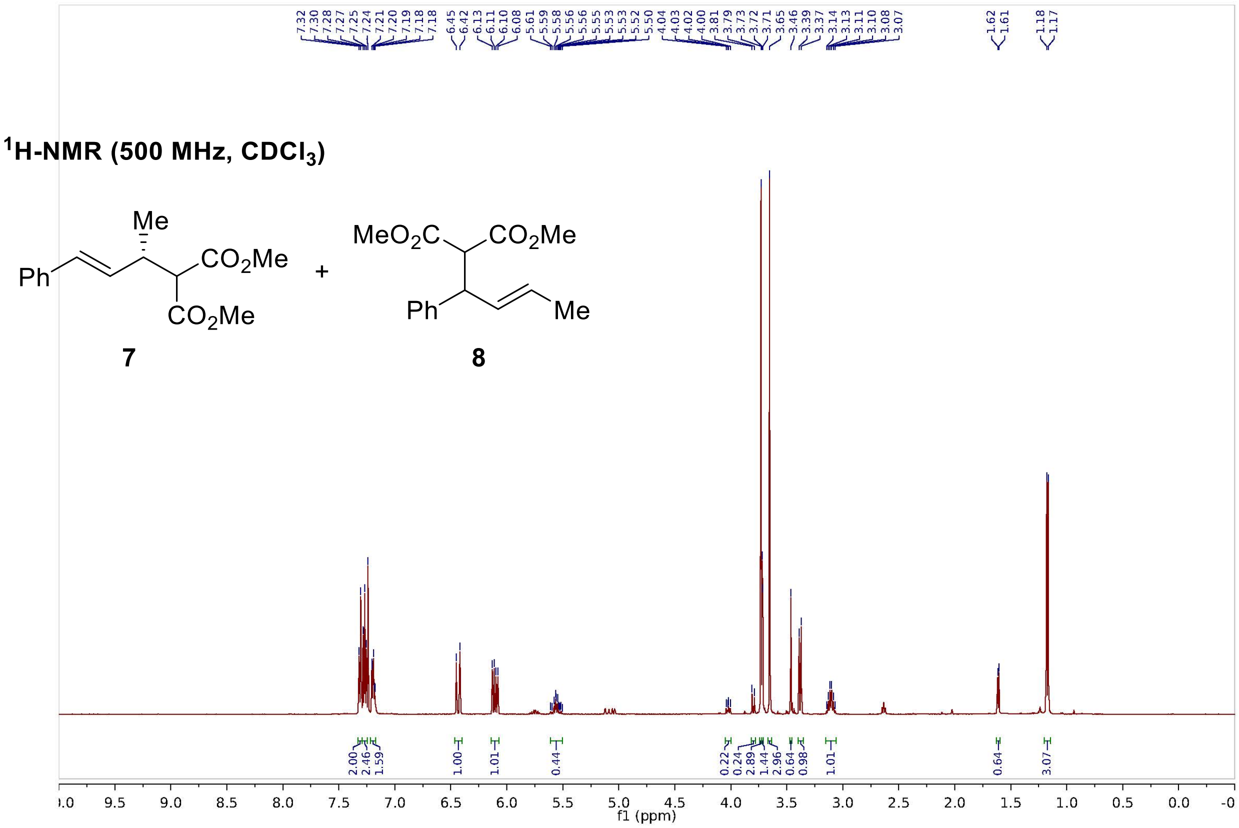
1H-NMR (500 MHz, CDCl3)
Me
Ph
CO2Me
CO2Me
+
MeO2C
CO2Me
Ph
Me
7
8
f1 (ppm)

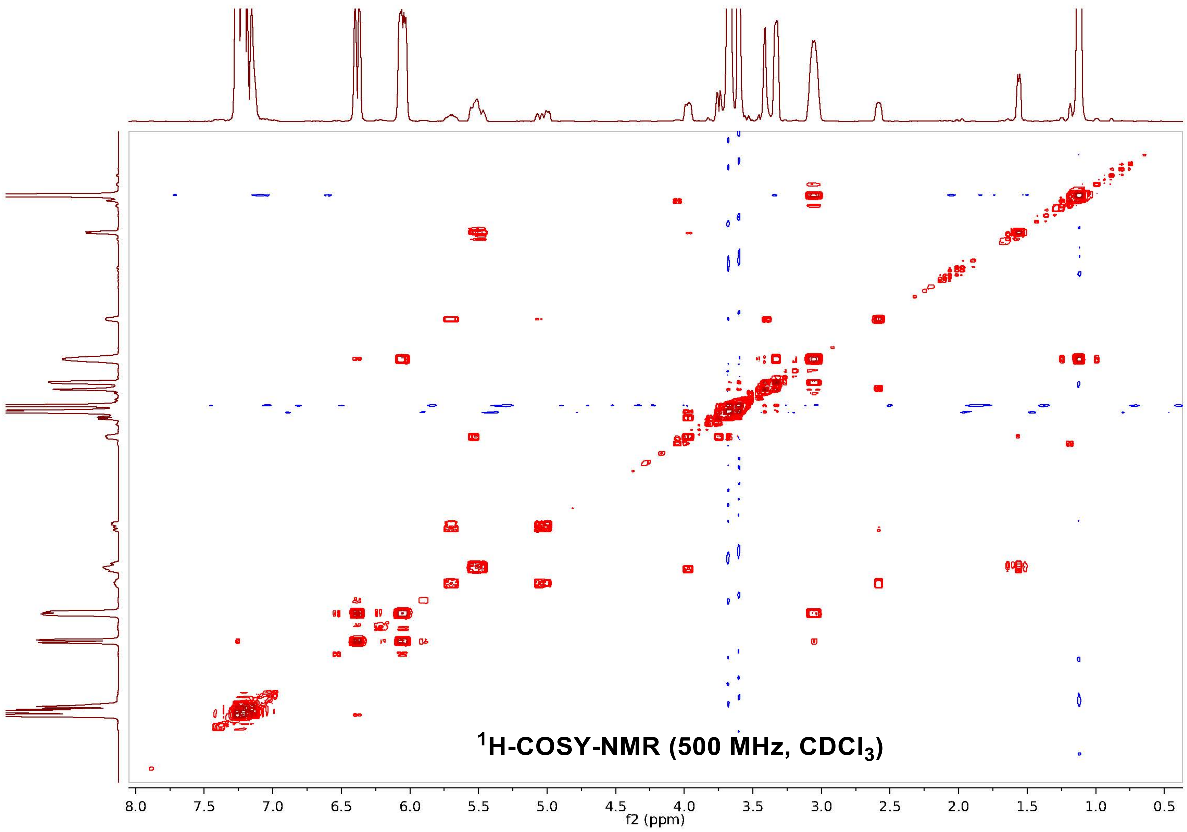
1H-COSY-NMR (500 MHz, CDCl3)
f2 (ppm)

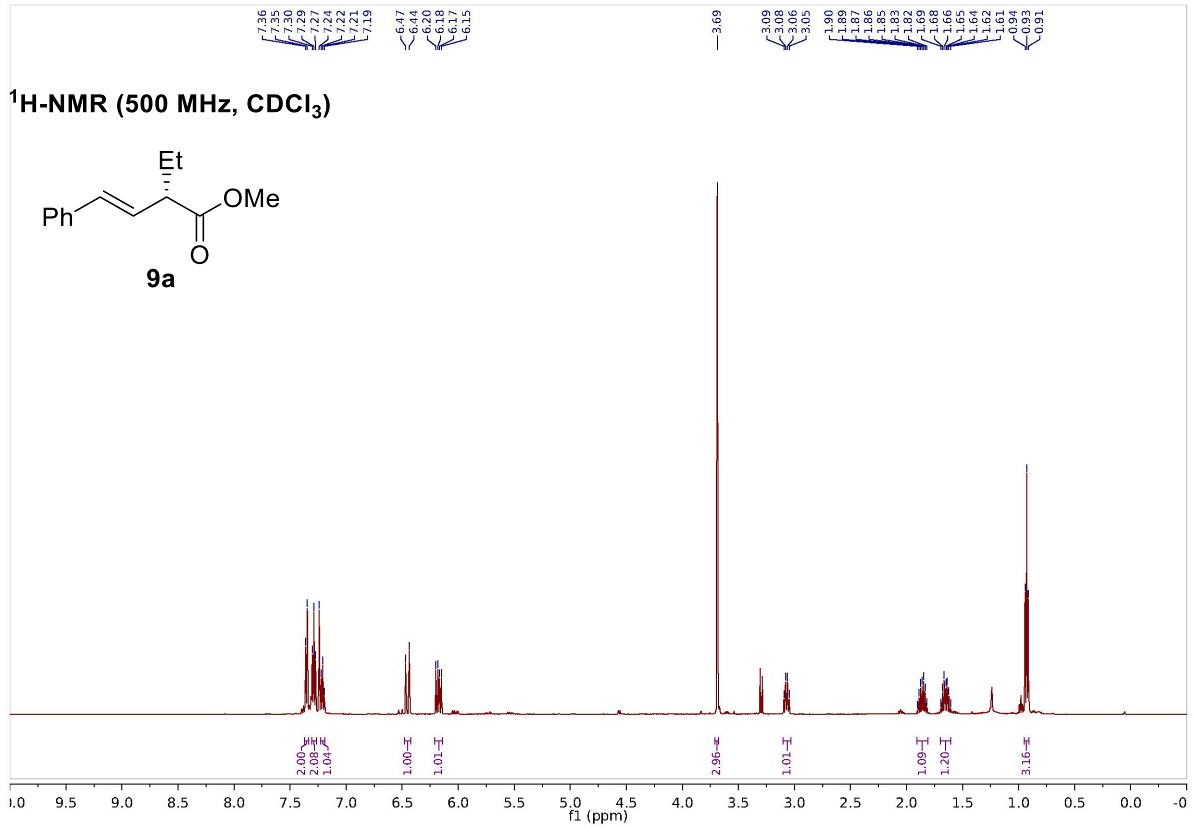

7.36 7.35 7.30 7.29 7.27 7.24 7.22 7.21 7.19 6.47 6.44 6.20 6.18 6.17 6.15 3.69 3.09 3.08 3.06 3.05 1.90 1.89 1.87 1.86 1.85 1.83 1.82 1.69 1.68 1.66 1.65 1.64 1.62 1.61 0.94 0.93 0.91
^{1}H-NMR (500 MHz, $CDCl_3$)
Et
OMe
Ph
O
9a
2.00 2.08 1.04 1.00 1.01 2.96 1.01 1.09 1.20 3.16
f1 (ppm)

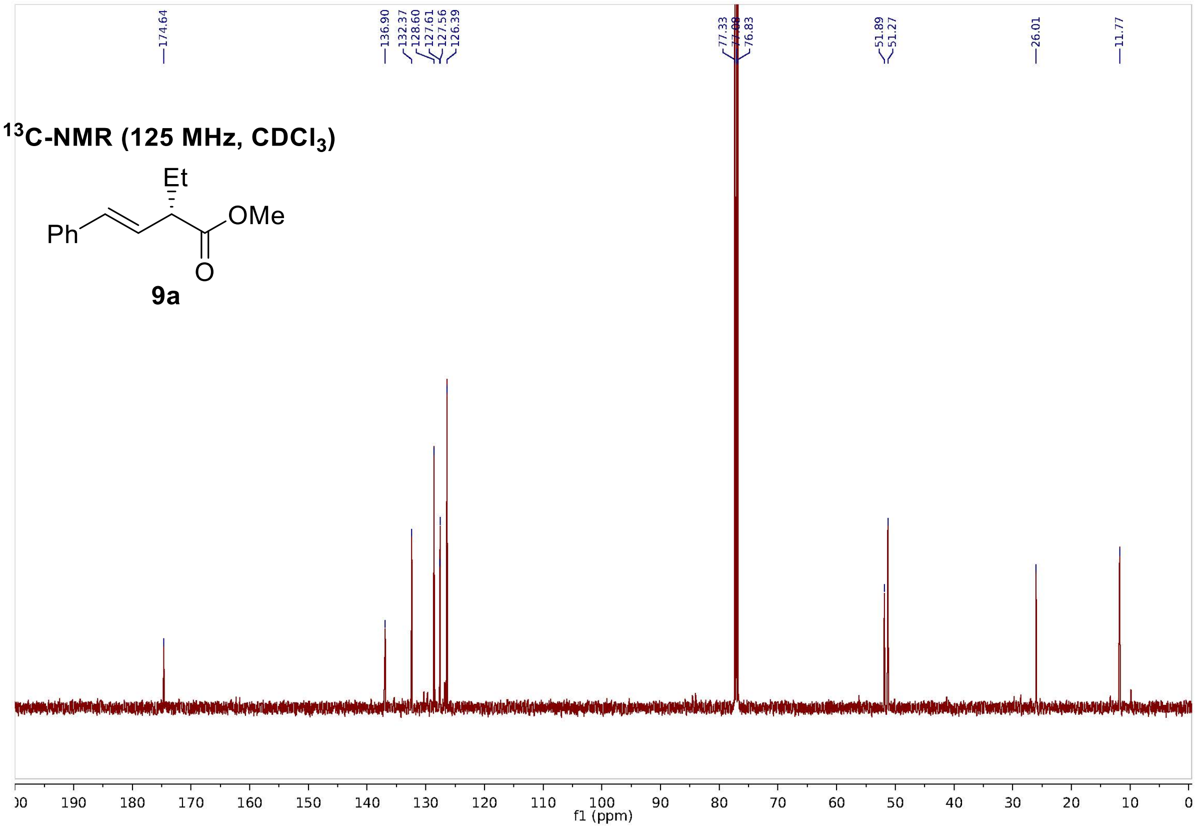

174.64 136.90 132.37 128.60 127.61 127.56 126.39 77.33 [illegible] 76.83 51.89 51.27 26.01 11.77
^{13}C-NMR (125 MHz, $CDCl_3$)
Et
OMe
Ph
O
9a
f1 (ppm)

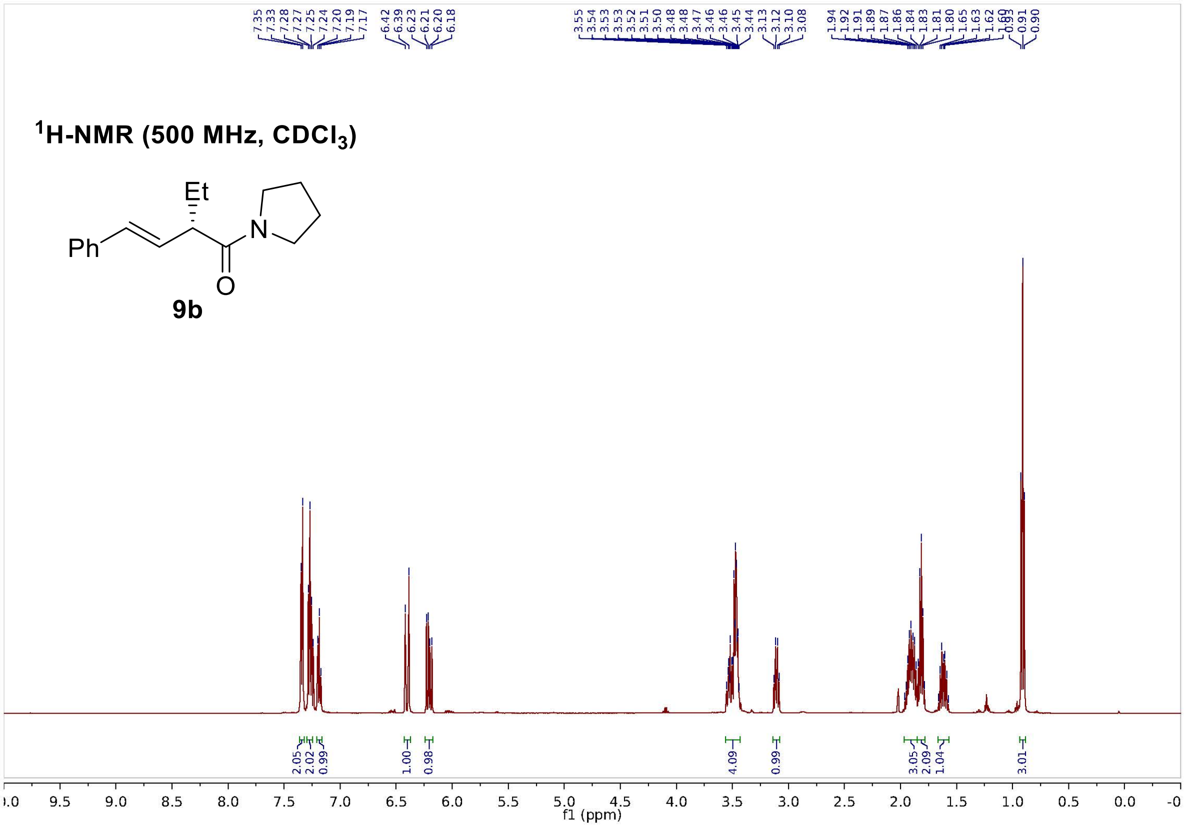
7.35 7.33 7.28 7.27 7.25 7.24 7.20 7.19 7.17
6.42 6.39 6.23 6.21 6.20 6.18
3.55 3.54 3.53 3.53 3.52 3.51 3.50 3.48 3.48 3.47 3.46 3.46 3.45 3.44 3.13 3.12 3.10 3.08
1.94 1.92 1.91 1.89 1.87 1.86 1.84 1.83 1.81 1.80 1.65 1.63 1.62 1.60 0.93 0.91 0.90
1H-NMR (500 MHz, CDCl3)
Et
N
Ph
O
9b
2.05 2.02 0.99 1.00 0.98 4.09 0.99 3.05 2.09 1.04 3.01
f1 (ppm)

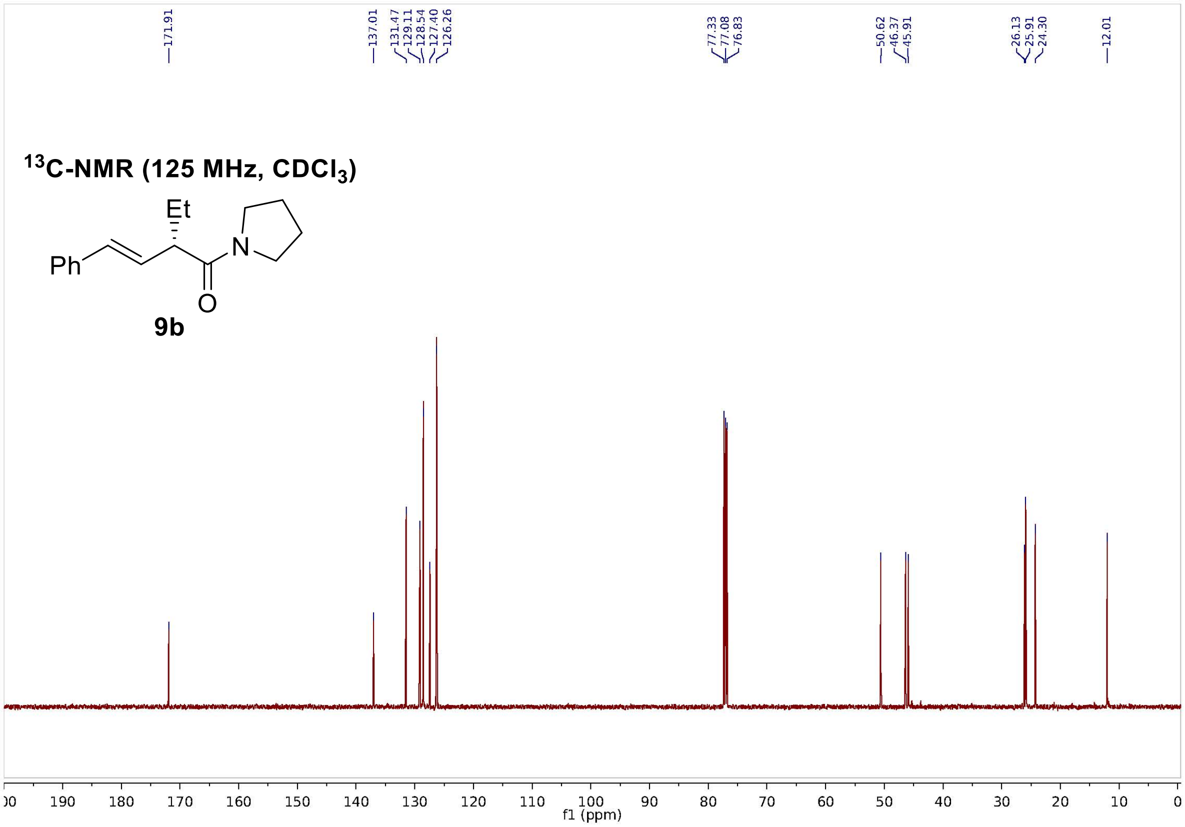
171.91
137.01 131.47 129.11 128.54 127.40 126.26
77.33 77.08 76.83
50.62 46.37 45.91
26.13 25.91 24.30
12.01
13C-NMR (125 MHz, CDCl3)
Et
N
Ph
O
9b
f1 (ppm)
